# Pyridalthiadiazole acceptor-functionalized triarylboranes with multi-responsive optoelectronic characteristics†
†Electronic supplementary information (ESI) available: Detailed synthetic procedures, characterization, NMR spectra, solid state UV-Vis/emission spectra, CV data at different scan rates, and TD-DFT calculation results. CCDC 1493046. For ESI and crystallographic data in CIF or other electronic format see DOI: 10.1039/c6sc03097a


**DOI:** 10.1039/c6sc03097a

**Published:** 2017-06-07

**Authors:** Xiaodong Yin, Kanglei Liu, Yi Ren, Roger A. Lalancette, Yueh-Lin Loo, Frieder Jäkle

**Affiliations:** a Department of Chemistry , Rutgers University – Newark , Newark , NJ 07102 , USA . Email: fjaekle@rutgers.edu; b Department of Chemical and Biological Engineering , Princeton University , Princeton , NJ 08544 , USA; c Andlinger Center for Energy and the Environment , Princeton University , Princeton , NJ 08544 , USA

## Abstract

A new class of Ar_2_B–π–A dyads and A–π–B(Ar)–π–A triads that feature strong organic acceptor moieties (A = pyridalthiadiazole, PT) attached to a central triarylborane were synthesized *via* Stille cross-coupling of ArB(Th–SnMe_3_)_2_ (Th = thiophenediyl, Ar = 2,4,6-tri-*tert*-butylphenyl (Mes*) or 2,4,6-tris(trifluoromethylphenyl) (^F^Mes)) with one or two equivalents of dibromopyridalthiadiazole. Single-crystal X-ray crystallography data for the triad Mes*B(Th–PT–Br)_2_ indicate a highly coplanar conformation, which is ideal for extended π-conjugation and favors intermolecular π-stacking. Despite the presence of Br substituents, these compounds exhibit strong photoluminescence in THF solution with quantum yields reaching up to 52%. Further extension of conjugation by coupling with 2-hexylthiophene leads to additional bathochromic shifts to give a highly soluble and strongly red-emissive material. All these compounds undergo facile reduction, first of the PT substituents and then at more negative potentials for the borane moiety. Upon chemical reduction with  in THF, an intramolecular charge transfer (ICT) pathway from the reduced PT moieties to boron is enabled and this results in a change of the color to blue. Theoretical calculations reveal that, due to the electron-withdrawing effect of the PT moieties, not only the PT-centered LUMOs themselves but also the LUMO+1 or LUMO+2, which show contributions from the p orbital of boron, experience a significant decrease in energy; they are much lower in energy than those of typical conjugated triarylboranes. The relatively low energy of both the PT-centered LUMOs and boron-centered LUMO+1 or LUMO+2 opens up multiple pathways for reaction with highly nucleophilic fluoride anions. Evidence for very strong F^–^ binding to boron is obtained in the case of the more sterically accessible ^F^Mes derivatives. Fluoride anion binding leads to an electron-rich borate moiety and as such generates an ICT pathway to the electron-deficient PT moieties; the direction of this ICT is opposite to that observed upon chemical reduction. For the Mes* derivatives, F^–^ binding is hindered, resulting in competing reduction of the PT acceptors. Finally, the electron acceptor character of the hexylthiophene derivative is exploited in electron-only diodes that show an average electron mobility of 6.4 ± 1.6 × 10^–5^ cm^2^ V^–1^ s^–1^.

## Abstract 2

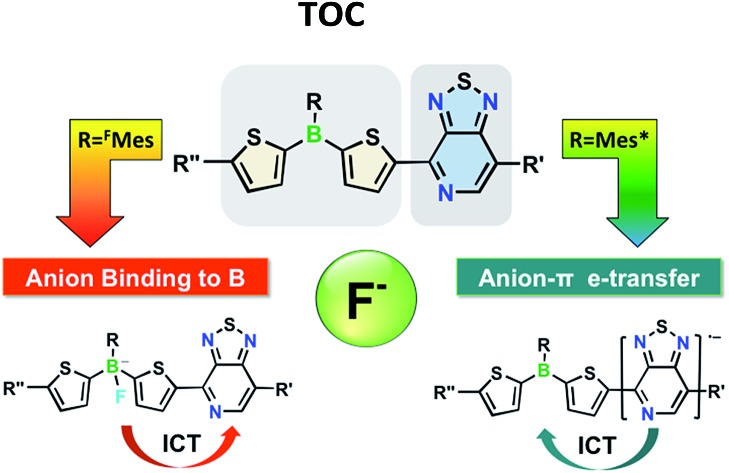
The functionalization of triarylboranes with pyridalthiadiazole (PT) acceptor moieties gives Ar_2_B–π–PT dyads and PT–π–B(Ar)–π–PT triads with low-lying PT-centered LUMO orbitals. Addition of a fluoride source results in competing anion binding to boron and PT reduction, depending on the steric and electronic structure of the B-aryl group.

## Introduction

The incorporation of main-group elements into the backbone of organic conjugated systems has been widely studied over the last decade and, in many instances, attempts to apply these materials in devices have revealed unusual properties and led to improved performances.^1^ Among these conjugated hybrids, boron-containing materials have attracted considerable attention,^2^ in part because interactions between the empty p orbital of boron and π-conjugated systems can lead to desirable optical and electronic properties that in turn enable applications in optoelectronics and sensors.^3^ Another emerging area is the development of switchable materials that respond to an external stimulus, such as irradiation with photons of a specific wavelength, changes in voltage, temperature or solvents, or the addition of chemical triggers.^4^

In prior studies on conjugated organoboranes, boron has typically been embedded into an electron-rich conjugated backbone. For instance, the combination of organoboranes with triarylamines leads to donor–π–acceptor compounds that have been widely explored for non-linear optical, organic light-emitting device (OLED) and sensing applications.^5^ In contrast, the functionalization of organoboranes with π-conjugated electron-acceptors remains sparsely explored.^6^,^7^ We report here a novel series of acceptor–π–acceptor dyads and corresponding triads that feature electron-deficient triarylboranes in combination with pyridal[2,1,3]thiadiazole (PT). PT was chosen because it is a highly electron-deficient heterocycle that has been successfully implemented in narrow band-gap chromophores for organic photovoltaics (OPV).^8^ We demonstrate that the electronic characteristics of both the borane and the PT moieties can be addressed individually using different triggers, such as F^–^ anion binding or chemical/electrochemical reduction, resulting in an intriguing new class of switchable materials. In addition, the acceptor moieties mutually influence each other. The strong electron-withdrawing effect of PT leads to increased Lewis acidity of boron and generates a pathway for ICT from the resulting borate moiety to PT. On the other hand, since PT is more easily reduced than boron, selective electrochemical or chemical reduction gives rise to radical anions and thereby generates a reverse ICT pathway from PT to boron. Further extension of conjugation by transition-metal catalyzed cross-coupling reactions is also demonstrated, suggesting that structural modulation and incorporation into polymeric materials can be readily accomplished.

## Results and discussion

### Synthesis of pyridalthiadiazole–borane dyads and triads *via* Stille coupling

**Scheme 1:**
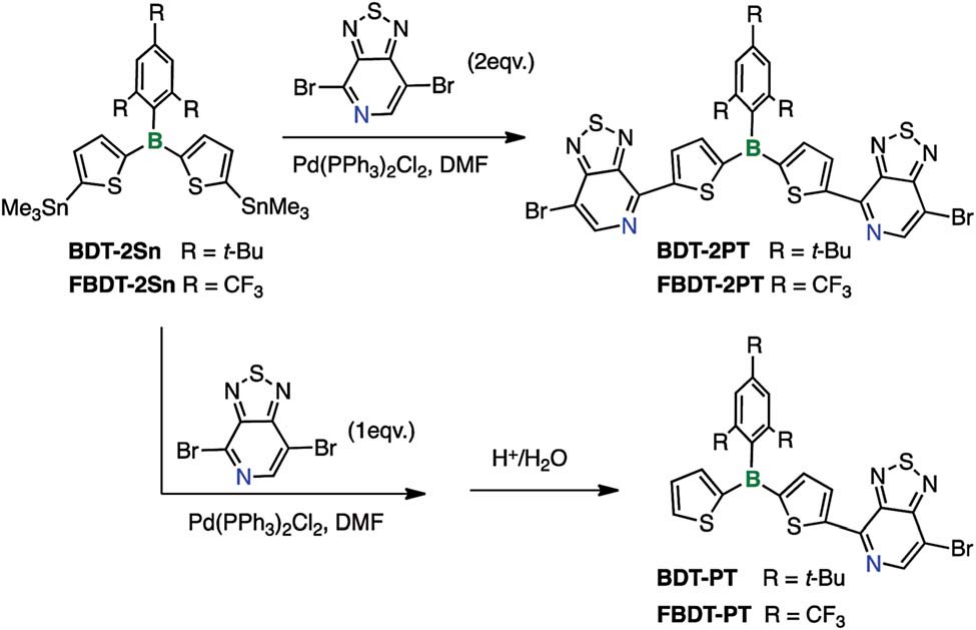
Synthesis of pyridal[2,1,3]thiadiazole acceptor-substituted triarylboranes.

4,7-Dibromo[1,2,5]thiadiazolo[3,4-*c*]pyridine was prepared according to a report by Yamamoto *et al.*^9^ The distannylated compounds, **BDT-2Sn** and **FBDT-2Sn**, were obtained in high yields by dilithiation of **BDT** and **FBDT**, followed by treatment with Me_3_SnCl.^10^ The PT-substituted triarylboranes, **BDT-2PT** (68% yield) and **FBDT-2PT** (55% yield), were then synthesized *via* Stille coupling and isolated by column chromatography in air without any special precaution (Scheme 1). Coupling proceeded with high regioselectivity since the C–Br bond in the *ortho*-position to the pyridyl nitrogen is much more reactive than the one in the *meta*-position. The corresponding mono-functionalized species, **BDT-PT** and **FBDT-PT**, were prepared by similar methods using an equimolar ratio of the reagents (69% and 63% yield, respectively). We note that the products do not contain any stannyl groups, presumably due to destannylation during the column chromatography step.

### Structural characterization

**Fig. 1:**
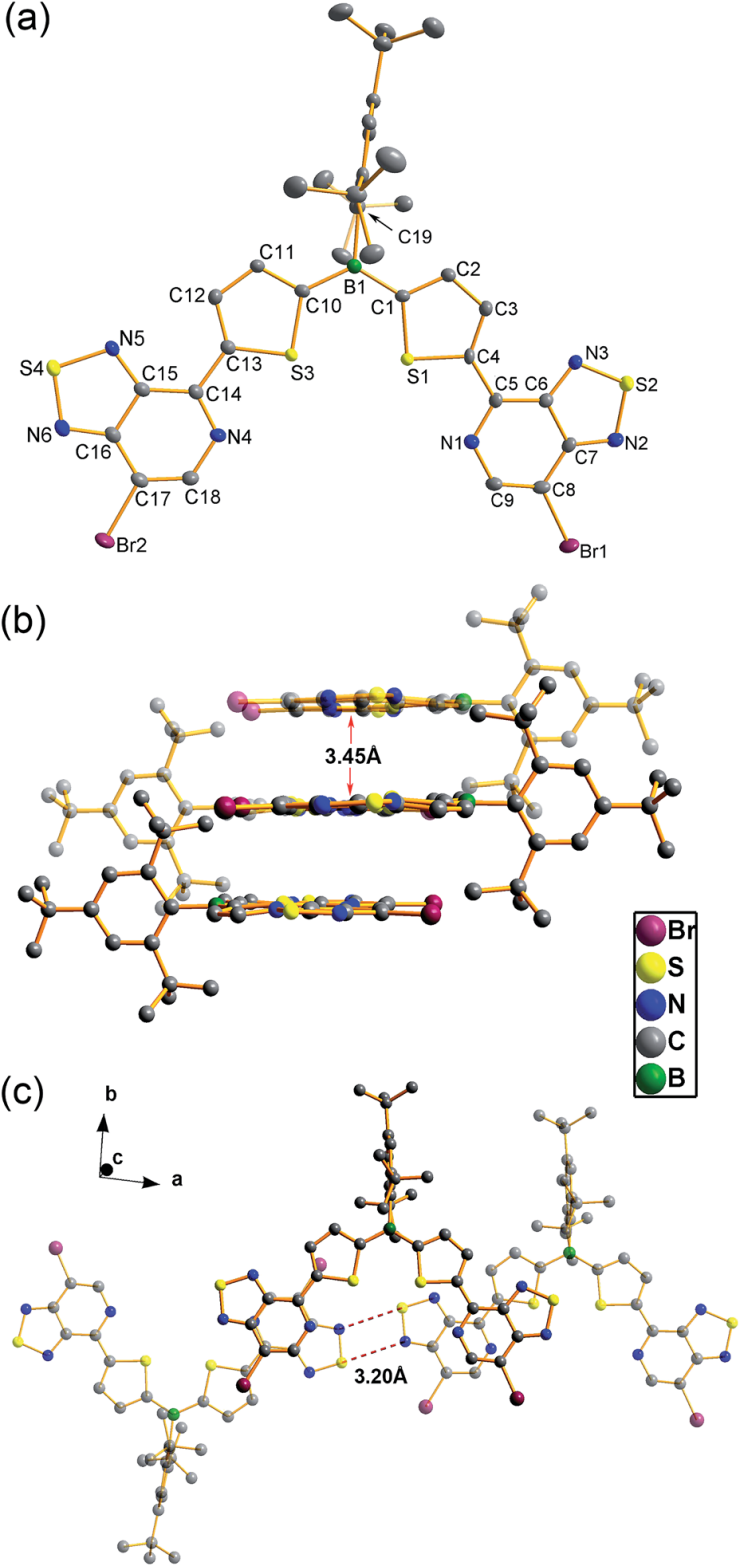
(a) ORTEP plot of the single crystal X-ray structure of **BDT-2PT** (30% thermal ellipsoids, cocrystallized THF molecule and H atoms are omitted); (b) illustration of π-stacking along the crystallographic *c*-axis; (c) illustration of intra-layer interactions.

Single crystals of **BDT-2PT** were obtained by recrystallization from hexanes/THF mixture and the corresponding structure is shown in Fig. 1a. The Mes* group adopts an orientation almost orthogonal to the thiophene rings with dihedral angles of 86.7°.^11^ Due to the “clamp-like” steric effect of the bulky Mes* group, the thiophene rings and the boron atom adopt a quasi-planar structure with a small torsion angle of 9.1° between the thiophene rings. The thiophene and pyridalthiadiazole rings are also coplanar with torsion angles of only 7.0 and 3.0°, indicating planarity over the entire PT–Th–B–Th–PT skeleton. The coplanar structure is expected to favor extended π-conjugation along the main chain *via* the empty p orbital on boron. The packing motif of **BDT-2PT** (Fig. 1b) reveals π-stacking along the *c*-axis with short distances of *ca.* 3.45 Å between PT heterocycles. Stacking is only observed for the PT heterocycles, whereas the central **BDT** moieties are separated by THF solvent molecules. The aromatic rings of the conjugated main chains form layers that alternate with layers containing the Mes* groups (Fig. 1b and c). Short distances between electron-rich sulfur and electron-deficient nitrogen atoms (average N···S distance of 3.20 Å *vs.* sum of van der Waals radii of 3.35 Å (ref. 12)) within these layers generate a 1D ribbon-like structure of conjugated molecules perpendicular to the π-stacking direction.

### Photophysical properties of PT–borane dyads and triads

**Fig. 2:**
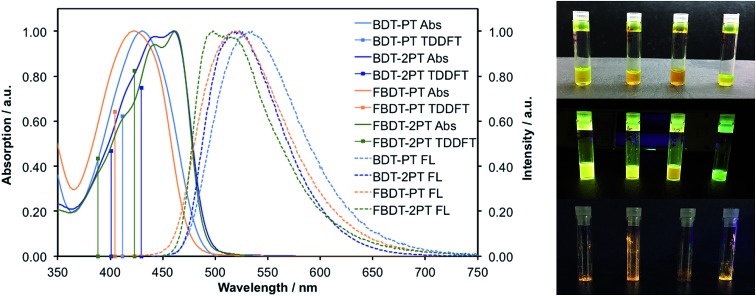
Normalized UV-Vis and fluorescence spectra of borane–PT dyads and triads (1 × 10^–5^ M in THF). Solid drop lines correspond to TD-DFT calculation results. Photographs of (top) solutions in THF under natural light, (middle) solutions in THF under UV irradiation, (bottom) solid samples of **BDT-PT**, **BDT-2PT**, **FBDT-PT**, and **FBDT-2PT** under UV irradiation (left to right).

The photophysical properties in THF solution are summarized in Table 1 and the spectral data are provided in Fig. 2. Generally, the triads give rise to longer wavelength absorption maxima (*ca.* 440–470 nm) than the corresponding dyads (*ca.* 420 to 430 nm), which is attributed to a more extended π-conjugated skeleton in the former. Despite the presence of Br substituents on the PT moieties all the compounds are highly fluorescent, with emission wavelength maxima in the range of 495 to 535 nm and quantum yields from 32 to 52%. In contrast to the trends in the absorption data, the emission for the dyads and triads occurs in a similar wavelength range, resulting in much larger Stokes shifts for the dyads. This observation is attributed to differences in the ICT character of the absorption bands, as further discussed *vide infra*. Thin-film photophysical data show consistent bathochromic shifts in the absorption and emission maxima, indicative of intermolecular interactions and/or planarization of the backbone (Fig. S1†). The thin-film quantum yields for **BDT-PT** and **BDT-2PT** were in the range of 12–18%. In both solution and thin film, the bis-PT triads show consistently lower quantum yields, which might be related to more facile non-radiative decay resulting from rotation of the additional PT ring or enhanced inter-system crossing^13^ due to the presence of a second Br heavy atoms. This is consistent with time-resolved measurements in THF solution that reveal a shorter lifetime for the triads relative to the dyads (Fig. S2†). Besides, the fluorinated compounds exhibit shorter fluorescence lifetimes (with biexponential characteristics) and both larger radiative and non-radiative rate constants than the corresponding non-fluorinated species (Table 1).

**Table 1:** Photophysical data of borane–PT dyads and triads

Compound	*λ*_abs(THF)_/nm	*λ*_abs(solid)_ ^*a*^/nm	*λ*_em(THF)_/nm	*λ*_em(solid)_ ^*a*^/nm	*Φ*_F(THF)_	*Φ*_F(solid)_ ^*a*^	*τ*/ns	*k*_r_ ^*b*^/10^8^ s^–1^	*k*_nr_ ^*b*^/10^8^ s^–1^
**BDT-PT**	430	442	535	578	52 ± 0.2%	18 ± 5%	7.8 ± 0.01	0.6	0.7
**BDT-2PT**	460, 443	491	523	595	32 ± 0.7%	12 ± 5%	3.6 ± 0.01	0.9	1.6
**FBDT-PT**	422	437	519	602	52 ± 0.1%	^*c*^	5.3 ± 0.01^*d*^	1.0	0.9
**FBDT-2PT**	462, 442	497	498, 515	622	38 ± 0.1%	^*c*^	2.0 ± 0.01^*d*^	1.9	3.1

^*a*^Thin film data (see Fig. S1).

^*b*^ *k*_r_ = *Φ*/*τ*, *k*_nr_ = 1/*τ* – *k*_r_.

^*c*^Signal too weak to determine.

^*d*^Average value for biexponential decay.

### Redox properties of PT–boranes

**Fig. 3:**
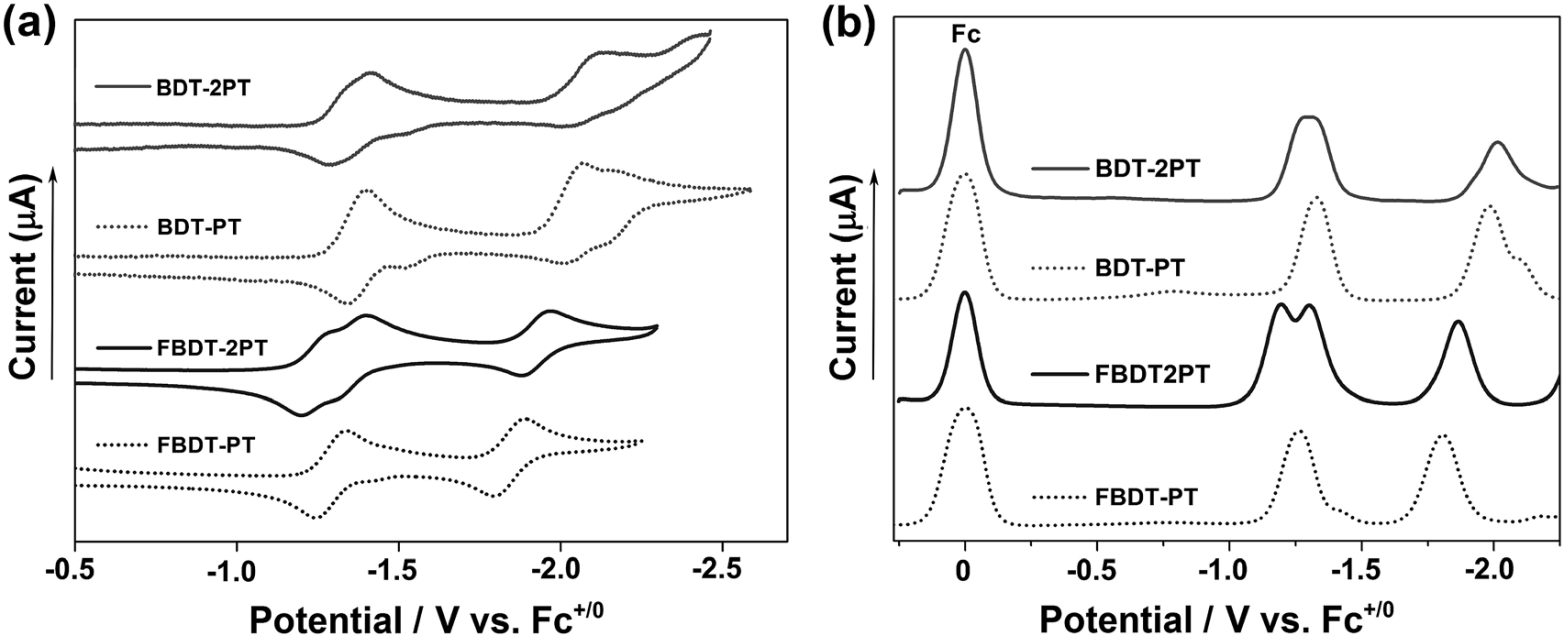
(a) Cyclic voltammetry (CV) and (b) square wave voltammetry (SWV) data in THF/0.1 M Bu_4_N[PF_6_] (1 × 10^–3^ mol L^–1^; *vs.* Fc^0/+^, *ν* = 100 mV s^–1^).

Cyclic voltammetry (CV) and square wave voltammetry (SWV) experiments conducted in THF solution reveal multiple reduction processes within the potential range from –1.2 to –2.1 V *vs.* Fc^+/0^ (Fig. 3 and S3†). By comparison with electrochemical data for the individual components,^11^ the redox waves in the range from –1.2 to –1.4 V are attributed to initial reduction of the PT rings, and those at –1.8 to –2.1 V correspond to reduction at the boron centers (Table S1†). The reduction potentials are much less cathodic than those for the respective precursors, **BDT** (–2.58 V) and **FBDT** (–2.22 V),^11^ demonstrating the strong electron-withdrawing effect of the PT heterocycle. Further, it is remarkable that the boron-centered reductions occur more readily for the PT-substituted derivatives despite the formation of radical anions and dianions in the initial reductions centered on the PT moieties. As expected, they occur at significantly less negative potential for the fluorinated compounds.^14^ Interestingly, a larger redox splitting of the PT-centered redox processes in **FBDT-2PT** (Δ*E* = 0.11 V) relative to those of **BDT-2PT** may also indicate more effective electronic communication between PT moieties as the organoborane “bridge” becomes more electron-deficient.

### Theoretical studies of neutral PT–boranes

Geometry optimization at the B3LYP/6-31+G* level of theory (Fig. S4†) reproduced the quasi-planar structure of the PT–Th–B–Th–PT skeleton with very small twist angles between the thiophene rings (1.6° for **BDT-PT**, 2.5° for **BDT-2PT**, 20.5° for **FBDT-PT**, and 15.1° for **FBDT-2PT**), consistent with the results of the X-ray structure analysis of **BDT-2PT**. The larger twist angle in **FBDT-PT** and **FBDT-2PT** may be due to the lower steric demand of the ^F^Mes in comparison to the Mes* group. On the other hand, the dihedral angles between the thiophene and PT groups are as small as 0.2° in all compounds, indicating favorable planarity and conjugation along the backbone.

**Fig. 4:**
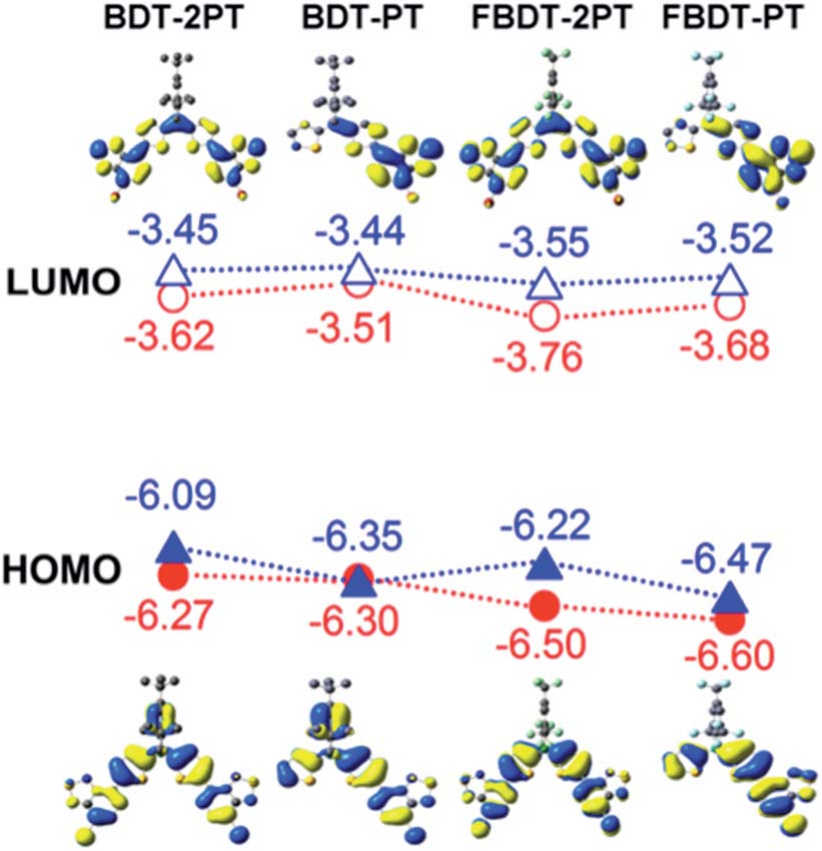
HOMO and LUMO orbitals of borane–PT dyads and triads (structures optimized at B3LYP/6-31+G* level, single point energy calculations at B3PW91/6-311+G**). Red circles correspond to DFT results, blue triangles to experimental results using *E*_LUMO_ = –4.8 – *E*_red_ (eV), *E*_gap_ = 1240/*λ*_onset_ (eV).

DFT calculations further reveal that the HOMO is mainly composed of bonding orbitals localized on the thiophenes and the pyridyl rings of the PT groups, and the LUMO is mainly composed of anti-bonding orbitals of the PT groups (Fig. 4). The boron p orbital makes a significant contribution to the LUMO (but not the HOMO), resulting in conjugation over the entire backbone. Consistent with the electrochemical data, the strongest contributions of the boron p orbital are seen in the LUMO+1 for **BDT-PT** and **FBDT-PT** and the LUMO+2 for **BDT-2PT** and **FBDT-2PT** (Table S2†). The benzene ring of the Mes* group participates in the HOMO of **BDT-PT** and **BDT-2PT**, but this is not the case for the ^F^Mes group, because of the strong electron-withdrawing effect of the trifluoromethyl groups.

To gain insights into the nature of the electronic transitions for the new organoboranes TD-DFT calculations were conducted at the ωB97XD theory level with a 6-311+G** basis set for all elements. As seen in Fig. 2 (drop lines), the results from these TD-DFT calculations match well with the experimental absorption spectra that show a single absorption maximum for **BDT-PT** and **FBDT-PT** at *ca.* 420 to 430 nm and two overlapping absorption maxima for **BDT-2PT** and **FBDT-2PT** in the range of 440–462 nm. The single absorption for the dyads is predicted to be mainly due to a S_0_ → S_1_ transition from the HOMO to a delocalized LUMO with contributions from the boron p orbital (Table S3†). For the corresponding triads, the two absorption bands can be ascribed to S_0_ → S_1_ (HOMO → LUMO) and S_0_ → S_2_ (HOMO–1 → LUMO/HOMO → LUMO+1) transitions (Table S4†), where the LUMO is delocalized over the entire skeleton including the boron atom, but the LUMO+1 is mostly localized on the PT acceptor units. All these low energy bands are primarily π–π* in nature; ICT processes to boron-centered p orbitals are observed at much higher energy.

### Chemical reduction of **BDT-PT** and **BDT-2PT**

**Fig. 5:**
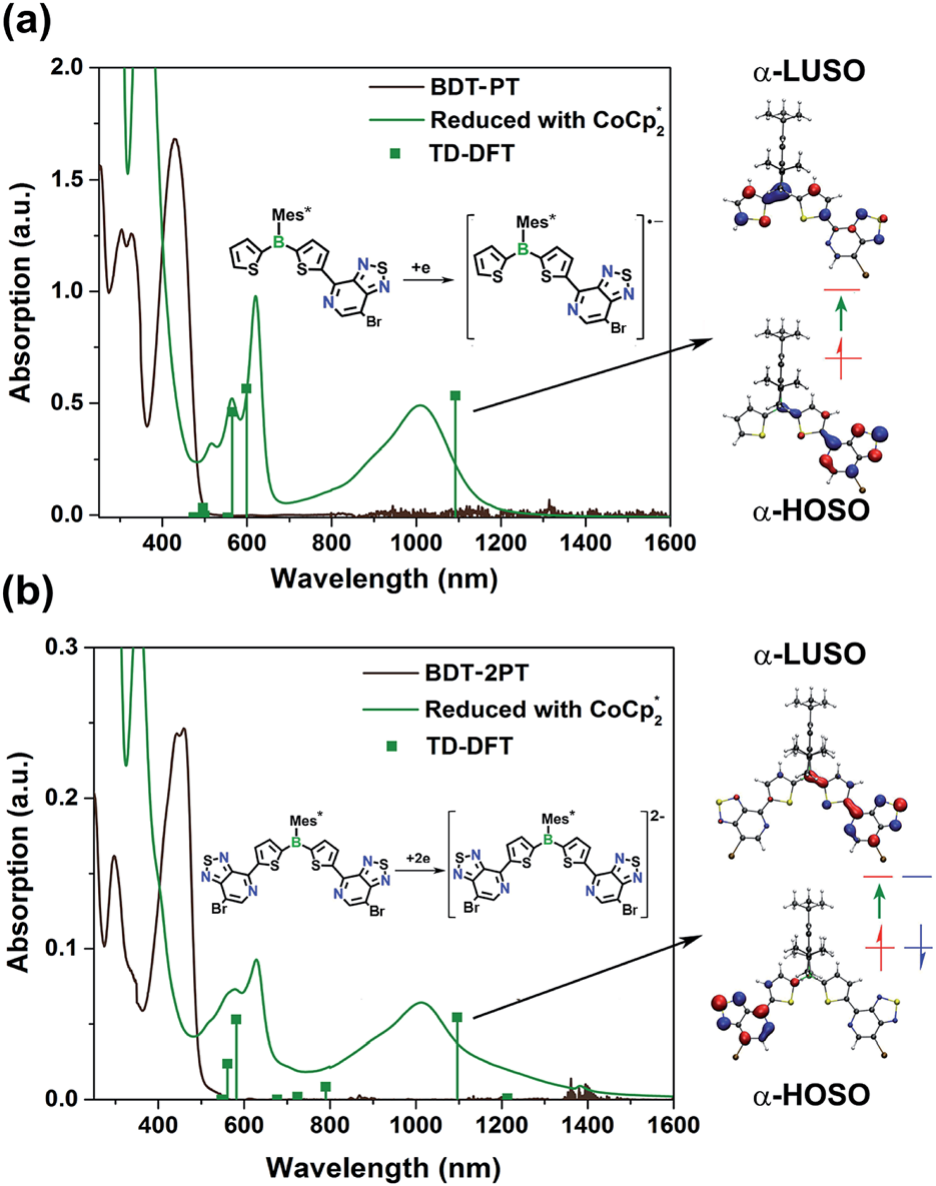
UV-Vis spectra of (a) **BDT-PT** and (b) **BDT-2PT** before (black) and after (green) chemical reduction with 
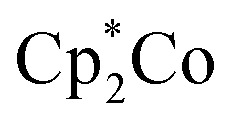
 in THF. Droplines with squares correspond to the TD-DFT results for the radical anion/dianion. Right: Illustration of electronic transitions of **BDT-PT˙^–^** and **BDT-2PT^2–^** corresponding to the lowest energy absorptions at *ca.* 1000 nm. Peaks at <400 nm are due to excess 
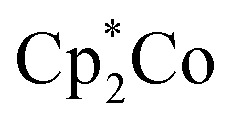
.

Our electrochemical and theoretical studies reveal that the first reduction processes take place on the PT moieties. This observation is in stark contrast to typical arylboranes,^15^ and the chemical reduction might therefore trigger a change in the photophysical properties with emergence of new ICT bands. As shown in Fig. 5, upon reduction of **BDT-PT** and **BDT-2PT** with an excess amount of decamethylcobaltocene 
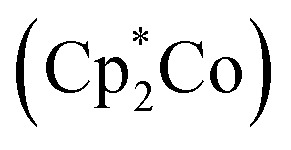
 in THF, new broad bands emerge in the near-IR region at *ca.* 1020 nm, accompanied by absorptions at *ca.* 550 to 630 nm, to give the compounds a dark blue appearance. Very similar absorptions are observed for the radical anions and dianions generated from the fluorinated species **FBDT-PT** and **FBDT-2PT**, except for that the longest wavelength band is shifted to *ca.* 1110 nm as illustrated in Fig. S5.†

We conducted DFT calculations on the radical anion **BDT-PT^–^˙** and dianion **BDT-2PT^2–^** at the uB3LYP/6-31+G* level of theory. The results indicate that the α-HOSO (Highest Occupied Spin Orbital) of **BDT-PT^–^˙** is mainly located on the PT moiety (Fig. S6†), which is consistent with the calculated distribution of spin density (Fig. S7†). The broad low-energy absorption at *ca.* 1020 nm can then be ascribed to charge transfer from the electron-rich [PT]˙^–^ moiety (α-HOSO) to the electron-deficient thienylborane moiety (α-LUSO, Lowest Unoccupied Spin Orbital). The higher energy absorptions at *ca.* 550 to 600 nm are due to transitions mostly from β-HOSO–1 to β-HOSO and α-HOSO to α-LUSO+2, and thus primarily involve thiophene and PT-centered orbitals with little contribution of boron. For the doubly reduced triad **BDT-2PT^2–^**, an unrestricted, broken-symmetry singlet solution that is lower in energy by *Δ* = 0.33 eV than the restricted solution indicates a biradicaloid character for the lowest singlet state. The electrons in the α-/β-HOSO are therefore localized on each of the PT moieties, which is again consistent with the calculated spin density (Fig. S7†). TD-DFT calculations for **BDT-2PT^2–^** are in good agreement with the experimental results although the transitions are much more complex than for the mono-PT compound. Similar to the mono-PT dyad, the broad absorption at *ca.* 1000 nm is ascribed to charge transfer from the electron-rich [PT]˙^–^ moieties to the electron-deficient boron–Th–PT moiety on the other side of the molecule (HOSO → LUSO), as shown in Table S6 and Fig. S6.† The higher energy absorptions at *ca.* 550 to 600 nm are due to transitions involving pyridylthiophene-centered orbitals.

### Anion binding properties

**Fig. 6:**
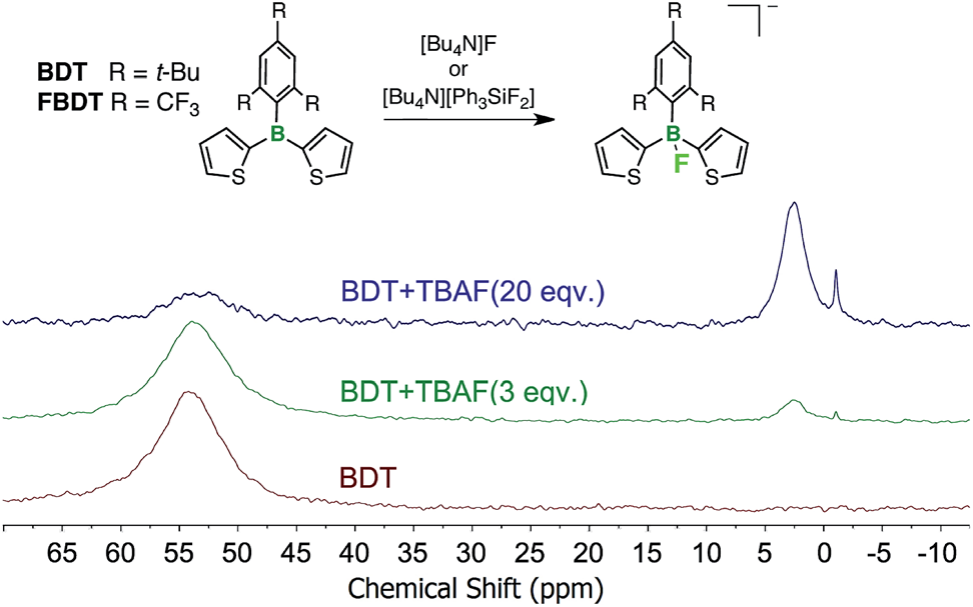
Fluoride anion binding to **BDT** and **FBDT**; changes in the ^11^B NMR data for **BDT** (2.0 × 10^–2^ mmol in 0.55 mL d8-THF) after addition of TBAF·3H_2_O (2.0 × 10^–1^ M in d8-THF) are shown.

According to the electrochemical data, the boron-centered reductions of the PT–borane dyads and triads occur at significantly lower potential than for the respective borane compounds without PT-acceptor functionalization. If sterically accessible, one would expect the increased electron-deficient character to also result in enhanced Lewis acidity of the boranes.^16^ In prior work, based on UV-Vis titrations, we concluded that the parent Mes*-substituted borane, **BDT**, does not bind F^–^ anions, but the corresponding fluorinated species **FBDT** acts as a strong receptor with a binding constant of lg *K* ≥ 7 in THF (∼1–2 × 10^–5^ M).^11^ These findings were consistent with Yamaguchi's work on a Mes*-substituted dibenzoborole.^17^ A reinvestigation by NMR spectroscopy in d8-THF suggested partial fluoride anion binding (*ca.* 65% binding) to **BDT** when subjected to a 20-fold excess of TBAF·3H_2_O (TBAF = [Bu_4_N]F) at the much higher concentration (∼2 × 10^–2^ M) used for the NMR experiments (Fig. 6, see also Fig. S8†). New signals at +2.5 ppm in the ^11^B NMR and at –130.9 ppm in the ^19^F NMR spectrum are attributed to fluoroborate formation. In addition, all the aromatic protons experience a characteristic upfield shift (*δ* = 7.07 (Mes*), 6.95 (Th), 6.86 (Th), 6.71 ppm (Th)), consistent with the increased electron-density at boron. Similar upfield shifts had been previously observed upon fluoride binding to **FBDT**, while the ^11^B NMR resonance was found at 1.7 ppm and the ^19^F NMR resonance for the boron-bound fluorine more upfield shifted at –151 ppm.^11^ Nevertheless, fluoride anion binding to **BDT** is clearly several orders of magnitude weaker than for **FBDT**.

Next, we conducted anion binding experiments on the new PT-substituted boranes to gain insights into the influence of the PT acceptor moieties on the Lewis acid properties. Initial studies on the fluoride binding to **FBDT-PT** and **FBDT-2PT** with TBAF·3H_2_O in THF under N_2_ atmosphere indicated very strong binding of F^–^ with a lg *K* ≥ 7 based on a UV-Vis titration in THF (Fig. S9†). The corresponding anion complexes were detected by ESI mass spectrometry (Fig. S10 and S11†). However, a peculiarity in the titration experiments was observed in that the initial binding was unexpectedly weak leading to very moderate decreases in the absorption. A possible explanation could be that the small amounts of water present in TBAF·3H_2_O led to retardation of the anion binding. Moreover, addition of excess BF_3_·OEt_2_ did not lead to quantitative regeneration of the corresponding free Lewis acids, suggesting that the borate complexes undergo further reaction. NMR spectral data showed evidence of free 1,3,5-tris(trifluoromethyl)benzene, which can be attributed to hydrodeborylation. Furthermore, for **FBDT-2PT** MALDI-TOF MS data suggested the formation of a by-product at twice the mass of the bromopyridal-thiadiazolylthiophene moiety, indicative of coupling of the substituents with expulsion of boron (Fig. S12†). When attempting to perform these studies under more rigorous oxygen-free conditions in a glove box, we also found evidence for radical formation (*vide infra*).

**Fig. 7:**
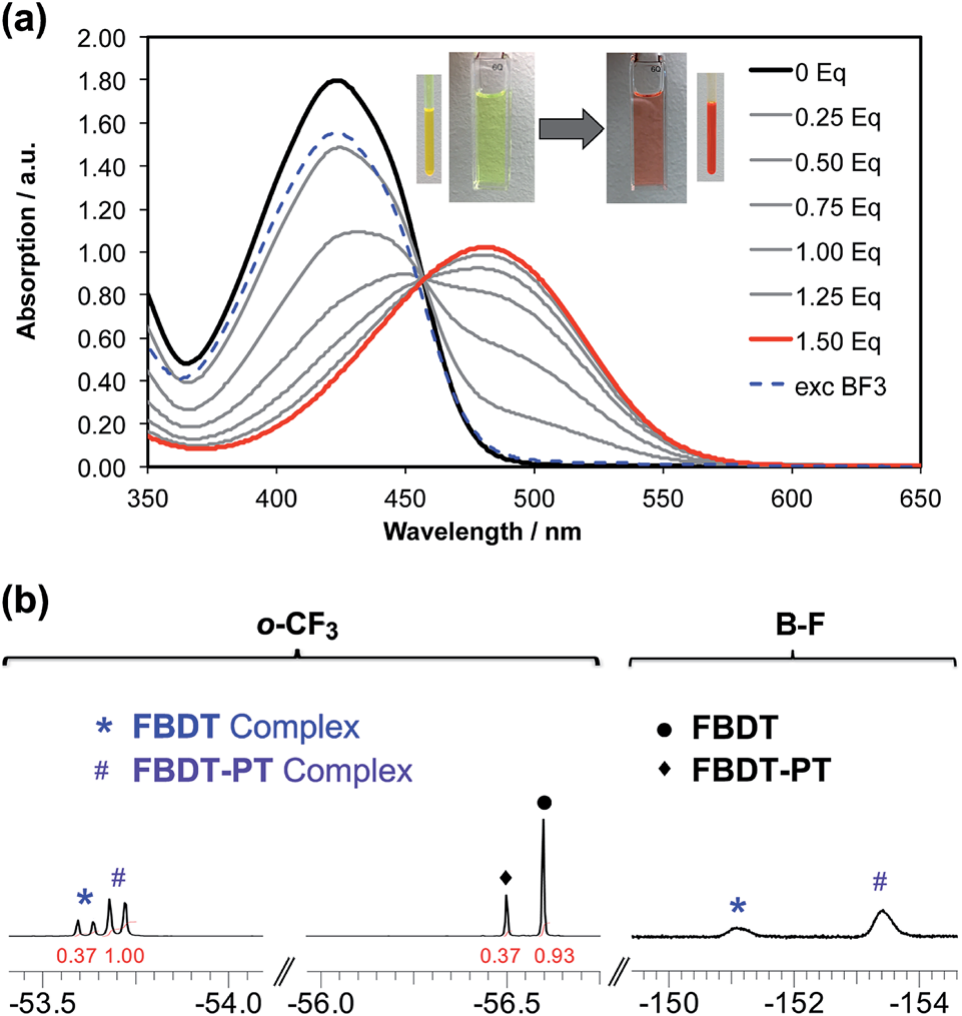
(a) Plots illustrating the UV-Vis titrations of **FBDT-PT** and in THF solution. Aliquots of a [Bu_4_N][Ph_3_SiF_2_] solution (5.0 × 10^–3^ M in THF) were added to a solution of the organoborane Lewis acid (2.4 × 10^–4^ mmol in 3 mL THF); the binding was reversed by addition of an excess amount of BF_3_·OEt_2_. (b) ^19^F NMR data in d8-THF illustrating competitive binding of **FBDT-PT** and **FBDT** with [Bu_4_N][Ph_3_SiF_2_] (1 : 1 : 1 molar ratio; [**FBDT-PT**]^0^ = [**FBDT**]^0^ = 5.0 × 10^–3^ M); *p*-CF_3_ not shown.

This prompted us to perform additional anion binding studies with the difluorosilicate [Bu_4_N][Ph_3_SiF_2_] as an isolable anhydrous fluoride source. Multinuclear NMR studies in d8-THF confirmed the formation of a fluoride complex of **FBDT-PT** with peak patterns that are comparable to those observed for the corresponding complex of the parent compound **FBDT** (Fig. S13†). The color of the solution changed from light yellow to intense orange and a UV-Vis spectral titration gave a binding constant of *K* = 4.5 × 10^5^ M^–1^ (Fig. 7a and S14†), which is remarkably high when considering that the binding equilibria also involve decomplexation of F^–^ from Si. We note that there is a significant kinetic barrier, which makes it imperative to let the mixture equilibrate for *ca.* 30 minutes after each addition of the fluoride source. Importantly, according to a ^19^F NMR competition experiment (Fig. 7b), anion binding is much stronger for **FBDT-PT** relative to **FBDT**, indicative of enhanced Lewis acid strength of **FBDT-PT**.

The respective Mes*-substituted boranes **BDT-PT** and **BDT-2PT** are much more sterically hindered and, not surprisingly, we found no evidence of anion binding or any other reactivity even in the presence of a very large excess of [Bu_4_N][Ph_3_SiF_2_] as the fluoride source under otherwise similar conditions. However, when an excess amount of TBAF·3H_2_O was added to a THF solution of **BDT-PT** or **BDT-2PT** under strict exclusion of oxygen, we observed an unexpected change of color from yellow to blue-green. UV-visible spectra showed new absorptions with maxima in the range of *ca.* 600 to 650 nm (Fig. S15†). In addition, very broad and weak absorptions were detected in the near-IR region for **BDT-2PT**. We note that similar spectral features are also observed for **FBDT-PT** and **FBDT-2PT** when treated with TBAF as the anion source (Fig. S16†). These absorptions somewhat resemble those observed upon reduction of the borane species with 
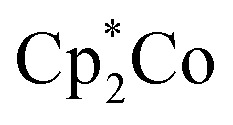
. This then raises the question whether reduction with formation of PT-centered radical anions might compete with the anion binding to boron.

**Fig. 8:**
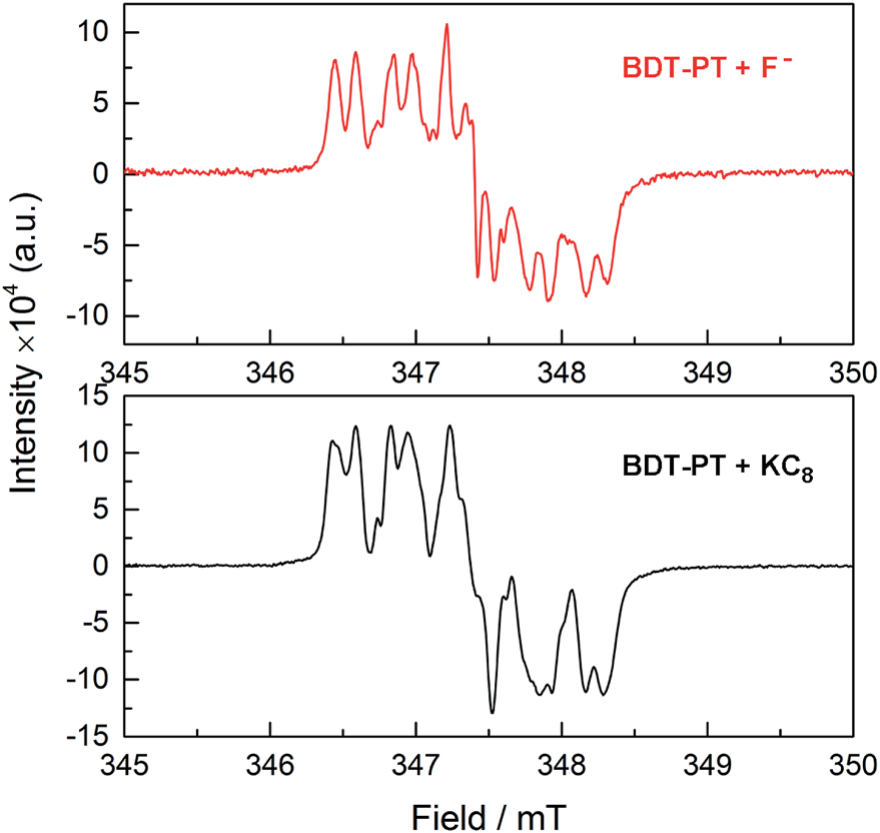
(Top) EPR data after addition of TBAF·3H_2_O to a solution of **BDT-PT** in THF; (bottom) EPR data after addition of a small amount of KC_8_ to a solution of **BDT-PT** in THF.

It is important to remember in this context that the electrochemical and DFT studies reveal that the LUMO levels are localized primarily on the PT moieties and the boron-centered unoccupied orbitals are found at significantly higher energy. Saha and coworkers showed that addition of strongly Lewis basic anions to electron-deficient naphthalene diimides (NPI) leads to anion–π interactions that ultimately result in reduction and generation of the corresponding [NPI]˙^–^ radical anions.^18^ It seems plausible then that in the case of **BDT-PT** and **BDT-2PT**, where anion binding to boron is sterically hindered by the very bulky substituent, reduction of the compounds by F^–^ effectively competes with the anion binding to boron. This process would be less favorable when using [Bu_4_N][Ph_3_SiF_2_] as the F^–^ source due to the diminished reducing power of the fluorosilicate complex. Preliminary EPR studies confirmed the formation of a radical species upon addition of TBAF to **BDT-PT** with similar spectral features as in the case of reduction (Fig. 8). Evidence of competing processes is also found from NMR binding studies in d8-THF performed under strict exclusion of oxygen. Partial binding of F^–^ to **BDT-2PT** is evidenced by the emergence of an upfield-shifted resonance at *ca.* +1 ppm in the ^11^B NMR spectrum and a broad signal at *ca.* –152 ppm in the ^19^F NMR spectrum (Fig. S17 and S18†). At the same time the ^11^B NMR resonance for the uncomplexed **BDT-2PT** and the corresponding ^1^H NMR data show evidence of extreme signal broadening. Upon addition of excess BF_3_·OEt_2_, the resonances attributed to the fluoroborate complex disappeared and the broad ^11^B NMR resonance for the free Lewis acid at *ca.* 57 ppm reemerged.

Coming back to the effect of anion binding to boron on the photophysical properties, it is clearly evident from Fig. 6 (and S9†), that upon adding an excess amount of fluoride the longest wavelength absorption bands experience a distinct red shift from 420 (**FBDT**)/462 nm (**FBDT-2PT**) to *ca.* 480–490 nm, while the emission is quenched concurrently. This phenomenon is unusual in that typically the binding of fluoride anions to conjugated organoboranes leads to a blue-shift of the absorption maximum due to diminished π-conjugation.5g,^19^ We thus surmise that the unusual acceptor (A)–π–boron structure in our compounds must enable a new low energy ICT pathway from the electron-rich borate moiety to the PT acceptor moieties upon anion binding. TD-DFT calculations confirm this hypothesis (Tables S7–S9†). For the dyads, the excitation can be assigned to a S_0_ → S_1_ transition, which corresponds to ICT from the electron-rich thienyl (and Mes*) groups on boron to PT acceptor-centered orbitals. In the triads, the excitation is the result of two transitions, S_0_ → S_1_ and S_0_ → S_2_, which correspond to (HOMO–1/HOMO) → (LUMO/LUMO+1), indicating a similar ICT process from thienyl (and Mes*) donor to PT acceptor-centered orbitals. Combined with the charge-transfer phenomena observed in the chemical reduction processes, we have thus demonstrated that the PT and borane acceptors in the A–π–boron/A–π–boron–π–A system can be modulated *via* reduction and anion binding, respectively, to generate distinct ICT pathways.

### Band gap engineering *via* further extension of π-conjugation

**Fig. 9:**
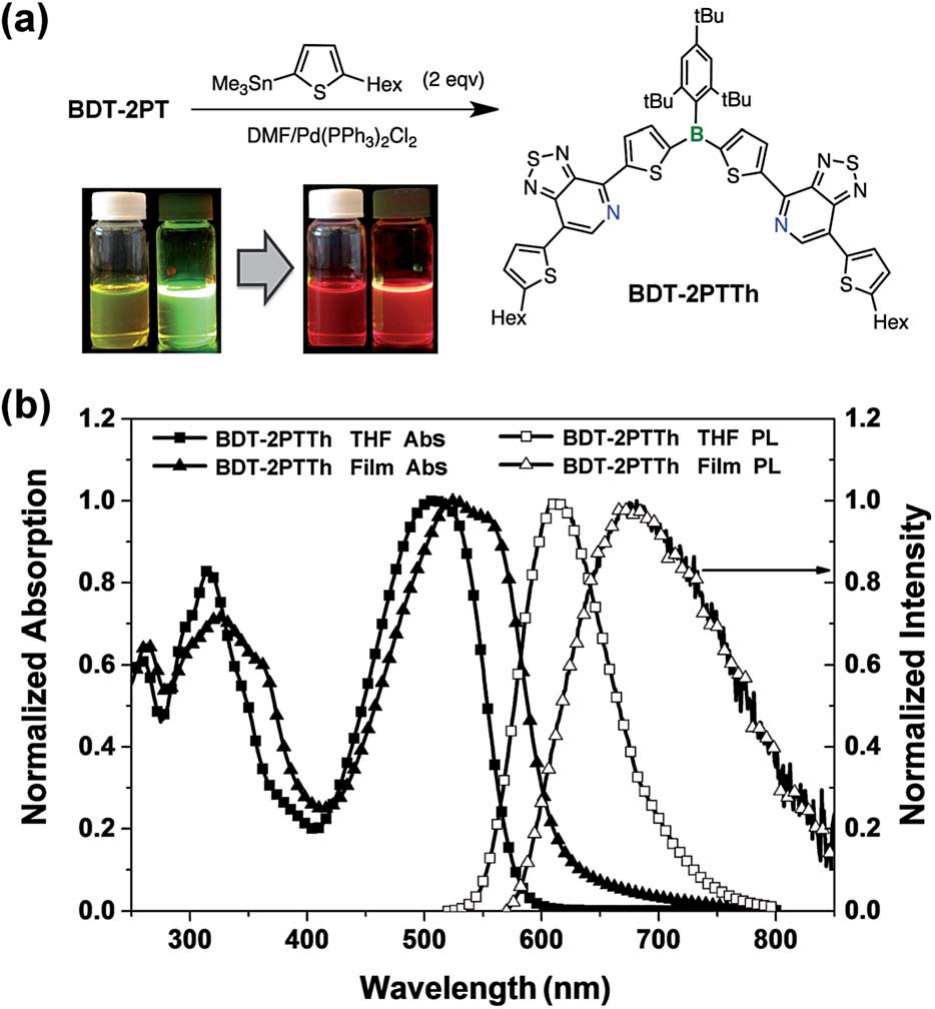
(a) Synthesis of **BDT-2PTTh** (Hex = *n*-hexyl) and corresponding photographs of DCM solutions under natural light and upon irradiation with a UV lamp (365 nm); (b) UV-Vis and photoluminescence spectra of **BDT-2PTTh** in THF and as thin film.

The PT acceptor-substituted organoborane building block can be further extended/functionalized by exploiting the bromine end groups to fine-tune the band gap and to improve materials processing characteristics, such as thermal stability and solubility. Stille coupling of **BDT-2PT** with 5-hexyl-2-trimethylstannylthiophene in DMF gave the expected product, **BDT-2PTTh**, as a dark red solid in 86% yield after purification by column chromatography (Fig. 9). Upon excitation at the absorption maximum of 495 nm in THF solution, **BDT-2PTTh** exhibits a strong red emission with a maximum at 610 nm and a remarkable quantum yield of 68 ± 2%. In thin films, both the UV-Vis and fluorescence bands are further red shifted (40 nm in the absorption and 80 nm in the emission) and the solid-state quantum yield decreases dramatically to 6 ± 1% due to π–π stacking effects. Besides, the extended π-conjugation also results in a longer fluorescence lifetime (7.1 ns in THF, Fig. S20†) than for **BDT-2PT** (3.6 ns, see Table 1). The oxidation (in THF) and reduction (in DCM) processes for **BDT-2PTTh** exhibit electrochemical reversibility with the first reduction and oxidation half-wave potentials at –1.42 V and +0.71 V (*vs.* Fc^+/0^), respectively (Fig. S21–S23†).^20^ In comparison to **BDT-2PT**, the HOMO of **BDT-2PTTh** (–5.57 eV) is shifted to higher energy due to the extension of conjugation into the terminal thiophene rings. This effect is less pronounced for the LUMO (–3.29 eV), which explains the observed bathochromic shifts in the absorption and emission bands.

### Application in electron-only diodes

Finally, to explore the potential utility of **BDT-2PTTh** as a new acceptor-type device material, we built electron-only diodes by spin coating a solution of **BDT-2PTTh** in CHCl_3_ (10 mg mL^–1^) and using ITO and Al as electrodes. We extracted an average electron mobility of 6.4 ± 1.6 × 10^–5^ cm^2^ V^–1^ s^–1^ based on three devices (Fig. S24†). This electron mobility is close to that reported by Yamaguchi for vacuum-deposited borane-functionalized thienylthiazoles^21^ (1.5 × 10^–4^ cm^2^ V^–1^ s^–1^), but lower than that of a planarized triphenylborane with mesogenic side groups that facilitate formation of columnar stacks^22^ (*ca.* 10^–3^ cm^2^ V^–1^ s^–1^). The lower mobility in our case is likely due to the amorphous nature (Fig. S25†) of the solution-deposited thin films of **BDT-2PTTh**.^23^

## Conclusions

In this work, we successfully synthesized a series of borane compounds that are functionalized with PT electron-acceptor groups. These compounds exhibit remarkable stability to air and water and are strongly fluorescent in solution with relatively high quantum yields. The single-crystal structure of **BDT-2PT** reveals planarity of the main skeleton and strong intermolecular π–π stacking with inter-layer distances of 3.45 Å, and similar planar structures are predicted for all other derivatives using DFT calculations. Functionalization with PT electron-acceptor units dramatically lowers both the PT-centered LUMO and boron-centered LUMO+1/LUMO+2 orbitals. As a result, competing anion binding and reduction processes are enabled upon addition of fluoride as a Lewis base. The reduction processes are most prevalent for the highly hindered Mes* derivatives. In contrast, selective and reversible F^–^ binding is achieved upon treatment of the highly Lewis acidic and less hindered **FBDT-PT** derivative with [Bu_4_N][Ph_3_SiF_2_] as the anion source. This binding process can be monitored by naked eye because of the strong red shift of the UV-Vis absorption, which is due to ICT from the newly generated electron-rich borate moieties to the electron-deficient PT moiety. An entirely opposite charge-transfer pathway is generated *via* chemical reduction with 
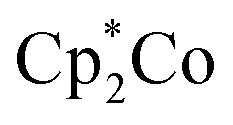
, indicating that the electronic characteristics of the PT and borane moieties can be addressed independently to achieve molecular switching properties. The reactivity of the bromine terminal substituents was exploited in a coupling reaction with stannylated hexylthiophene. The additional π-extension results in a well soluble compound that displays strong red emission and excellent electrochemical reversibility. This observation indicates that further tuning of the optical and electronic properties can be easily achieved. Moreover, the PT-functionalized boranes are well suited as novel electron-deficient building blocks for more complex structures, such as polymers, with interesting and unusual optical and electronic properties. Efforts at polymerizing PT-functionalized boranes to realize even larger conjugated systems are ongoing.

## Supplementary Material

Supplementary information

Crystal structure data
