# Supplementary material for: Pyridalthiadiazole acceptor-functionalized triarylboranes with multi-responsive optoelectronic characteristics
Source: Chem Sci. 2017 Jun 7;8(8):5497–505. doi: 10.1039/c6sc03097a (PMC6103004; doi:10.1039/c6sc03097a)
Supplement: Supplementary file 1 [file SC-008-C6SC03097A-s001.pdf]

Electronic Supplementary Material (ESI) for Chemical Science.
This journal is © The Royal Society of Chemistry 2017

Supporting Information

Pyridalthiadiazole Acceptor-Functionalized Triarylboranes with Multi-Responsive Optoelectronic Characteristics

Xiaodong Yin,[a‡] Kanglei Liu,[a‡] Yi Ren,[a,b] Roger A. Lalancette,[a]

Yueh-Lin Loo,[b,c] and Frieder Jäkle*[a]

[a] Department of Chemistry, Rutgers University - Newark, Newark, NJ 07102, USA.

E-mail: fjaekle@rutgers.edu

[b] Department of Chemical and Biological Engineering, Princeton University, NJ 08544, USA.

[c] Andlinger Center for Energy and the Environment, Princeton University, Princeton, NJ 08544, USA

‡ These authors have contributed equally to this manuscript

Materials and General Methods

All reactions were carried out under an atmosphere of pre-purified nitrogen using either Schlenk techniques or an inert-atmosphere glovebox. Ether solvents were distilled from Na/benzophenone prior to use. Dimethylformamide (DMF) was purified by distillation from DMF pre-dried with 4Å molecular sieve. Hydrocarbon and chlorinated solvents were purified using a solvent purification system (alumina/copper columns for hydrocarbon solvents), and the chlorinated solvents were subsequently distilled from CaH_2 and degassed via several freeze-pump-thaw cycles. All chemicals were purchased from commercial sources and directly used without further purification unless noted otherwise. TBAF•$3H_2O$ and $[Bu_4N][Ph_3SiF_2]$ were purchased from Sigma-Aldrich and the latter was purified by repeated recrystallization from toluene/tetrahydrofuran. Bis(dithiophen-2-yl)(2,4,6-tri-tert-butylphenyl)borane (**BDT**)[1], di(thiophen-2-yl)(2,4,6-tris(trifluoromethyl)phenyl)borane (**FBDT**)[1], (((2,4,6-tri-tert-butylphenyl)boranediyl)bis-(thiophene-2,5-diyl))bis(trimethylstannane) (**BDT-2Sn**)[2], (((2,4,6-tris(trifluoromethyl)phenyl)boranediyl)-bis(thiophene-2,5-diyl))bis(trimethylstannane) (**FBDT-2Sn**)[2] were synthesized according to literature procedures.

NMR data were acquired at ambient temperature unless noted otherwise. 500.0 MHz ^{1}H, 125.7 MHz ^{13}C, 160.4 MHz ^{11}B, and 470.4 MHz ^{19}F NMR spectra were recorded on a 500 MHz Varian INOVA spectrometer or a 500 MHz Bruker AVANCE spectrometer; 599.7 MHz ^{1}H, 150.8 MHz ^{13}C, 192.4 MHz ^{11}B, and 564.3 MHz ^{19}F NMR spectra were recorded on a Varian INOVA 600 spectrometer, which was equipped with a boron-free 5 mm dual broadband gradient probe (Nalorac, Varian Inc., Martinez, CA). ^{11}B NMR spectra were acquired with boron-free quartz NMR tubes. ^{1}H and ^{13}C NMR spectra were referenced internally to solvent signals ($CDCl_3$: 7.26 ppm for ^{1}H NMR, 77.16 ppm for ^{13}C NMR; d8-THF: 3.58 ppm for ^{1}H NMR, 67.57 ppm for ^{13}C NMR) and all other NMR spectra externally to $SiMe_4$ (0 ppm). The following abbreviations are used for signal assignments: Th = thienyl, Mes* = 2,4,6-tri-tert-butylphenyl, ^{F}Mes = 2,4,6-tris(trifluoromethyl)phenyl, PT = pyridalthiadiazolyl, *t*-Bu = *tert*-butyl, s = singlet, d = doublet, t = triplet, q = quartet, m = multiplet, br = broad. NMR titrations were carried out in J-Young NMR tubes with a Teflon screw top using d8-THF that was distilled from Na. Aliquots of a TBAF•$3H_2O$ or $[Bu_4N][Ph_3SiF_2]$ solution in d8-THF were added to the sample solution in a glove box environment under N_2 atmosphere.

EPR measurements at X-band (9.75 GHz) were carried out at room temperature using a Bruker EMX spectrometer. All samples were prepared in a glove box and sealed with a septum.

MALDI-MS and ESI-MS measurements were performed on an Apex-ultra 7T Hybrid FT-MS (Bruker Daltonics). MALDI-TOF MS measurements were performed on a Bruker Ultraflextreme in reflectron mode with delayed extraction. Anthracene (10 mg/mL) was used as the matrix for the MALDI-MS analyses and mixed with the samples (10 mg/mL in chloroform) in a 10:1 ratio, and then spotted on the wells of a target plate. Red phosphorus or sodium iodide clusters were used for calibration.

UV-visible absorption data were acquired on a Varian Cary 5000 UV-Vis/NIR spectrophotometer. The fluorescence data and lifetimes were measured using a Horiba Fluorolog-3 spectrofluorometer equipped with a 388 nm nanoLED and a FluoroHub R-928 detector. Absolute quantum yields (Φ_F) were measured on the HORIBA Fluorolog-3 using a pre-calibrated Quanta-φ integrating sphere. Light from the sample compartment is directed into the sphere via a fiber-optic cable and an F-3000 Fiber-Optic Adapter, and

then returned to the sample compartment (and to the emission monochromator) via a second fiber-optic cable and an F-3000 Fiber-Optic Adapter. UV-vis titrations were conducted by addition in a glove box of aliquots of a TBAF•$3H_2O$ or $[Bu_4N][Ph_3SiF_2]$ solution in distilled THF via microsyringe to a cuvette with a septum closure containing the sample solution in THF (3 mL) and monitored by UV-visible spectroscopy. Data fitting was performed using the commercial HypSpec software package.

Cyclic voltammetry (CV) and square wave voltammetry (SWV) experiments were carried out on a CV-50W analyzer from BASi. The three-electrode system consisted of an Au disk as working electrode, a Pt wire as counter electrode and an Ag wire as the reference electrode. The voltammograms were recorded with ca. 10^{-3} to 10^{-4} M solutions in THF (reduction scans) or DCM (oxidation scans) containing $Bu_4N[PF_6]$ (0.1 M) as the supporting electrolyte. The scans were referenced after the addition of a small amount of ferrocene or decamethylferrocene as internal standard. The potentials are reported relative to the ferrocene/ferrocenium couple. When decamethylferrocene (Fc*) was used as the internal reference the data was converted to $E(Fc^{0/+}) = 0$ V using the equation $E(Fc^{*0/+}) = E(Fc^{0/+}) – 0.50$ V).

DFT calculations were performed with the Gaussian09 suite of programs.[3] The input files were generated from single crystal structures when available or otherwise generated in Chem3D and then pre-optimized in Spartan '12 V 1.2.0. Geometries were then optimized in Gaussian09 using the hybrid density functional B3LYP with a 6-31+G* basis set. Frequency calculations were performed to confirm the presence of local minima (only positive frequencies). The orbital energy levels were calculated by single point calculations using the B3PW91 functional with a 6-311+G* basis set. Vertical excitations were then calculated using TD-DFT methods (wb97xd/6-311+G** or wb97xd/aug-cc-pvdz basis set). For reduced species, the structures and orbital energies were optimized using the uB3LYP/6-31+G* basis set. Orbital representations were plotted with Gaussview 5.08.

Chemical reductions of **BDT-2PT** and **BDT-PT** were conducted in dried and degassed THF with decamethylcobaltocene ($CoCp^*_2$) as reductant (the redox potential for the $[CoCp^*_2]^{+/0}$ couple is –1.94 V vs. $Fc^{+/0}$). For each substrate, an excess amount of $CoCp^*_2$ (>2 eqv.) was added under inert atmosphere, then the UV-Vis spectra were recorded using a sealed quartz cell.

Mobility Measurements: The electron-only devices use ITO and aluminum as electrodes and films of **BDT-2PTTh** with a layer thickness of 125 nm to 213 nm that were spin-coated from $CHCl_3$ solution (10 mg/mL) The electron mobilities were extracted from space charge limited current (SCLC) measurements.

Single crystals of **BDT-2PT** were obtained from a mixture of THF and hexanes. X-ray diffraction intensities were collected on a Bruker SMART APEX II CCD Diffractometer using CuKα (1.54178 Å) radiation at 100(2) K. The structures were refined by full-matrix least squares based on F_2 with all reflections (SHELXTL V5.10; G. Sheldrick, Siemens XRD, Madison, WI). Non-hydrogen atoms were refined with anisotropic displacement coefficients, and hydrogen atoms were treated as idealized contribution. SADABS (Sheldrick, 12 G.M. SADABS (2.01), Bruker/Siemens Area Detector Absorption Correction Program; Bruker AXS: Madison, WI, 1998) absorption correction was applied. Crystallographic data for the structure of **BDT-2PT** have been deposited with the Cambridge Crystallographic Data Center as supplementary publications CCDC 1493046. Copies of the data can be obtained free of charge on application to CCDC, 12 Union Road, Cambridge CB2 1EZ, UK (fax: (+44) 1223-336-033; email: deposit@ccdc.cam.ac.uk).

Synthetic Procedures and Characterization Data

Synthesis of 4,4'-(5,5'-((2,4,6-tri-tert-butylphenyl)boranediyl)bis(thiophene-2,5-diyl))bis(7-bromo-[1,2,5]thiadiazolo[3,4-c]pyridine) (BDT-2PT)

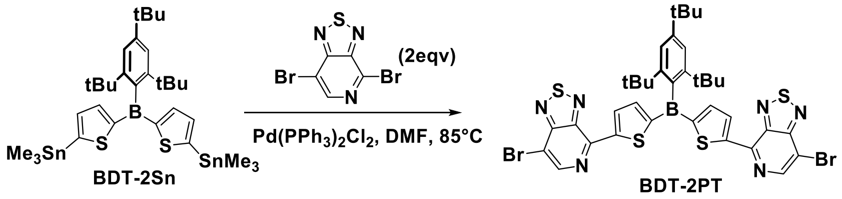

BDT-2Sn (1.49 g, 1.99 mmol) and 4,7-dibromo[1,2,5]thiadiazolo[3,4-c]pyridine (1.42 g, 4.81 mmol) were mixed in a 50 mL Schlenk flask, followed by addition of 10 mL of dry N,N-dimethylformamide (DMF). Dichloro(bis(triphenylphosphine))palladium(II) (0.17 g, 0.24 mmol) was added under nitrogen atmosphere. The reaction mixture was heated to 85 °C and kept stirring at that temperature for 20 h. The mixture was then cooled to room temperature, diluted with 50 mL of dichloromethane, and washed with water (100 mL×2). The organic phase was dried over anhydrous sodium sulfate and the solvent was removed under vacuum at RT. The crude product was redissolved in a small amount of CH_2Cl_2, mixed with silica gel and the solvent removed under vacuum. Column chromatography (silica gel, hexanes:dichloromethane = 5:1) gave **BDT-2PT** as an orange solid (1.15 g, 68%). Single crystals of **BDT-2PT** were obtained from a mixture of THF and hexanes.

^{1}H NMR (500.0 MHz, $CDCl_3$): δ = 8.79 (d, *J* = 3.5 Hz, 2H, Th), 8.75 (s, 2H, PT), 7.93 (br s, 2H, Th), 7.47 (s, 2H, Mes*), 1.42 (s, 9H, *t*-Bu), 1.24 (s, 18H, *t*-Bu); **^{13}C NMR** (125.7 MHz, $CDCl_3$): δ = 156.60, 152.97 (br, C-B), 152.16, 149.75, 149.25, 148.29, 147.43, 145.97, 142.93 (br, C-B), 133.90, 133.68, 123.04, 109.53, 38.82, 35.27, 34.92, 31.61; **^{11}B NMR** (160.4 MHz, $CDCl_3$): δ = 54.4 ($w_{1/2}$ = 3000 Hz). **High-res MALDI-MS (Anthracene)**: *m/z* = 849.0392 (calcd for $C_{36}H_{36}BBr_2N_6S_4$ (MH^+) 849.0344). Elemental analysis calculated for THF solvate $C_{36}H_{35}BBr_2N_6S_4$ • 2 C_4H_8O: C 53.12, H 5.17, N 8.45%; found C 52.84, H 4.82, N 8.13%; the presence of 2 molecules of THF for each molecule of **BDT-2PT** was confirmed by ^{1}H NMR and single crystal X-ray diffraction analysis.

Synthesis of 7-bromo-4-(5-(thiophen-2-yl(2,4,6-tri-tert-butylphenyl)boranyl)thiophen-2-yl)-[1,2,5]thiadiazolo[3,4-c]pyridine (BDT-PT)

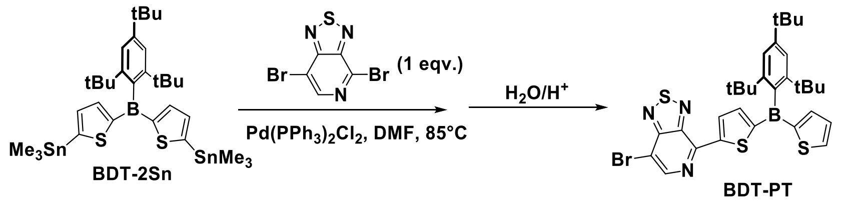

BDT-2Sn (1.20 g, 1.60 mmol) and 4,7-dibromo[1,2,5]thiadiazolo[3,4-c]pyridine (0.47 g, 1.59 mmol) were mixed in a 50 mL Schlenk flask, followed by addition of 10 mL of dry DMF. Dichloro(bis(triphenylphosphine))palladium(II) (60 mg, 0.085 mmol) was added under nitrogen

atmosphere. The reaction mixture was heated to 85 °C and kept stirring at that temperature for 20 h. The mixture was then cooled to room temperature, quenched by adding 10 mL of 10% NH_4Cl aqueous solution and then diluted with 50 mL of dichloromethane. The organic phase was washed with water (100 mL×2) and dried over anhydrous sodium sulfate, and the solvent was removed under vacuum at RT. The crude product was redissolved in a small amount of CH_2Cl_2, mixed with silica gel and the solvent removed under vacuum. Column chromatography (silica gel, hexanes:dichloromethane = 5:1) gave **BDT-PT** as an orange solid (0.70 g, 69%).

^{1}**H NMR** (599.7 MHz, $CDCl_3$): δ = 8.74 (d, *J* = 4.2 Hz, 1H, Th), 8.73 (s, 1H, PT), 7.88 (d, *J* = 4.8 Hz, 1H, Th), 7.83 (br s, 2H, Th), 7.46 (s, 2H, Mes*), 7.24 (t, *J* = 4.2 Hz, 1H, Th), 1.41 (s, 9H, *t*-Bu), 1.20 (s, 18H, *t*-Bu); ^{13}**C NMR** (150.8 MHz, $CDCl_3$): δ = 156.56, 153.53 (br, C-B), 151.98, 149.15, 148.95, 148.30, 147.77, 147.56, 146.06, 142.52 (br, C-B), 141.88 (br, C-B), 136.34, 134.56, 133.52, 128.83, 122.88, 109.28, 38.78, 35.13, 34.88, 31.59; ^{11}**B NMR** (160.3 MHz, $CDCl_3$): δ = 54.1 ($w_{1/2}$ = 2000 Hz). **High-res MALDI-MS** (Anthracene): *m/z* = 636.1333 (calcd for $C_{31}H_{36}BBrN_3S_3$ (MH^+) 636.1348). Elemental analysis calculated for $C_{31}H_{35}BBrN_3S_3$: C 58.49, H 5.54, N 6.60%; found C 58.26, H 5.76, N 6.03%.

Synthesis of 4,4'-(5,5'-((2,4,6-tris(trifluoromethyl)phenyl)boranediyl)bis(thiophene-5,2-diyl))bis(7-bromo-[1,2,5]thiadiazolo[3,4-c]pyridine) (FBDT-2PT)

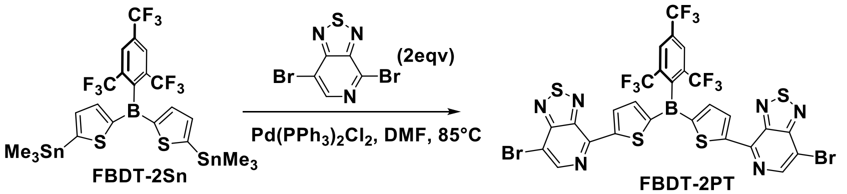

FBDT-2Sn (1.60 g, 2.04 mmol) and 4,7-dibromo-[1,2,5]thiadiazolo[3,4-c]pyridine (1.47 g, 4.98 mmol) were mixed in a 50 mL Schlenk flask, followed by addition of 10 mL of dry DMF. Dichloro(bis(triphenylphosphine))palladium(II) (0.17 g, 0.24 mmol) was added under nitrogen atmosphere. The reaction mixture was heated to 85 °C and kept stirring at that temperature for 20 h. The mixture was then cooled to room temperature, diluted with 50 mL of dichloromethane, and washed with water (100 mL×2). The organic phase was dried over anhydrous sodium sulfate and the solvent was removed under vacuum at RT. The crude product was redissolved in a small amount of CH_2Cl_2, mixed with silica gel and the solvent removed under vacuum. Column chromatography (silica gel, hexanes:dichloromethane = 5:1) gave **FBDT-2PT** as an orange solid (0.99 g, 55 %).

^{1}**H NMR** (599.7 MHz, d8-THF): δ = 8.86 (d, *J* = 4.2 Hz, 2H, Th), 8.76 (s, 2H, PT), 8.51 (s, 2H, ^{F}Mes), 7.89 (d, *J* = 3.6 Hz, 2H, Th); ^{13}**C NMR** (150.8 MHz, d8-THF, 60 °C): δ = 157.61, 153.99, 149.31, 147.58, 146.8 (br), 146.67, 144.83, 145.5 (br), 135.58 (q, J_{C-F} = 31.7 Hz), 133.99, 133.40 (q, J_{C-F} = 35.7 Hz), 127.71, 111.32, $\underline{C}F_3$ not observed; ^{11}**B NMR** (192.4 MHz, d8-THF): δ = 56.1 ($w_{1/2}$ = 3000 Hz); ^{19}**F NMR** (470.7 MHz, $CDCl_3$): δ = -55.95, -63.07. **High-res MALDI-MS** (Anthracene): *m/z* = 884.8068 (calcd for $C_{27}H_9BBr_2F_9N_6S_4$ (MH^+) 884.8088). Elemental analysis calculated for $C_{27}H_8BBr_2F_9N_6S_4$: C 36.59, H 0.91, N 9.48%; found C 38.02, H 1.22, N 8.77%; the slightly high C and H values are likely due to traces of solvents that were difficult to remove even under high vacuum.

Synthesis of 7-bromo-4-(5-(thiophen-2-yl(2,4,6-tris(trifluoromethyl)phenyl)boryl)thiophen-2-yl)-[1,2,5]thiadiazolo[3,4-c]pyridine (FBDT-PT)

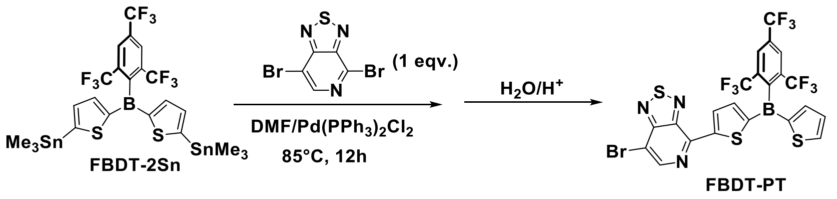

FBDT-2Sn (1.20 g, 1.53 mmol) and 4,7-dibromo[1,2,5]thiadiazolo[3,4-c]pyridine (0.45 g, 1.53 mmol) were mixed in a 50 mL Schlenk flask, followed by addition of 10 mL of dry DMF. Dichloro(bis(triphenylphosphine))palladium(II) (60 mg, 0.085 mmol) was added under nitrogen atmosphere. The reaction mixture was heated to 85 °C and kept stirring at that temperature for 20 h. The mixture was then cooled to room temperature, quenched by adding 10 mL of 10% NH_4Cl aqueous solution and then diluted with 50 mL of dichloromethane. The organic phase was washed with water (100 mL×2) and dried over anhydrous sodium sulfate, and the solvent was removed under vacuum at RT. The crude product was redissolved in a small amount of CH_2Cl_2, mixed with silica gel and the solvent removed under vacuum. Column chromatography (silica gel, hexanes:dichloromethane = 5:1) gave **FBDT-PT** as orange solid (0.65 g, 63%).

^{1}H NMR (599.7 MHz, $CDCl_3$): δ = 8.79 (d, J = 4.2 Hz, 1H, Th), 8.73 (s, 1H, PT), 8.18 (s, 2H, ^{F}Mes), 8.04 (d, J = 4.2 Hz, 1H, Th), 7.74 (d, J = 3.6 Hz, 1H, Th) 7.69 (d, J = 3.6 Hz, 1H, Th), 7.32 (t, J = 4.2 Hz, 1H, Th); **^{13}C NMR** (150.8 MHz, $CDCl_3$): δ = 156.56, 151.41, 148.15, 146.89, 146.00, 144.36 (br), 143.47, 143.22, 141.40 (br), 139.16, 134.60 (q, $J_{C\text{-}F}$ = 34.7 Hz), 133.47, 132.17 (q, $J_{C\text{-}F}$ = 32.0 Hz), 129.52, 126.29, 123.67 (q, $J_{C\text{-}F}$ = 275 Hz), 122.92 (q, $J_{C\text{-}F}$ = 273 Hz), 110.15; **^{11}B NMR** (192.4 MHz, $CDCl_3$): δ = 51.9 ($w_{1/2}$ = 2000 Hz); **^{19}F NMR** (564.3 MHz, $CDCl_3$): δ = -56.12, -63.12. **High-res MALDI-MS** (Anthracene): *m/z* = 671.9072 (calcd for $C_{22}H_9BBrF_9N_3S_3$ (MH^+) 671.9091). Elemental analysis calculated for $C_{22}H_8BBrF_9N_3S_3$: C 39.31, H 1.20, N 6.25%; found C 39.95, H 1.16, N 6.03%; Elemental analysis calculated for hexanes solvate $C_{22}H_8BBrF_9N_3S_3$ • 0.3 C_6H_{14}: C 40.95, H 1.76, N 6.02%; found C 40.91, H 1.98, N 5.69% (Repeated: Found 40.70, 1.47, 5.87%); the presence of hexanes in the sample was confirmed by ^{1}H NMR analysis.

Synthesis of 1,1'-(((((2,4,6-tri-tert-butylphenyl)boranediyl)bis(thiophene-5,2-diyl)) bis([1,2,5]thiadiazolo[3,4-c]pyridine-4,7-diyl))bis(thiophene-5,2-diyl))bis(hexan-1-one) (BDT-2PTTh)

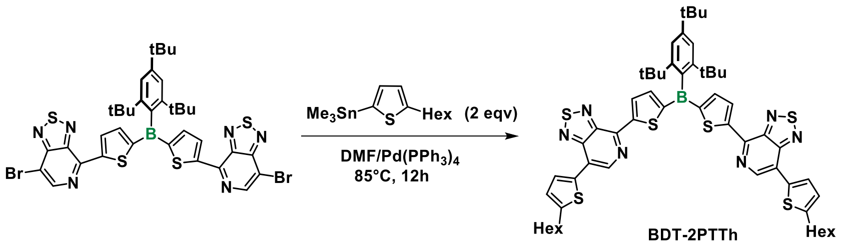

BDT-2PT (0.80 g, 0.94 mmol) and 5-hexyl-2-trimethylstannylthiophene (1.04 g, 3.14 mmol) were mixed in a 50 mL Schlenk flask, followed by addition of 10 mL of dry DMF.

Tetrakis(triphenylphosphine)palladium(0) (115 mg, 0.10 mmol) was added under nitrogen atmosphere. The reaction mixture was heated to 85 °C and kept stirring at that temperature for 20 h. The mixture was then cooled to room temperature, diluted with 50 mL of dichloromethane, and washed with water (100 mL×2). The organic phase was dried over anhydrous sodium sulfate and the solvent was removed under vacuum at RT. The crude product was redissolved in a small amount of CH_2Cl_2, mixed with silica gel and the solvent removed under vacuum. Column chromatography (silica gel, hexanes:dichloromethane = 5:1) gave **BDT-2PTTh** as a red solid (0.83 g, 86%). The sample was recrystallized from a mixture of THF and pentane at –30 °C.

^{1}H NMR (599.7 MHz, $CDCl_3$): δ = 8.86 (overlap of 2H, Th and 2H, PT), 8.01 (d, *J* = 3.6 Hz, 2H, Th-Hex), 7.96 (br s, 2H, Th-B), 7.48 (s, 2H, Mes*), 6.93 (d, *J* = 4.2 Hz, 2H, Th-Hex), 2.91 (t, *J* =7.2 Hz, 4H, α–CH_2), 1.77 (m, 4H, β–CH_2), 1.43 (s, 9H, *t*-Bu), 1.35 (m, 12H, Hex), 1.25 (s, 18H, *t*-Bu), 0.91 (t, *J* = 7.2 Hz, 6H, Me); **^{13}C NMR** (150.8 MHz, $CDCl_3$): δ = 154.96, 152.51 (br, C-B), 152.13, 150.47, 149.48, 149.03, 148.49, 145.47, 142.85 (br, C-B), 140.02, 134.27, 133.92, 132.89, 128.70, 125.71, 122.99, 121.72, 38.85, 35.27, 34.90, 31.72, 31.63, 30.52, 28.99, 22.74, 14.24; **^{11}B NMR** (160.3 MHz, $CDCl_3$): δ = 56.9 ($w_{1/2}$ = 3000 Hz). **High-res MALDI-MS (Anthracene)**: *m/z* = 1024.3660 (calcd for $C_{56}H_{65}BN_6S_6$ (M^+) 1024.3688). Elemental analysis calculated for $C_{56}H_{65}BN_6S_6$: C 65.60, H 6.39, N 8.20%; found C 65.23, H 5.97, N 7.93%. UV-Vis absorption and photoluminescence data: λ_{abs} (THF) = 495 nm; λ_{abs} (solid) = 510, 560 nm; λ_{em} (THF) = 610 nm; λ_{em} (solid) = 695 nm.

NMR Data for Fluoride Anion Complexation of BDT / BDT-PT and FBDT / FBDT-PT in d8-THF

For BDT: ^{1}**H NMR** (500.0 MHz, d8-THF): δ = 7.95 (d, *J* = 4.7 Hz, 2H, Th), 7.71 (br, 2H, Th), 7.48 (s, 2H, Mes*), 7.20 (t, *J* = 4.1 Hz, 2H, Th), 1.38 (s, 9H, *t*-Bu), 1.16 (s, 18H, *t*-Bu); ^{11}**B NMR** (160.4 MHz, d8-THF): δ = 52.5.

For BDT•F^{-} (after addition of large excess of TBAF): δ = 7.07 (2H, Mes*), 6.95 (br, 2H, Th), 6.86 (br, 2H, Th), 6.71 (br, 2H, Th), 1.30 (s, 1.5H, *t*-Bu), 1.13 (s, 18H, *t*-Bu); ^{11}**B NMR** (160.4 MHz, d8-THF): δ = 2.5; ^{19}**F NMR** (470.4 MHz, d8-THF): δ = –130.9 (br, B-F).

For BDT-PT: ^{1}**H NMR** (500.0 MHz, d8-THF): δ = 8.77 (d, *J* = 3.8 Hz, 1H, Th), 8.71 (s, 1H, PT), 8.03 (d, *J* = 4.7 Hz, 1H, Th), 7.85 (br, 1H, Th), 7.81 (br, 1H, Th), 7.52 (s, 2H, Mes*), 7.26 (t, *J* = 4.1 Hz, 1H, Th), 1.41 (s, 9H, *t*-Bu), 1.21 (s, 18H, *t*-Bu).

For BDT-PT•F^{-} (after addition of large excess of TBAF): Signals are strongly broadened, indicative of radical formation. The color of the solution changes to deep blue.

For FBDT: ^{1}**H NMR** (500.0 MHz, d8-THF): δ = 8.41 (s, 2H, FMes), 8.14 (d, *J* = 4.7 Hz, 2H, Th), 7.61 (br, 2H, Th), 7.29 (t, *J* = 3.6 Hz, 2H, Th); ^{11}**B NMR** (160.4 MHz, d8-THF): δ = 51.2; ^{19}**F NMR** (470.4 MHz, d8-THF): δ = -56.65, -63.79.

For FBDT•F^{-} (after addition of [Bu$_4$N][Ph$_3$SiF$_2$]): ^{1}**H NMR** (500.0 MHz, d8-THF): δ = 7.94 (s, 2H, FMes), 7.08 (d, *J* = 4.5 Hz, 2H, Th), 6.81 (br, 2H, Th), 6.78 (br, 2H, Th); ^{11}**B NMR** (160.4 MHz, d8-THF): δ = 1.8; ^{19}**F NMR** (470.4 MHz, d8-THF): δ = -53.65, -53.69, -63.51, -151.1 (br, B-F).

For FBDT-PT: ^{1}**H NMR** (500.0 MHz, d8-THF): δ = 8.80 (d, *J* = 3.9 Hz, 1H, Th), 8.73 (s, 1H, PT), 8.46 (s, 2H, FMes), 8.23 (d, *J* = 4.6 Hz, 1H, Th), 7.77 (d, *J* = 3.9 Hz, 1H, Th), 7.74 (d, *J* = 3.7 Hz, 1H, Th), 7.35 (t, 1H, *J* = 4.2 Hz, Th); ^{11}**B NMR** (160.4 MHz, d8-THF): δ = 52.9; ^{19}**F NMR** (470.4 MHz, d8-THF): δ = -56.55, -63.77.

For FBDT-PT•F^{-} (after addition of [Bu$_4$N][Ph$_3$SiF$_2$]): ^{1}**H NMR** (500.0 MHz, d8-THF): δ = 8.55 (d, *J* = 3.5 Hz, 1H, Th), 8.46 (s, 1H, PT), 7.99 (s, 2H, FMes), 7.14 (d, *J* = 4.6 Hz, 1H, Th), 6.98 (m, 1H, Th), 6.84 (t, *J* = 4.1 Hz, 1H, Th), 6.81 (m, 1H, Th); ^{11}**B NMR** (160.4 MHz, d8-THF): δ = 1.8; ^{19}**F NMR** (470.4 MHz, d8-THF): δ = -53.73, -53.77, -63.54, -153.6 (br, B-F).

Photophysical Data

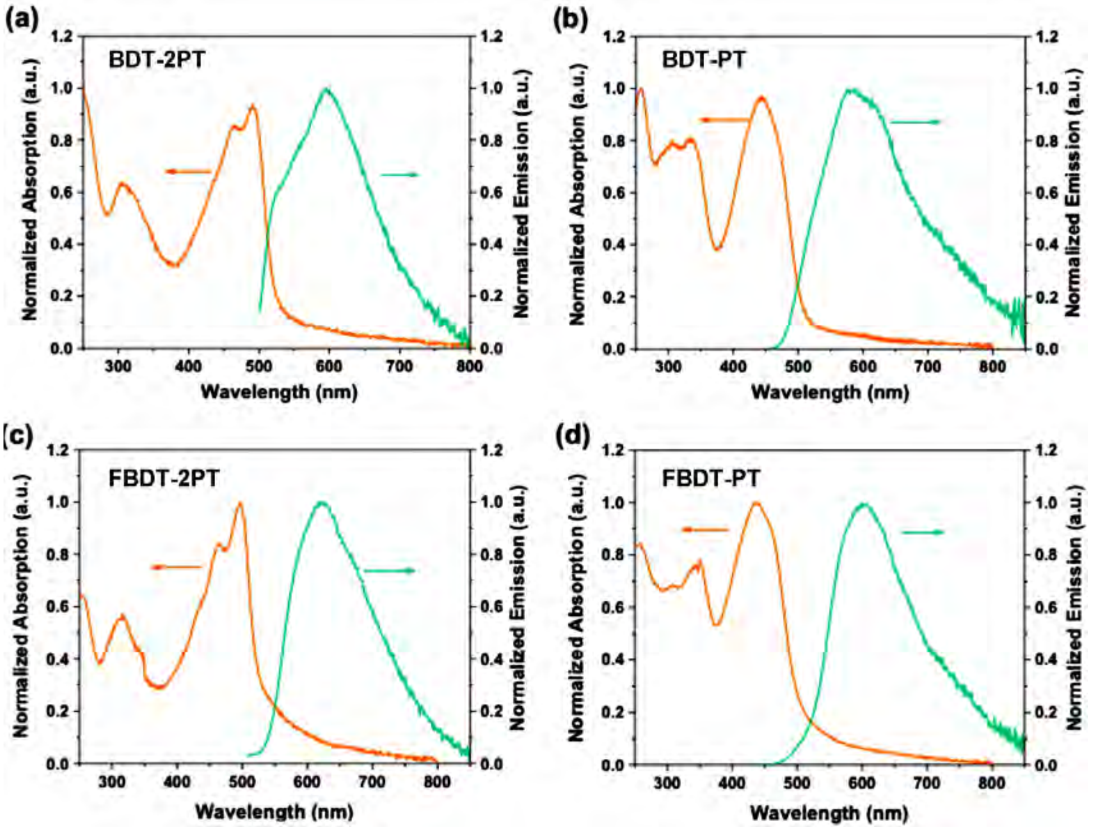

Figure S1. UV-Vis and fluorescence spectra of thin films for (a) **BDT-2PT**, (b) **BDT-PT**, (c) **FBDT-2PT**, (d) **FBDT-PT**.

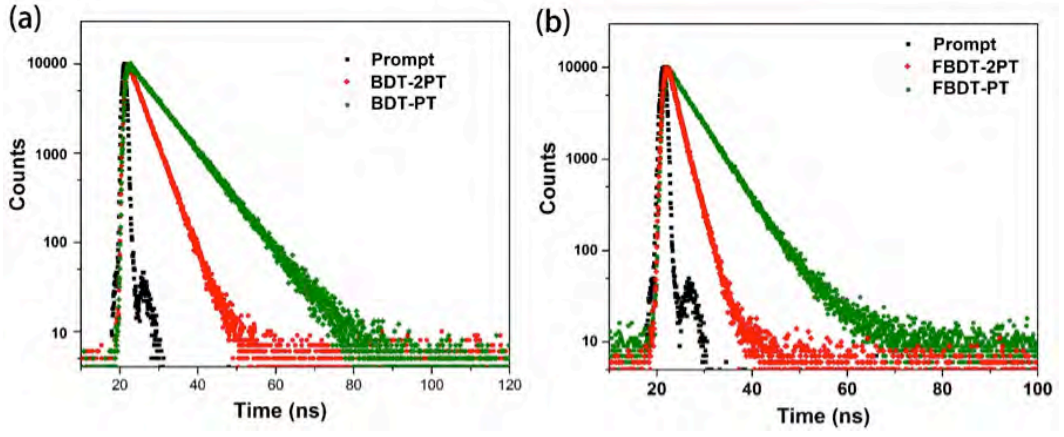

Figure S2. Fluorescence decays of (a) non-fluorinated and (b) fluorinated borane compounds in degassed THF (1×10^{-5} mol L^{-1}) excited with a 388 nm nanoLED.

Electrochemical Data

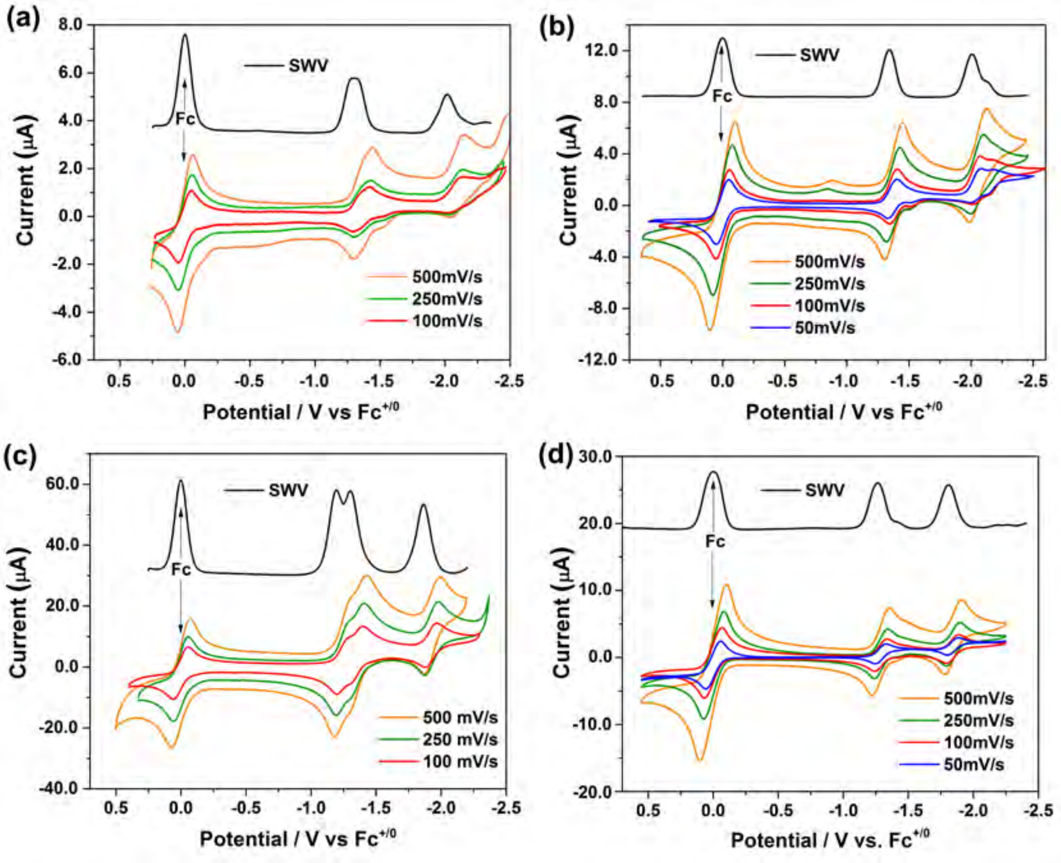

Figure S3. Square wave (100 mV/s) and cyclic voltammetry plots of (a) **BDT-2PT**, (b) **BDT-PT**, (c) **FBDT-2PT**, (d) **FBDT-PT** in THF/0.1M $Bu_4N[PF_6]$ (1×10^{-3} mol L^{-1}; vs. $Fc^{0/+}$).

Table S1. Cyclic voltammetry data of Pyridalthiadiazole-Borane compounds (scan rate: 100 mV/s).

Entry	E_{PT} / V [a]	$\Delta E_p(E_{pc}-E_{pa})$ / mV	E_{boron} / V [a]	$\Delta E_p(E_{pc}-E_{pa})$ / mV
**BDT-2PT**	-1.317	[b]	-2.084	[c]
	-1.391	[b]		
**BDT-PT**	-1.336	60	-2.038	[c]
**FBDT-2PT**	-1.238	~70 [b]	-1.920	80
	-1.353	~80 [b]		
**FBDT-PT**	-1.288	90	-1.839	87

[a] Reported vs. $E_{Fc}^{+/0}$; [b] not determined or only estimated due to overlap of two redox waves; [c] For the boron-centered reduction of **BDT-PT** and **BDT-2PT** a secondary process is evident at low scan rates; this might be due to further reaction of the multiply reduced species at highly negative potentials and is not observed for the more easily reduced fluorinated species.

DFT Calculation Results for Neutral Lewis Acids

Table S2. Frontier orbital representations (scaling radii of 75%, isovalue = 0.02) and energy levels of neutral borane-PT compounds (structure optimization with B3LYP/6-31+G*, single point energy calculations with B3PW91/6-311+G*).

	**BDT-PT**	**BDT-2PT**	**FBDT-PT**	**FBDT-2PT**
**LUMO+2**		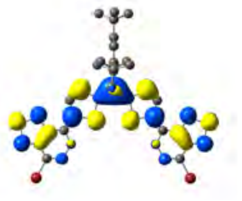 **-2.43 eV**		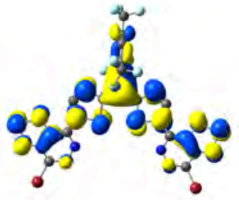 **-2.71 eV**
**LUMO+1**	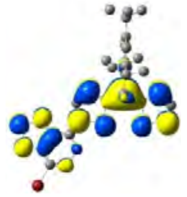 **-2.35 eV**	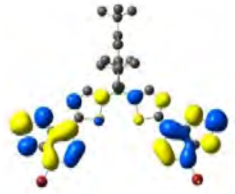 **-3.44 eV**	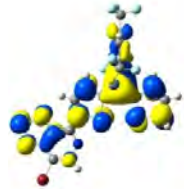 **-2.67 eV**	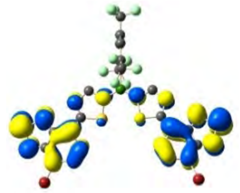 **-3.55 eV**
**LUMO**	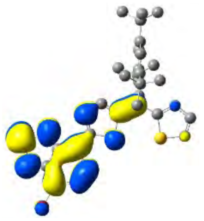 **-3.51 eV**	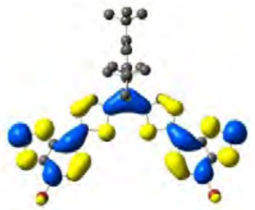 **-3.62 eV**	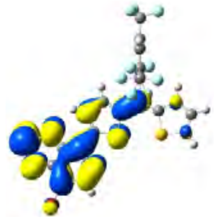 **-3.68 eV**	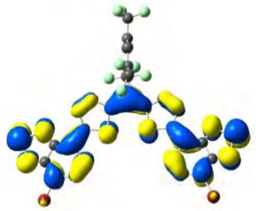 **-3.76 eV**
**HOMO**	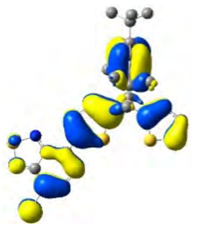 **-6.30 eV**	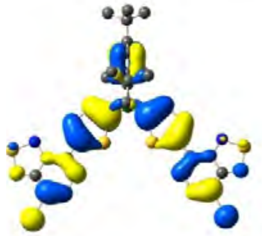 **-6.27 eV**	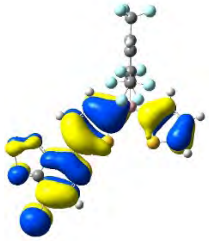 **-6.60 eV**	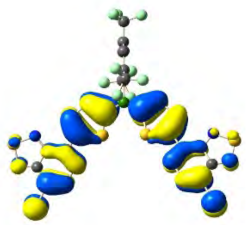 **-6.50 eV**
**HOMO-1**	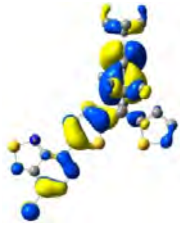 **-6.46 eV**	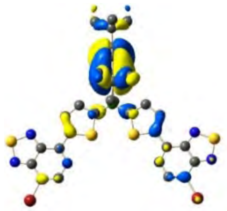 **-6.49 eV**	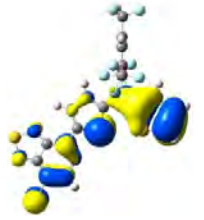 **-7.28 eV**	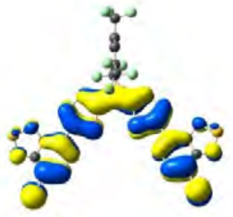 **-6.86 eV**

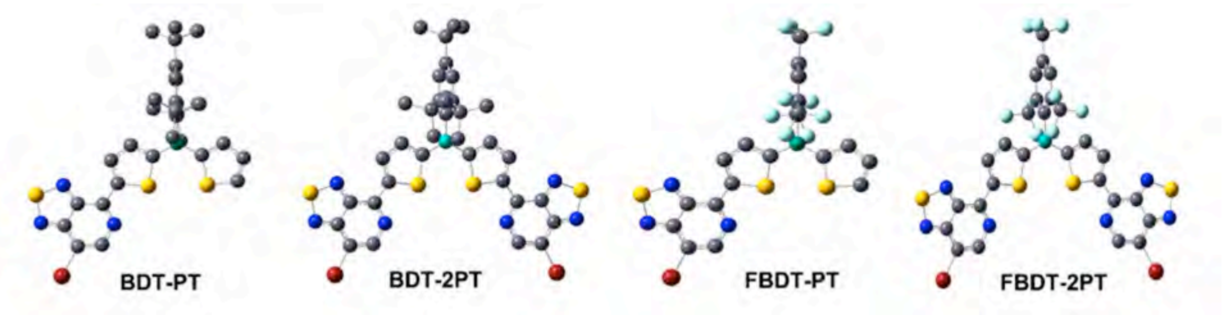

Figure S4. DFT optimized structures of neutral borane-PT compounds (B3LYP/6-31+G*). C grey, B blue-green, Br red, F light blue, N blue, S yellow.

Table S3. TD-DFT calculation results for **BDT-PT** and **FBDT-PT** (wb97xd/6-311+G**).

**BDT-PT**			**FBDT-PT**		
λ (nm)	Oscillator strength *f*	Assignment (Probability)	λ (nm)	Oscillator strength *f*	Assignment (Probability)
**411.74**	**0.6209**	**H→L (0.81)**	**404.46**	**0.6420**	**H→L (0.92)**
311.90	0.0235	H-5→L (0.32)	311.60	0.0822	H-3→L (0.23)
		H-4→L (0.25)			H-1→L (0.28)
		H-3→L (0.17)			H→L+1 (0.23)
308.26	0.0032	H-2→L (0.20)	300.54	0.1557	H-3→L (0.36)
		H-2→L+1 (0.22)			H-2→L (0.13)
		H-1→L (0.26)			H→L+1 (0.34)
		H-1→L+2 (0.20)	271.04	0.0518	H-7→L (0.28)
302.62	0.1369	H-5→L (0.13)			H-4→L (0.15)
		H→L+1 (0.42)			H-2→L+1 (0.11)
285.32	0.0457	H-2→L (0.21)			H-1→L+1 (0.14)
		H-2→L+1 (0.18)	267.76	0.0631	H-7→L (0.10)
		H-1→L (0.22)			H-4→L (0.13)
		H-1→L+1 (0.20)			H-3→L (0.10)
273.09	0.0608	H-9→L (0.16)			H-2→L (0.13)
		H-6→L (0.21)			H-2→L+1 (0.21)
		H-3→L (0.10)	266.93	0.0255	H-7→L (0.11)
		H-3→L+1 (0.33)			H-4→L (0.14)
268.79	0.0659	H-6→L (0.35)			H-2→L (0.10)
		H-3→L (0.16)			H-1→L (0.22)
		H→L+1 (0.29)			H→L+1 (0.19)
265.06	0.1583	H-9→L (0.42)	261.00	0.2708	H-7→L (0.24)
		H-4→L+1 (0.11)			H-4→L (0.18)
					H-1→L+1 (0.26)

Table S4. TD-DFT data of **BDT-2PT** and **FBDT-2PT** (wb97xd/6-311+G**).

**BDT-2PT**		
λ (nm)	Oscillator strength *f*	Assignment (Probability)
**429.51**	**0.7472**	**H→L (0.65)**
**400.75**	**0.4676**	**H-1→L (0.45)**<br>**H→L+1 (0.47)**
320.98	0.0011	H-3→L (0.25)<br>H-3→L+2 (0.14)<br>H-2→L (0.23)<br>H-2→L+2 (0.10)
319.64	0.002	H-9→L+1 (0.28)<br>H-8→L (0.38)
319.38	0.0022	H-9→L (0.38)<br>H-8→L+1 (0.37)
319.06	0.0196	H-4→L (0.37)<br>H→L+2 (0.14)
311.31	0.0754	H-4→L (0.21)<br>H→L+2 (0.25)
304.75	0.006	H-5→L (0.54)<br>H-4→L+1 (0.33)
295.88	0.0528	H-3→L (0.23)<br>H-3→L+2 (0.11)<br>H-2→L (0.32)<br>H-2→L+2 (0.15)
290.69	0.1339	H-1→L (0.25)<br>H-1→L+2 (0.35)<br>H→L+1 (0.20)

**FBDT-2PT**		
λ (nm)	Oscillator strength *f*	Assignment (Probability)
**422.99**	**0.8239**	H-1→L+1 (0.24)<br>**H→L (0.71)**
**387.98**	**0.4348**	**H-1→L (0.44)**<br>**H→L+1(0.51)**
319.95	0.0071	H-7→L (0.30)<br>H-6→L+2 (0.28)<br>H-2→L (0.13)
317.53	0.0351	H-7→L (0.14)<br>H-2→L (0.20)<br>H-1→L+1 (0.11)<br>H→L+2 (0.19)
305.37	0.1048	H-4→L (0.10)<br>H-3→L+1 (0.11)<br>H-2→L (0.34)<br>H→L+2 (0.23)
304.75	0.0137	H-3→L (0.62)<br>H-2→L+1 (0.23)
285.31	0.1215	H-1→L (0.30)<br>H-1→L+2 (0.30)<br>H→L+1 (0.22)
276.43	0.0029	H-5→L (0.24)<br>H-4→L+1 (0.31)

Table S5. Comparison of TD-DFT calculation results for **FBDT-PT** using wb97xd/aug-cc-pvdz versus wb97xd/6-311+G** as basis set.

Calculations were performed on one selected compound using the larger basis set to validate our computational approach. We find that the lowest energy transition is predicted at essentially the same energy and with the same orbital contributions using the wb97xd/aug-cc-pvdz basis set as in the case of corresponding calculations with the wb97xd/6-311+G** basis set.

**FBDT-PT** (wb97xd/6-311+G**)			**FBDT-PT** (wb97xd/aug-cc-pvdz)		
λ (nm)	Oscillator strength *f*	Assignment (Probability)	λ (nm)	Oscillator strength *f*	Assignment (Probability)
**404.46**	**0.6420**	**H→L (0.92)**	**404.65**	**0.6277**	**H→L (0.92)**
311.60	0.0822	H-3→L (0.23)<br>H-1→L (0.28)<br>H→L+1 (0.23)	311.53	0.1020	H-3→L (0.20)<br>H-1→L (0.27)<br>H→L+1 (0.27)
300.54	0.1557	H-3→L (0.36)<br>H-2→L (0.13)<br>H→L+1 (0.34)	300.41	0.1372	H-3→L (0.41)<br>H-2→L (0.12)<br>H→L+1 (0.31)
271.04	0.0518	H-7→L (0.28)<br>H-4→L (0.15)<br>H-2→L+1 (0.11)<br>H-1→L+1 (0.14)	272.45	0.0491	H-7→L (0.44)<br>H-4→L (0.11)<br>H-1→L+1 (0.13)
267.76	0.0631	H-7→L (0.10)<br>H-4→L (0.13)<br>H-3→L (0.10)<br>H-2→L (0.13)<br>H-2→L+1 (0.21)	268.05	0.0519	H-7→L (0.12)<br>H-3→L (0.11)<br>H-3→L+1 (0.09)<br>H-2→L (0.22)<br>H-2→L+1 (0.34)
266.93	0.0255	H-7→L (0.11)<br>H-4→L (0.14)<br>H-2→L (0.10)<br>H-1→L (0.22)<br>H→L+1 (0.19)	266.58	0.0454	H-4→L (0.28)<br>H-1→L (0.25)<br>H→L+1 (0.26)
261.00	0.2708	H-7→L (0.24)<br>H-4→L (0.18)<br>H-1→L+1 (0.26)	261.00	0.2708	H-7→L (0.24)<br>H-4→L (0.18)<br>H-1→L+1 (0.26)
			261.83	0.2660	H-7→L (0.19)<br>H-4→L (0.20)<br>H-1→L+1 (0.29)

Spectral Data for Cp^*_2Co Reduction in THF

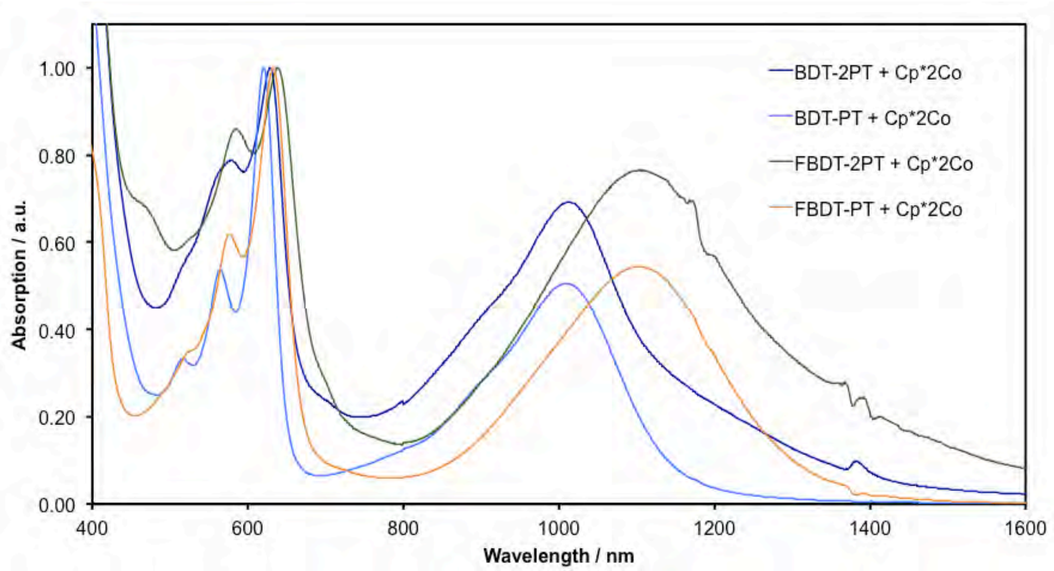

Figure S5a. UV-Vis spectra of (a) **BDT-2PT**, (b) **BDT-PT**, (c) **FBDT-2PT**, and (d) **FBDT-PT** after chemical reduction with an excess of Cp^*_2Co in THF solution.

(a) BDT-2PT: λ_{max} = 579, 628, 1013 nm **(b) BDT-PT**: λ_{max} = 564, 620, 1010 nm

(c) FBDT-2PT: λ_{max} = 584, 638, 1105 nm **(d) FBDT-PT**: λ_{max} = 576, 633, 1103 nm

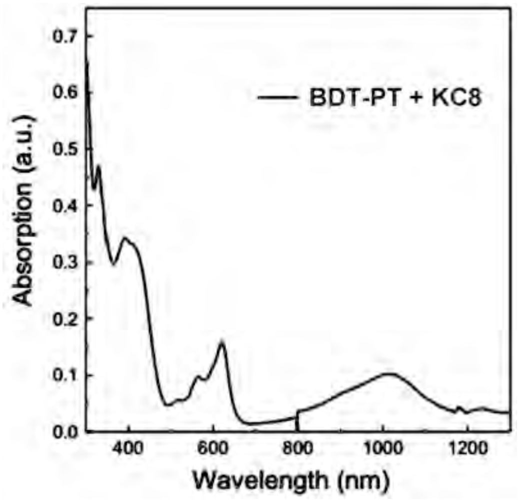

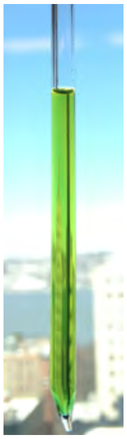

Figure S5b. (Left) UV-Vis spectrum of a solution of **BDT-PT** in THF after addition of a small amount of KC8; (Right) Photograph of a solution of **BDT-PT** in THF after addition of a small amount of KC8. The absorption spectrum is identical to that observed after reduction with $CoCp_2^*$, indicating formation of the same radical anion species.

DFT Calculation Results for Radical Anions and Dianions

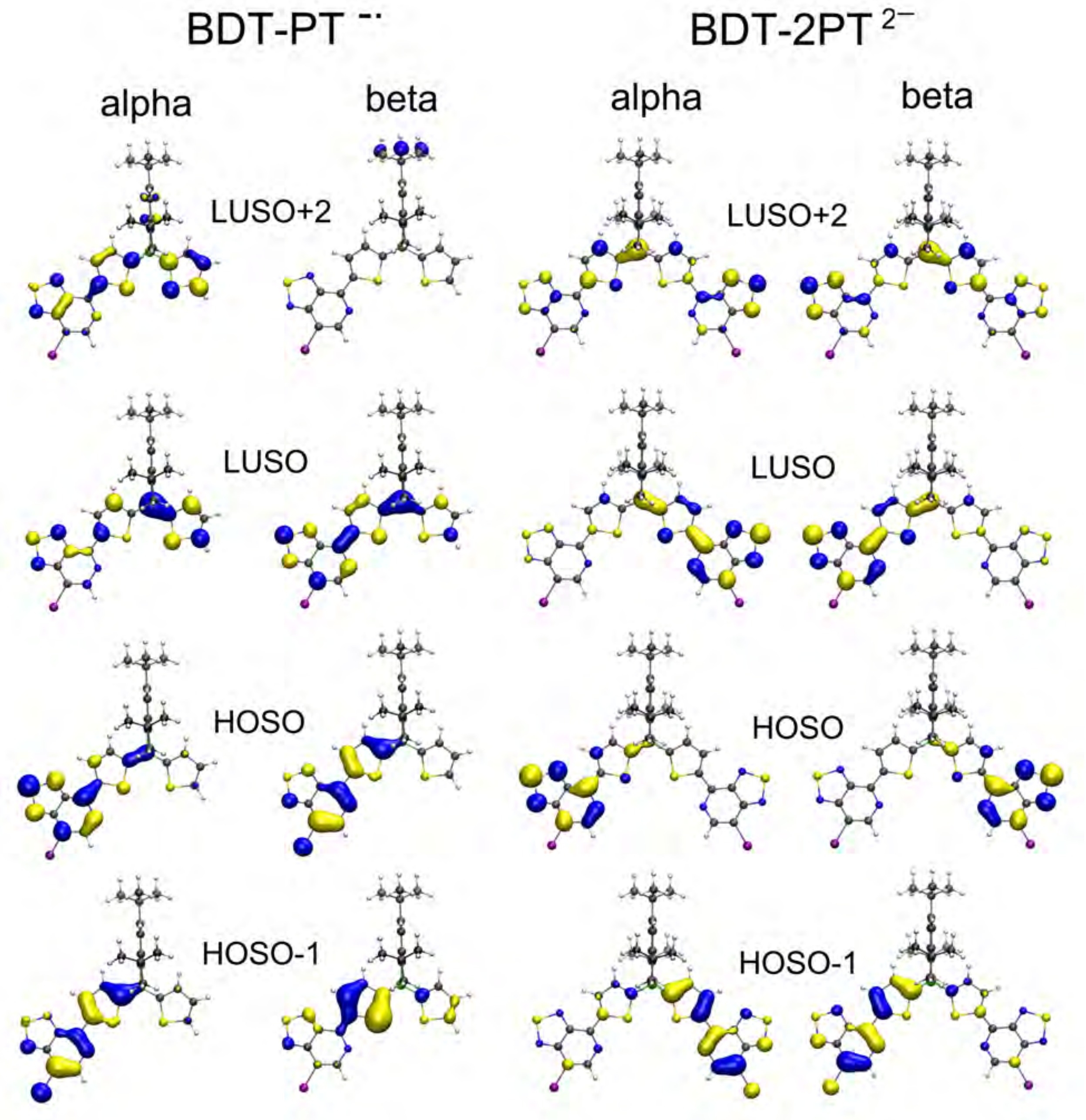

Figure S6. Plots of selected calculated orbitals of (left) **BDT-PT**$^{-·}$ and (right) **BDT-2PT**$^{2-}$ (uB3LYP/6-31+G*, isovalue = 0.005). C grey, B green, Br red, N blue, S yellow.

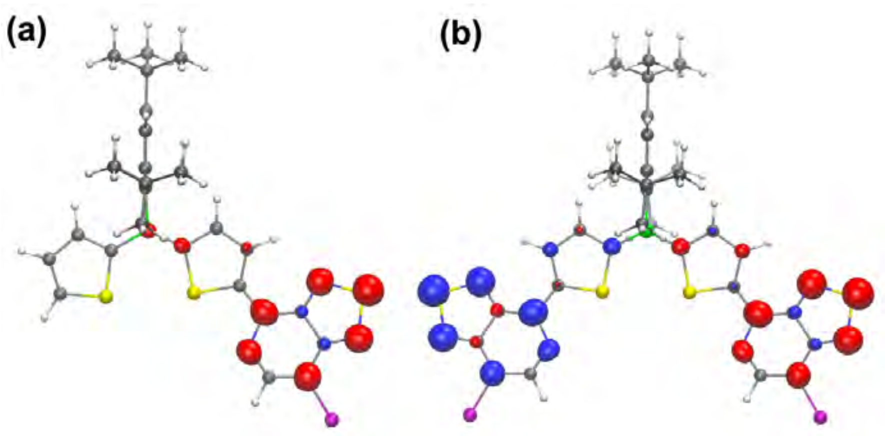

Figure S7. Illustration of calculated spin density of (a) **BDT-PT**$^{-\cdot}$ and (b) **BDT-2PT**$^{2-}$ (uB3LYP/6-31G**, isovalue = 0.005). C grey, B green, Br red, N blue, S yellow.

Table S6. TD-DFT data of **BDT-PT**$^{-\cdot}$ and **BDT-2PT**$^{2-}$ (uB3LYP/6-311+G**).

**BDT-PT**$^{-\cdot}$			**BDT-2PT**$^{2-}$		
λ (nm)	Oscillator strength *f*	Assignment (Probability) [a]	λ (nm)	Oscillator strength *f*	Assignment (Probability) [a]
**1095.93**	**0.1989**	**αH→αL (0.95)**	1213.78	0.0051	αH→αL (0.49)
		**βH-1→βH (0.05)**			βH→βL (0.49)
**600.98**	**0.2098**	**αH→αL (0.04)**	**1097.76**	**0.3567**	**αH→αL (0.48)**
		**αH→αL+2 (0.27)**			**βH →βL (0.48)**
		**βH-1→βH (0.59)**	792.28	0.0545	αH→αL+1 (0.42)
**567.36**	**0.1736**	**αH→αL+2 (0.61)**			βH→βL+1 (0.42)
		**βH-1→βH (0.27)**	726.80	0.0125	αH-1→αL (0.05)
502.59	0.0033	αH→αL+3 (0.69)			αH→αL+1 (0.39)
		αH→αL+5 (0.09)			βH-1→βL (0.05)
497.76	0.0161	αH-1→αL (0.41)			βH→βL+1 (0.39)
		αH→αL+3 (0.14)	**583.11**	**0.3525**	**αH-1→αL (0.41)**
		αH→αL+18 (0.05)			**βH-1→βL (0.41)**
		βH-3→βH (0.03)	**562.47**	**0.1602**	**αH-1→αL (0.37)**
		βH-1→βL (0.24)			**αH→αL+1 (0.06)**
					**βH-1→βL (0.37)**
					**βH→βL+1 (0.06)**

[a] α-HOSO=αH, αLUSO=αL; β-HOSO=βH, β-LUSO=βL

Anion Binding Data for BDT

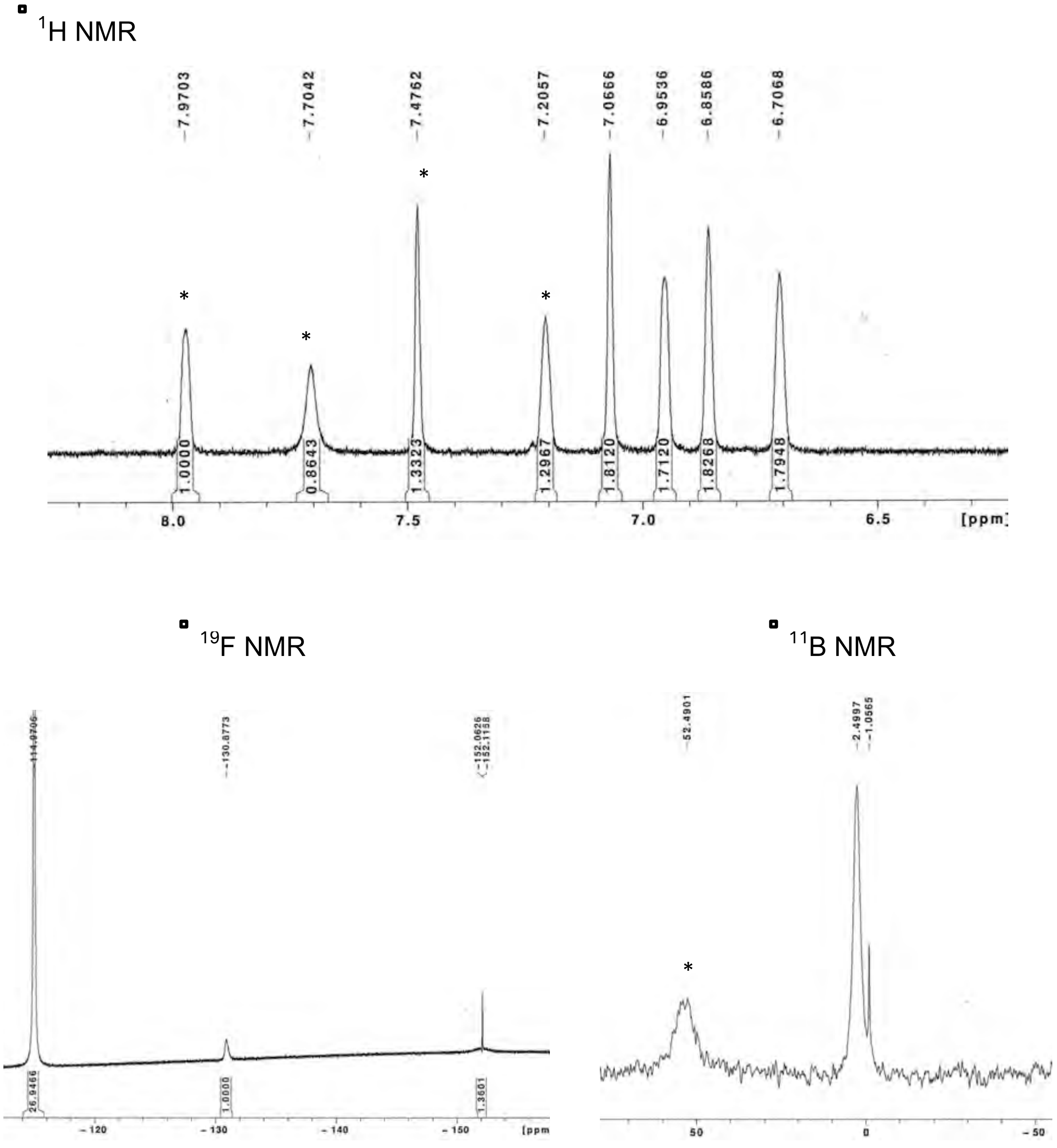

Figure S8. ^{1}H (aromatic region), ^{11}B, and ^{19}F NMR data after treatment of **BDT** (2.0×10^{-2} mmol in 0.55 mL d8-THF) with 20 equivalents of TBAF•$3H_2O$ (2.0×10^{-1} M in d8-THF). Residual uncomplexed **BDT** is indicated with an asterisk.

The signal at ca. +52.5 ppm in the ^{11}B NMR is assigned to the free borane, the signal at +2 ppm to the fluoride anion complex **BDT•F**$^{-}$. The broad signal at ca. –130.9 ppm in the ^{19}F NMR is assigned to the anion complex **BDT•F**$^{-}$, that at ca. –115.0 ppm to unbound fluoride anions (excess), and the signal at –152 ppm to a trace of HF_2^{-} anion.

Additional Anion Binding Data

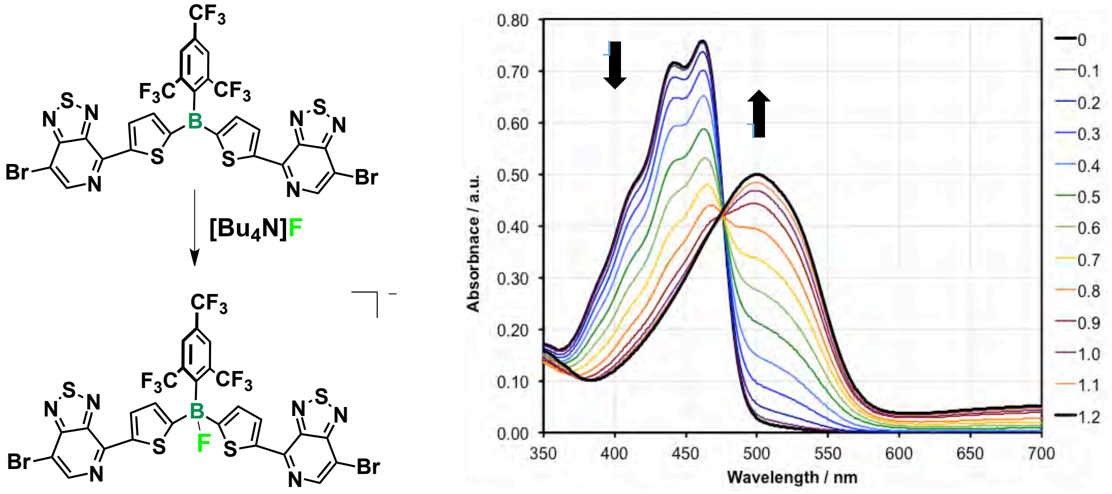

Figure S9. Plot illustrating the UV-Vis titration of a solution of **FBDT-2PT** (3.8×10^{-5} mmol in 3 mL of THF) with TBAF•$3H_2O$ in THF (1.27×10^{-3} M). The titration was carried out under nitrogen atmosphere using a septum-sealed cuvette to which aliquots of the anion solutions were added successively.

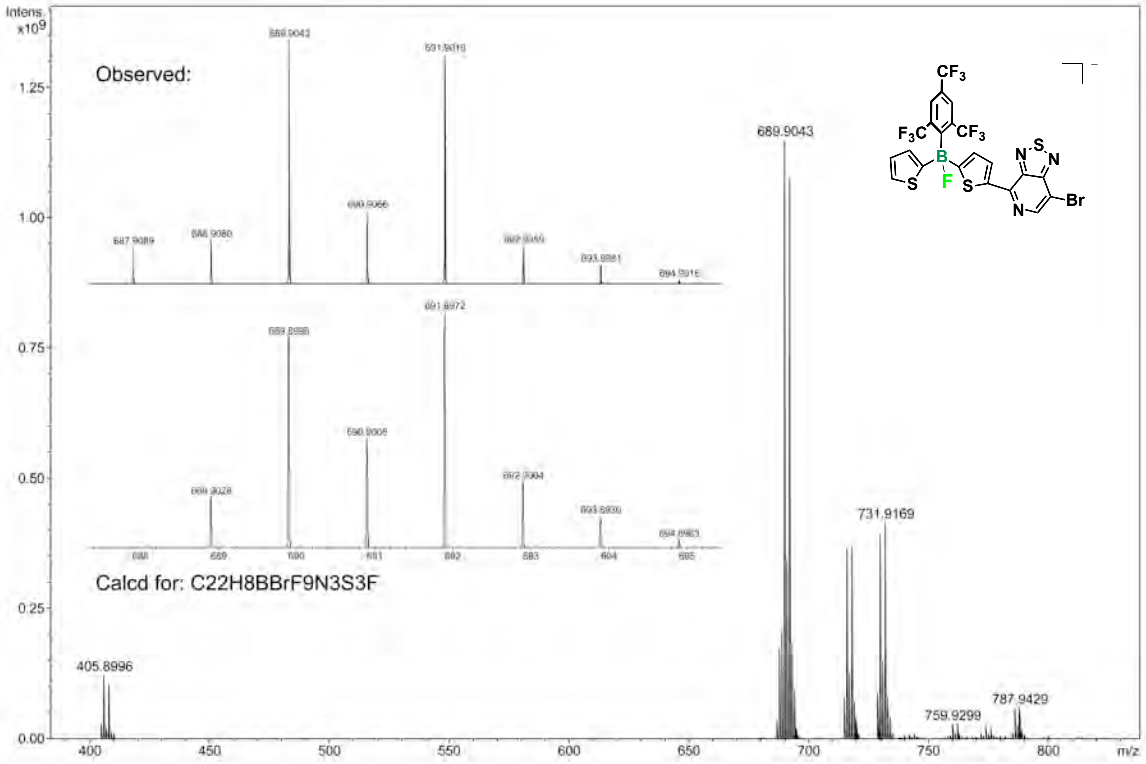

Figure S10. ESI data acquired in negative mode for a solution of **FBDT-PT** in THF to which an excess of TBAF•$3H_2O$ was added.

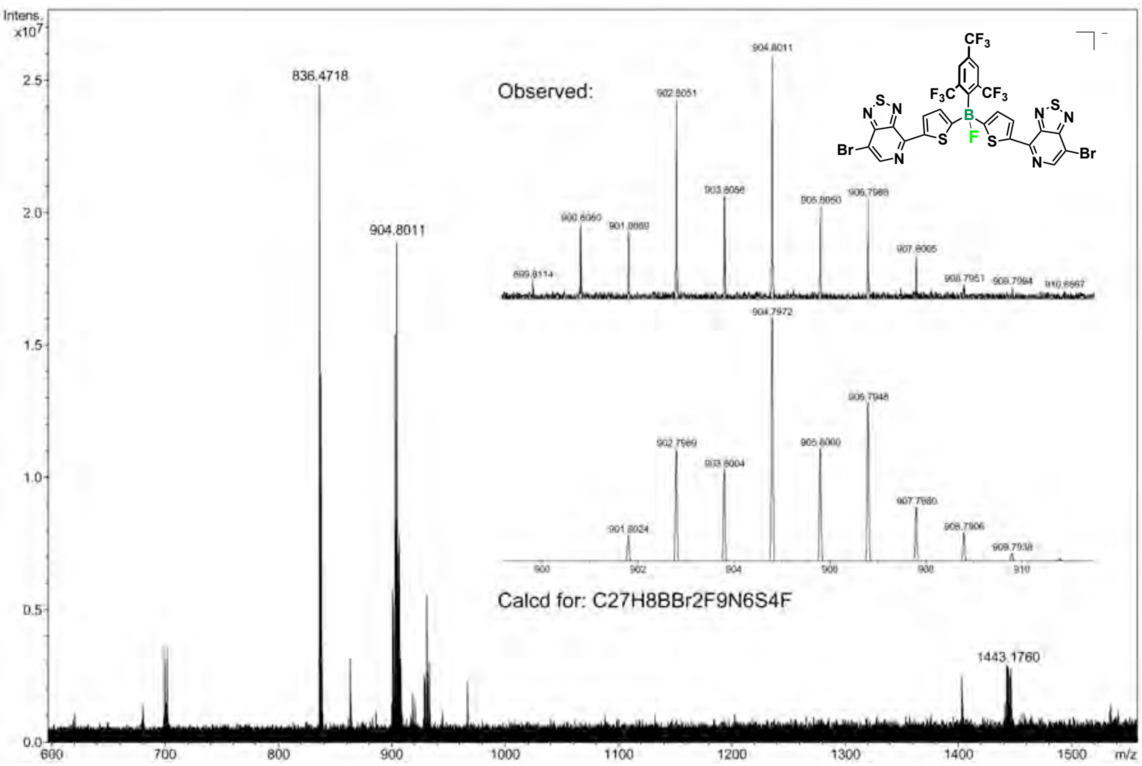

Figure S11. ESI data a cquired in negative mode for a solution of **FBDT-2PT** in THF to which an excess of TBAF•3H_2O was added.

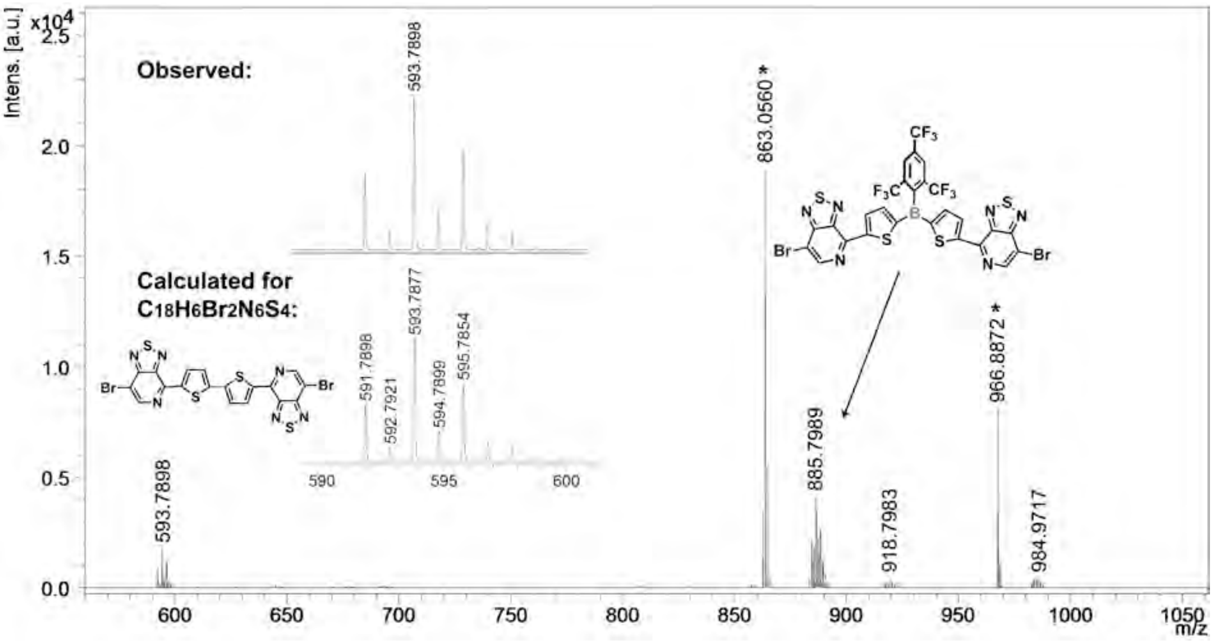

Figure S12. MALDI-TOF MS data for **FBDT-2PT** (negative mode) after addition of TBAF•3H_2O and attempted recovery of the free acid by addition of excess BF_3•OEt_2. * denotes matrix or impurity peak.

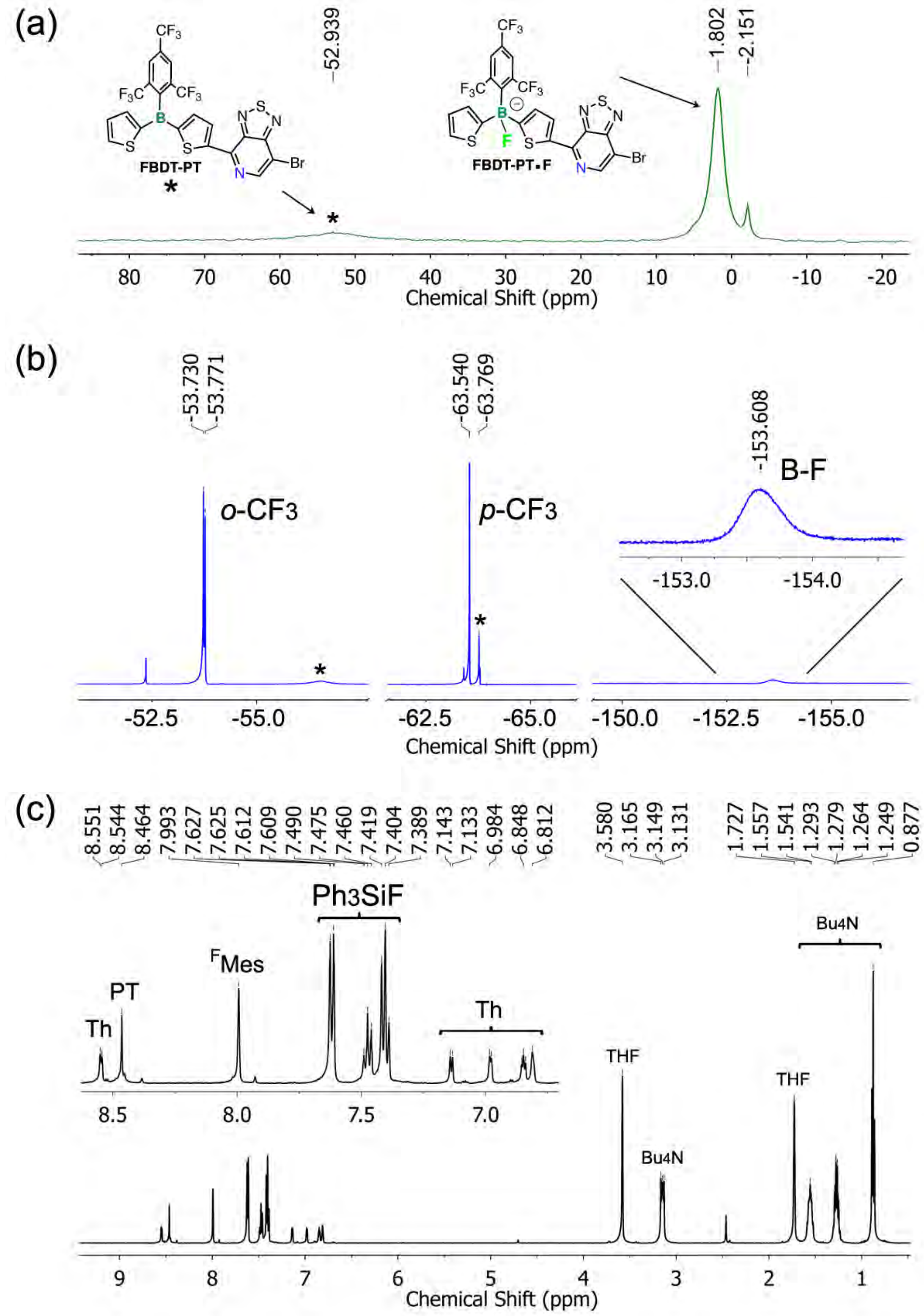

Figure S13. NMR data for **FBDT-PT** (4.1×10^{-3} mmol in 0.37 mL d8-THF) after addition of 1.0 equivalent of $[Bu_4N][Ph_3SiF_2]$ (1.3×10^{-1} M); * denotes a small amount of residual uncomplexed **FBDT-PT** (1.2 equivalents were used when recording the ^{1}H NMR data).

(a)

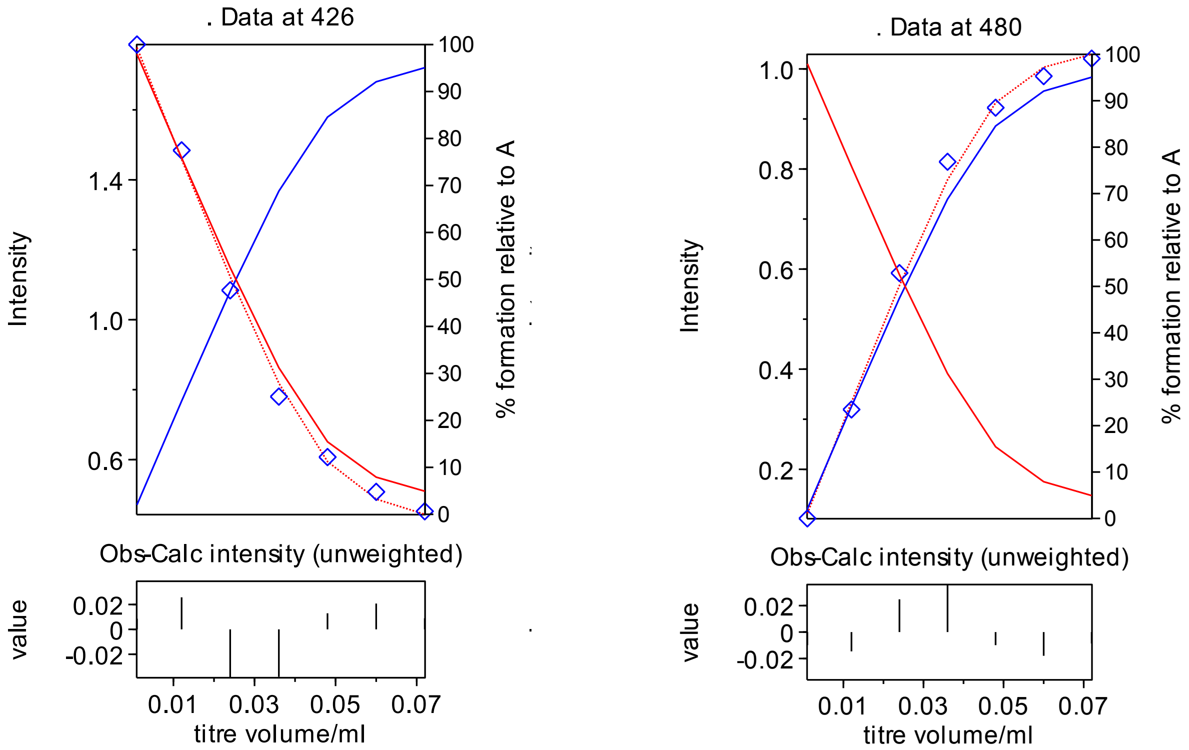

(b)

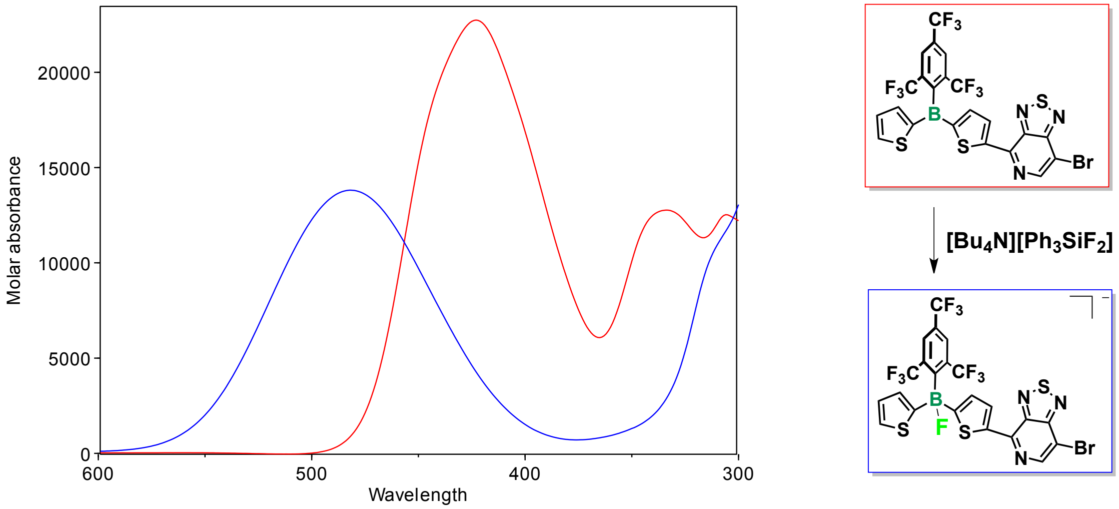

Figure S14. (a) Fits of absorption data (HypSpec™ software) for the titration of **FBDT-PT** (2.4×10^{-4} mmol in 3 mL THF) with $[Bu_4N][Ph_3SiF_2]$ (5.0×10^{-3} M in THF) at λ = 426 nm and at λ = 480 nm using a binding constant of lgK = 5.65. Color Code: **FBDT-PT** (red) and **[FBDT-PT]F** (blue). (b) Calculated absorption spectra of **FBDT-PT** (red) and **[FBDT-PT]F** (blue).

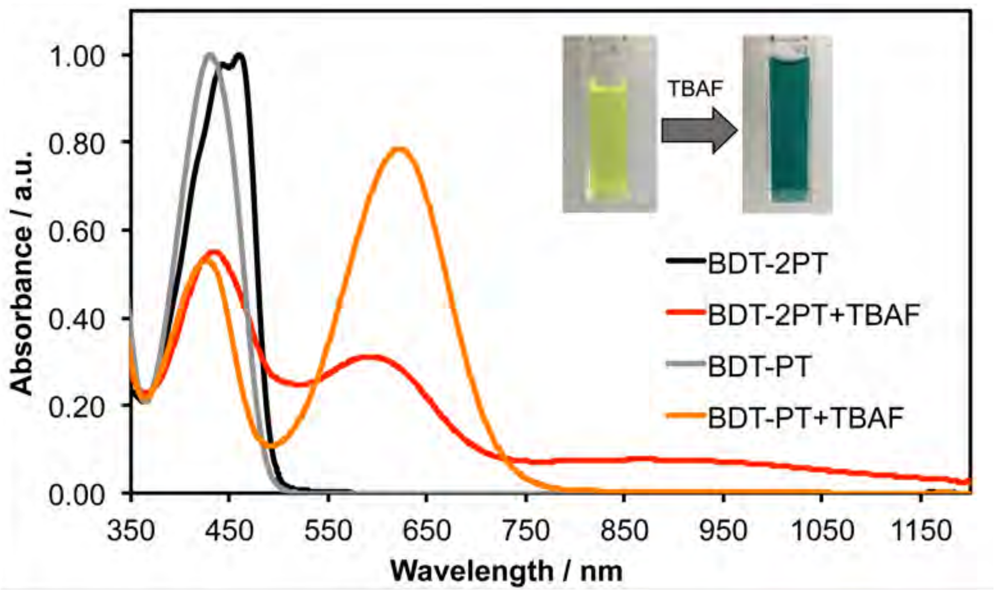

Figure S15. Normalized UV-Vis spectra of **BDT-PT** (1.0×10^{-4} mmol in 3 mL in THF, illustrated with photographs) and **BDT-2PT** (6.4×10^{-5} mmol in 3 mL in THF) solutions before and after addition of a 20 equivalents of TBAF•3H_2O (2.5×10^{-2} M in THF).

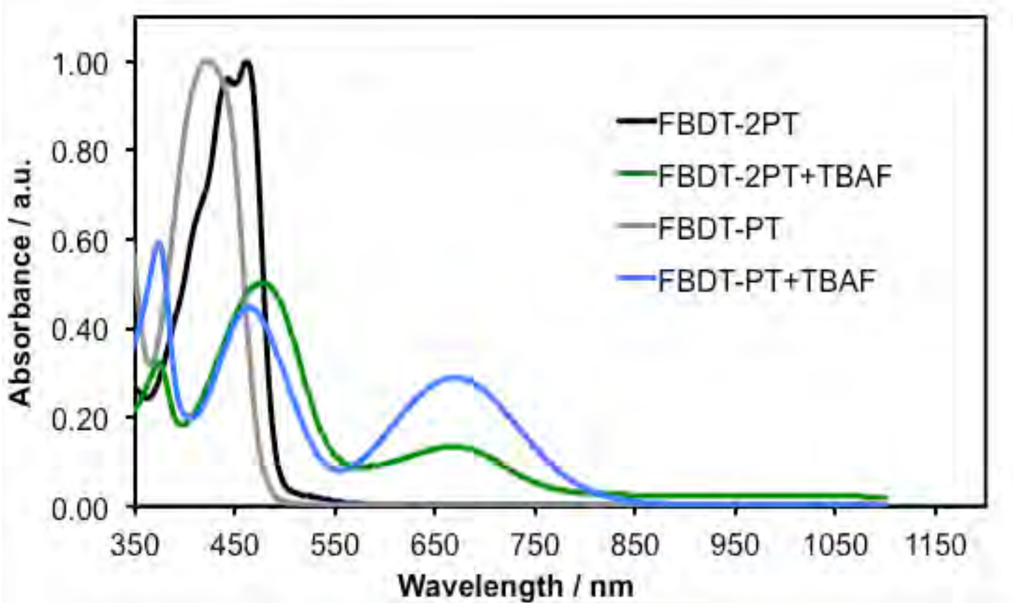

Figure S16. Normalized UV-Vis spectra of **FBDT-PT** (3.6×10^{-4} mmol in 3 mL in THF) and **FBDT-2PT** (1.5×10^{-4} mmol in 3 mL in THF) before and after addition of 2 equivalents of TBAF•3H_2O (1.25×10^{-2} M in THF).

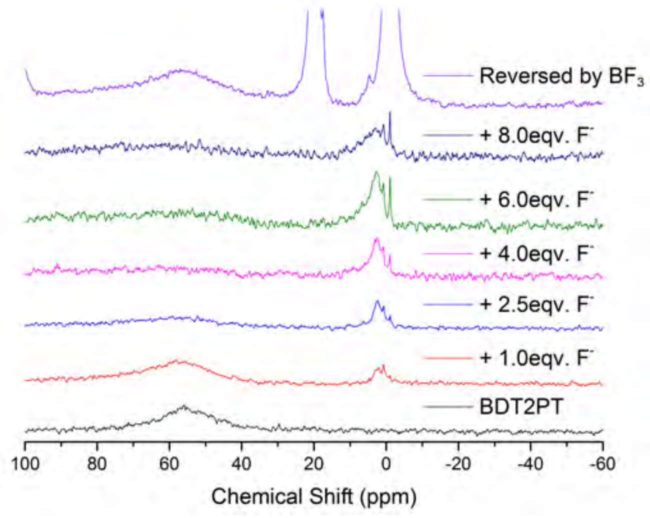

Figure S17. ^{11}B NMR titration experiment of **BDT-2PT** in d8-THF solution. Aliquots of a TBAF•3H_2O solution (2.0×10^{-1} M in d8-THF) were added to **BDT-2PT** (5.0×10^{-3} mmol in 0.55 mL d8-THF).

The signal at ca. +57 ppm in the ^{11}B NMR is assigned to the free borane, the signal at +2 ppm to the fluoride anion complex **BDT-2PT•**F^-. The strong broadening of the ^{11}B NMR resonance at +57 ppm is tentatively attributed to the formation of PT-centered radical anions. Excess BF_3•OEt_2 in THF was added to reverse the anion binding process; regeneration of the free Lewis acid was confirmed by reemergence of the broad peak at ca. +57 ppm.

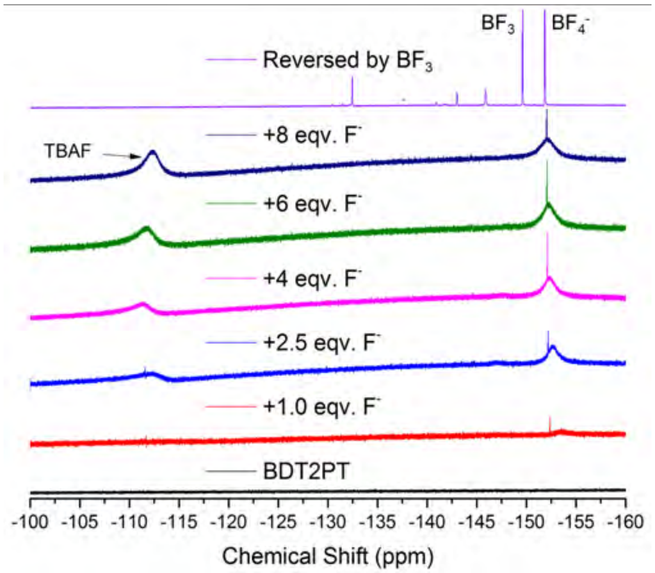

Figure S18. ^{19}F NMR titration experiment of **BDT-2PT** in d8-THF solution. Aliquots of a TBAF•3H_2O solution (2.0×10^{-1} M in d8-THF) were added to **BDT-2PT** (5.0×10^{-3} mmol in 0.55 mL d8-THF).

The broad signal at ca. –152 ppm in the ^{19}F NMR is tentatively assigned to the anion complex **BDT-2PT•**F^-, but could also be due to generation of additional HF_2^-, and that at ca. –112 ppm to unbound fluoride anions. Excess BF_3•OEt_2 was added to reverse the anion binding process, leading to disappearance of both signals.

DFT Calculation Results for Fluoride Anion Complexes

Table S7. Frontier orbital representations (scaling radii of 75%, isovalue = 0.02) and energy levels of borane-PT fluoride anion complexes (structure optimization with B3LYP/6-31+G*, single point energy calculations with B3PW91/6-311+G*). C grey, B blue-green, Br red, F light blue, N blue, S yellow.

	**(BDT-PT)·F$^-$**	**(BDT-2PT)·F$^-$**	**(FBDT-PT)·F$^-$**	**(FBDT-2PT)·F$^-$**
**LUMO+1**	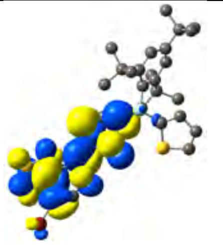 **1.06 eV**	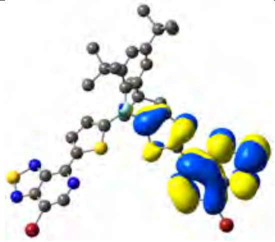 **-1.07 eV**	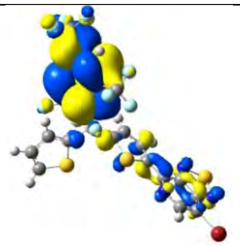 **0.82 eV**	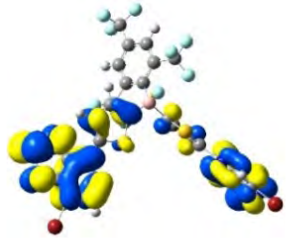 **-1.18 eV**
**LUMO**	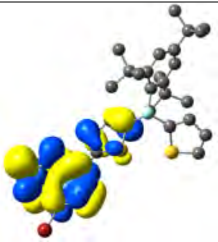 **-0.96 eV**	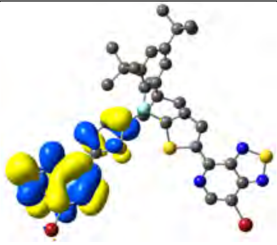 **-1.12 eV**	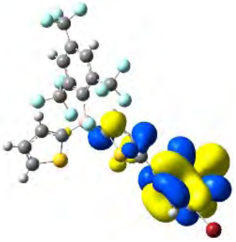 **-1.03 eV**	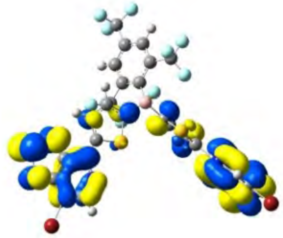 **-1.19 eV**
**HOMO**	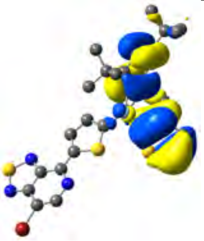 **-3.08 eV**	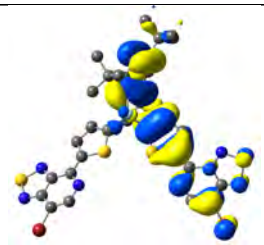 **-3.40 eV**	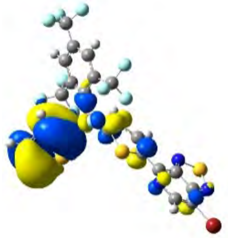 **-3.36 eV**	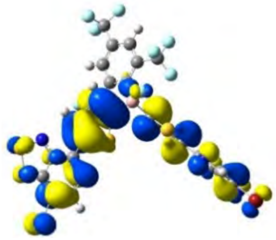 **-3.66 eV**
**HOMO-1**	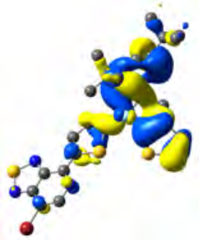 **-3.21 eV**	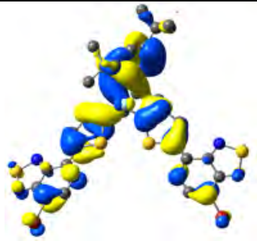 **-3.50 eV**	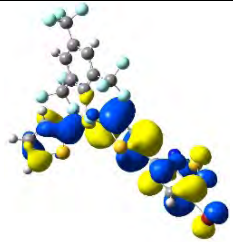 **-3.49 eV**	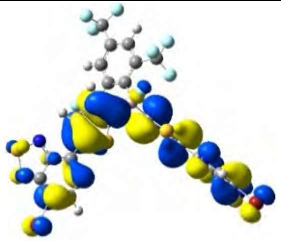 **-3.71 eV**

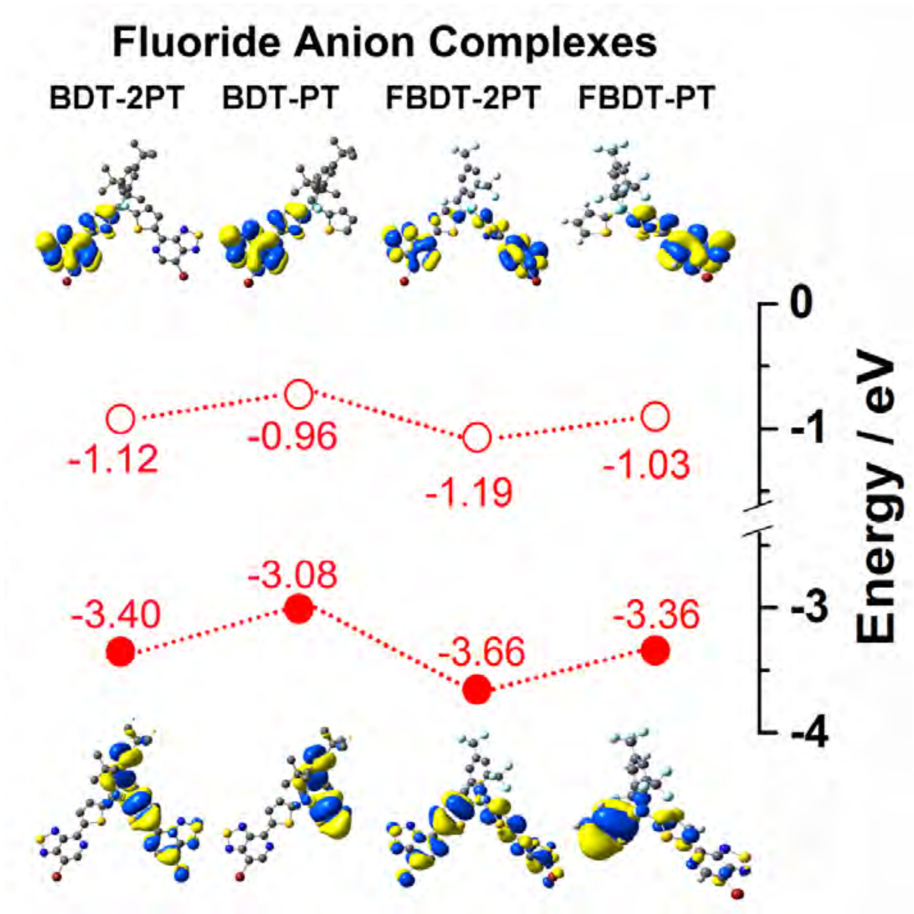

Figure S19. Comparison of HOMO and LUMO orbitals of fluoride anion complexes (structures optimized at B3LYP/6-31+G* level, single point energy calculations at B3PW91/6-311+G**).

Table S8. TD-DFT data of **(BDT-PT)·F$^-$** and **(BDT-2PT)·F$^-$** (wb97xd/6-311+G**).

**(BDT-PT)·F$^-$**			**(BDT-2PT)·F$^-$**		
λ (nm)	Oscillator strength *f*	Assignment (Probability)	λ (nm)	Oscillator strength *f*	Assignment (Probability)
**491.36**	**0.5155**	H-2→L (0.16)	**496.91**	**0.5542**	H-1→L (0.32)
		**H-1→L (0.79)**			**H→L+1 (0.51)**
355.31	0.0051	H-5→L (0.76)	**473.17**	**0.4339**	**H-1→L (0.56)**
		H-4→L (0.14)			H→L+1 (0.31)
293.36	0.1305	H-7→L (0.46)	347.84	0.0059	H-5→L (0.29)
		H-1→L+1 (0.22)			H-4→L (0.61)
285.68	0.0014	H-3→L (0.85)	342.37	0.0035	H-5→L+1 (0.44)
277.99	0.0028	H-5→L (0.14)			H-4→L+1 (0.41)
		H-4→L (0.72)	318.46	0.0014	H-2→L (0.17)
272.95	0.2086	H-7→L (0.21)			H-2→L+1 (0.26)
		H-1→L+1 (0.46)			H→L (0.40)

Table S9. TD-DFT data of **(FBDT-PT)·F$^-$** and **(FBDT-2PT)·F$^-$** (wb97xd/6-311+G**).

**(FBDT-PT)·F$^-$**			**(FBDT-2PT)·F$^-$**		
λ (nm)	Oscillator strength *f*	Assignment (Probability)	λ (nm)	Oscillator strength *f*	Assignment (Probability)
**478.05**	**0.4899**	**H→L (0.89)**	**481.20**	**0.5751**	**H→L (0.47)**
350.04	0.0055	H-3→L (0.77)	**458.37**	**0.3790**	**H→L+1 (0.48)**
		H-2→L (0.16)	343.29	0.0063	H-2→L (0.36)
308.60	0.0006	H-1→L (0.85)	340.78	0.0047	H-3→L (0.13)
288.70	0.1280	H-5→L (0.52)			H-3→L+1 (0.31)
		H→L+4 (0.17)			H-2→L (0.22)
269.87	0.1543	H-10→L (0.19)			H-2→L+1 (0.27)
		H-5→L (0.22)	302.71	0.0010	H-1→L (0.15)
		H→L+1 (0.11)			H-1→L+1 (0.38)
		H→L+4 (0.27)			H→L (0.16)
264.90	0.2010	H-10→L (0.36)			H→L+1 (0.19)
		H→L+5 (0.28)	299.56	0.0008	H-1→L+1 (0.11)
					H→L (0.31)
			289.00	0.1330	H-6→L+1 (0.17)
					H-5→L (0.13)
			285.18	0.0820	H-6→L (0.14)
					H-6→L+1 (0.20)

Photophysical, Electrochemical and DFT Calculation Results for BDT-2PTTh

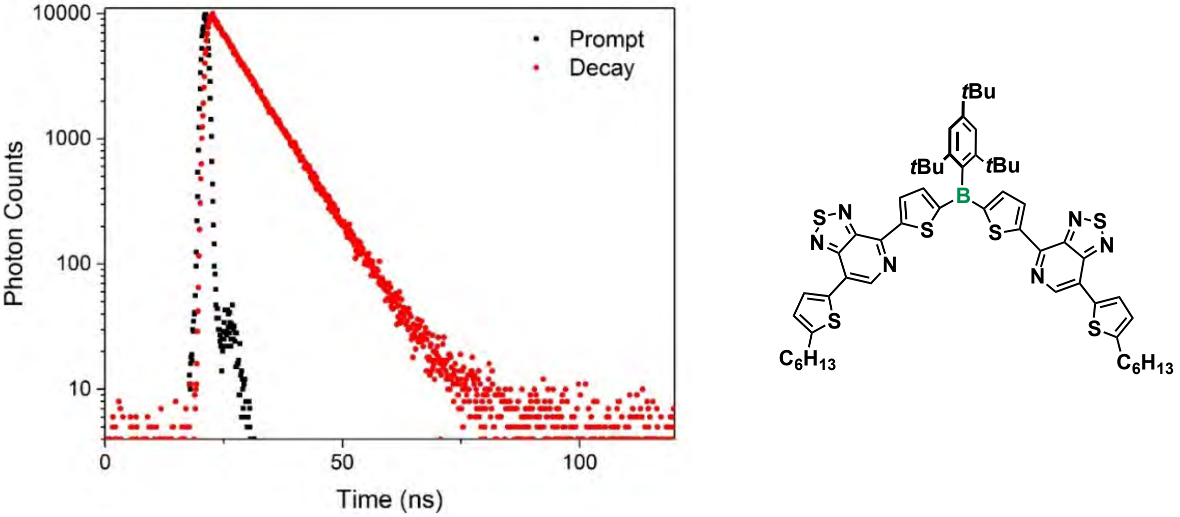

Figure S20. Fluorescence decay plot for **BDT-2PTTh** in degassed THF (1×10^{-5} mol L^{-1}), excited with 388 nm nanoLED.

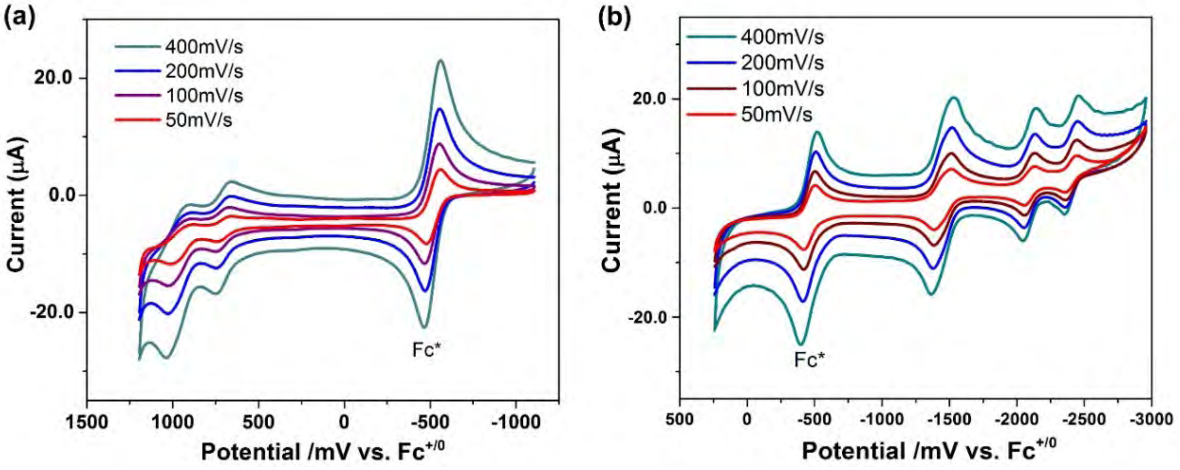

Figure S21. Cyclic voltammetry data of **BDT-2PTTh** in (a) DCM and (b) THF containing 0.1M $Bu_4N[PF_6]$ (1×10^{-3} mol L^{-1}). Decamethylferrocene (Fc*) was used as the internal reference and the data converted to $E(Fc^{0/+}) = 0$ V using the equation $E(Fc^{*0/+}) = E(Fc^{0/+}) - 0.50$ V. $E_{ox1} = 0.71$V ($\Delta E = 71$ mV); $E_{ox2} = 0.97$ V ($\Delta E = 117$ mV); E_{red1}(PT) = –1.42 V, –1.48 V (ΔE: not determined due to overlap of redox waves); E_{red2}(boron) = –2.09 V ($\Delta E = 73$ mV); E_{red3}(PT) = –2.40 V ($\Delta E = 88$ mV).

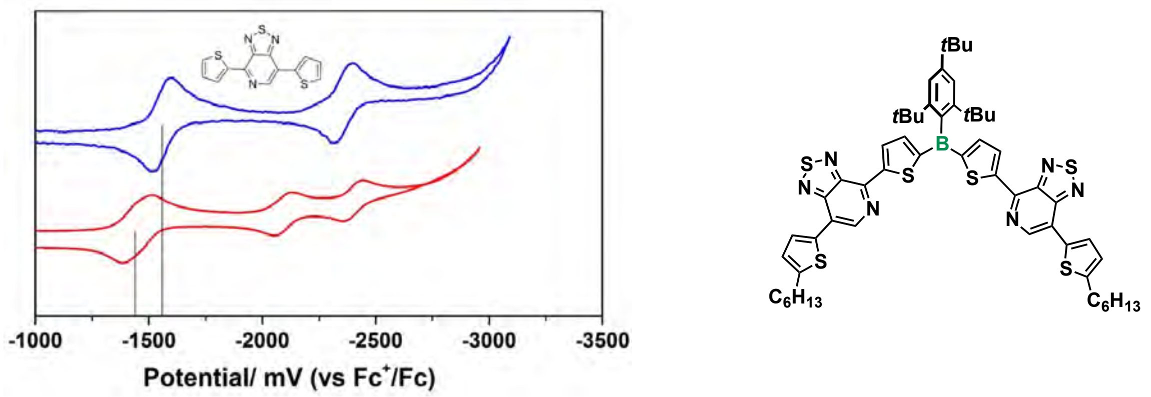

Figure S22. Comparison of cyclic voltammetry data of **BDT-2PTTh** (red) and dithienyl pyridalthiadiazole (blue) in THF, 0.1M $Bu_4N[PF_6]$ (1×10^{-3} mol L^{-1}; vs. $Fc^{+/0}$). $E_{red1} = -1.56$ (ΔE = 74 mV); $E_{red2} = -2.36$ (ΔE = 74 mV).

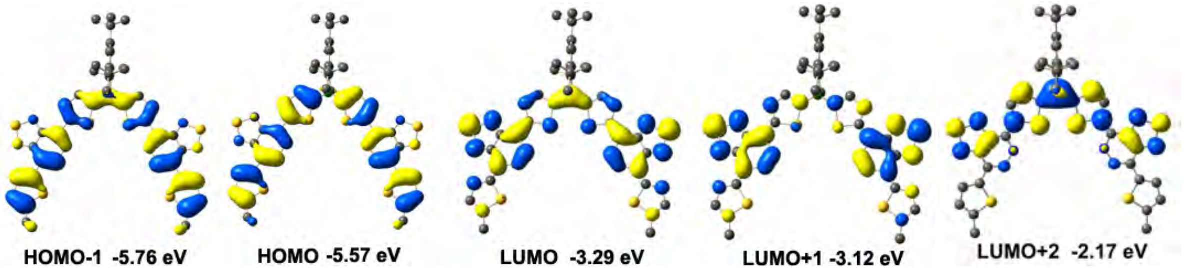

Figure S23. Frontier orbital representations (scaling radii of 75%, isovalue = 0.02) and energy levels of **BDT-2PTTh** (simplified structure with methyl in place of hexyl group; structure optimization with B3LYP/6-31+G*, single point energy calculations with B3PW91/6-311+G*). C grey, B blue-green, N blue, S yellow.

Mobility Measurements for BDT-2PTTh in Electron-Only Devices

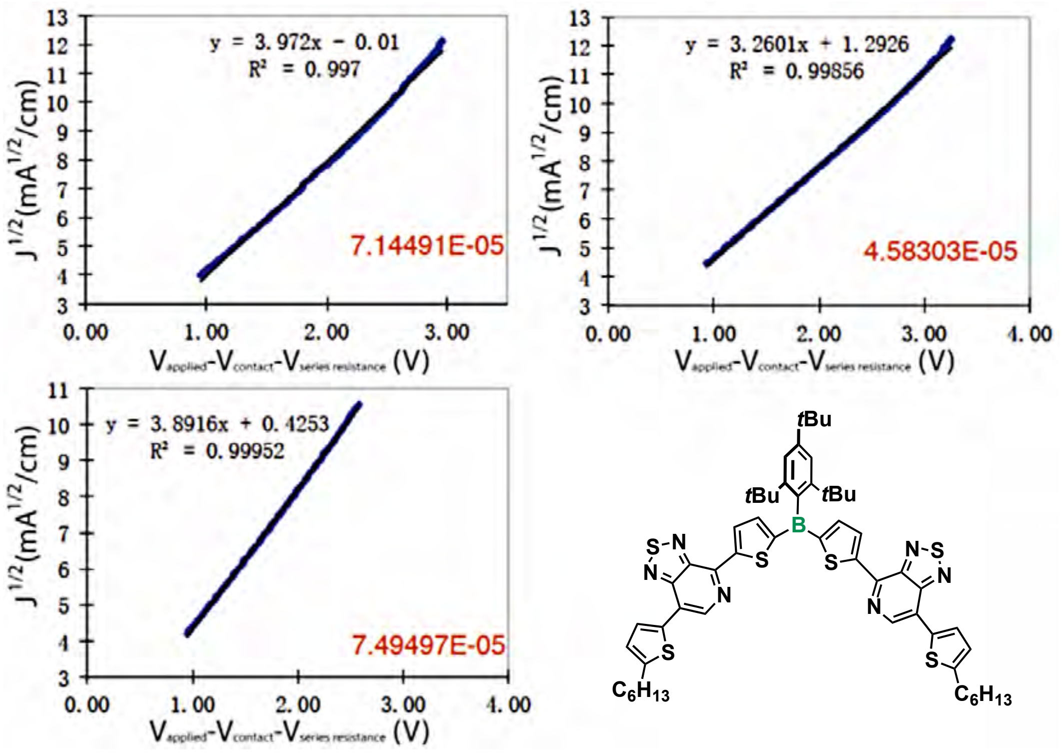

Figure S24. Electron mobility data of **BDT-2PTTh** from electron only devices with a layer thickness of 213 nm.

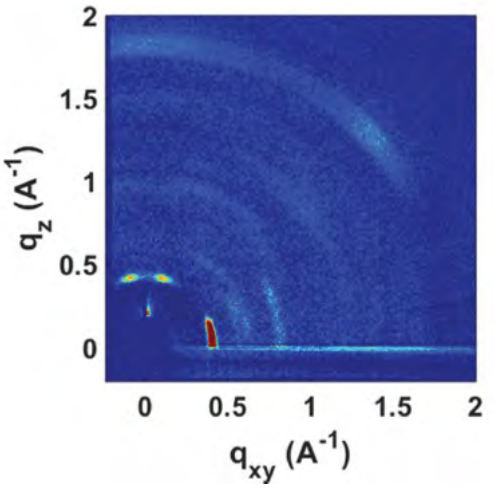

Figure S25. GI-WAXS pattern for an annealed thin film of **BDT-2PTTh** showing limited and diffused reflections consistent with an amorphous structure.

NMR and Mass Spectrometry Data for New Compounds

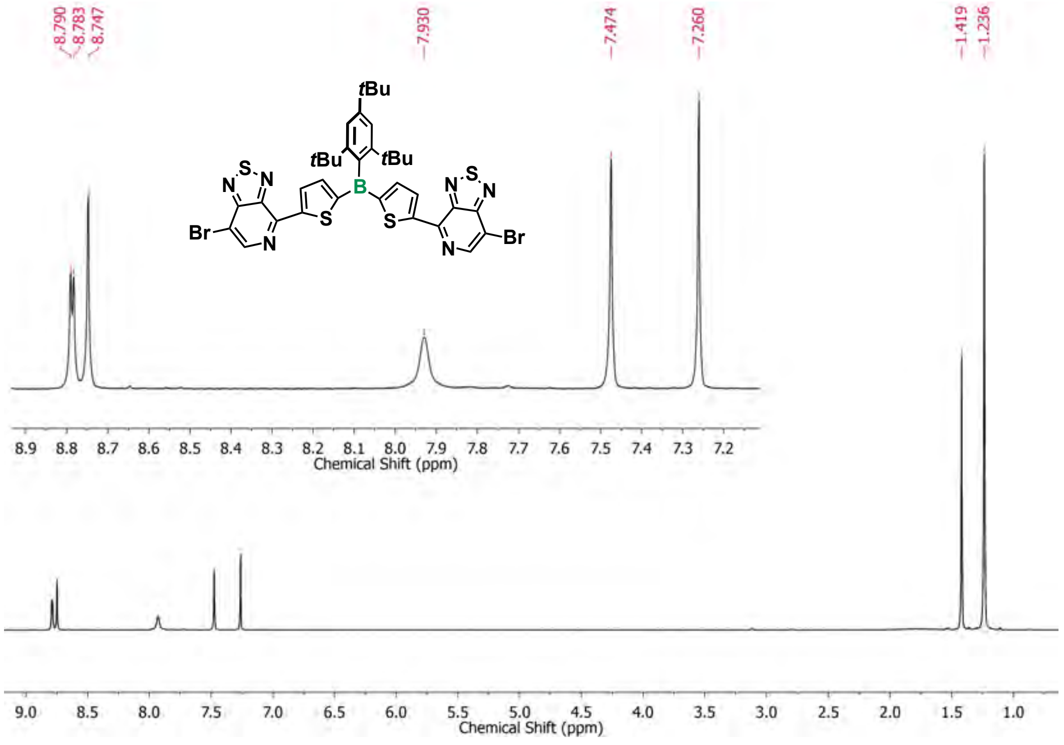

Figure S26. ^{1}H NMR ($CDCl_3$, 25°C) of **BDT-2PT.**

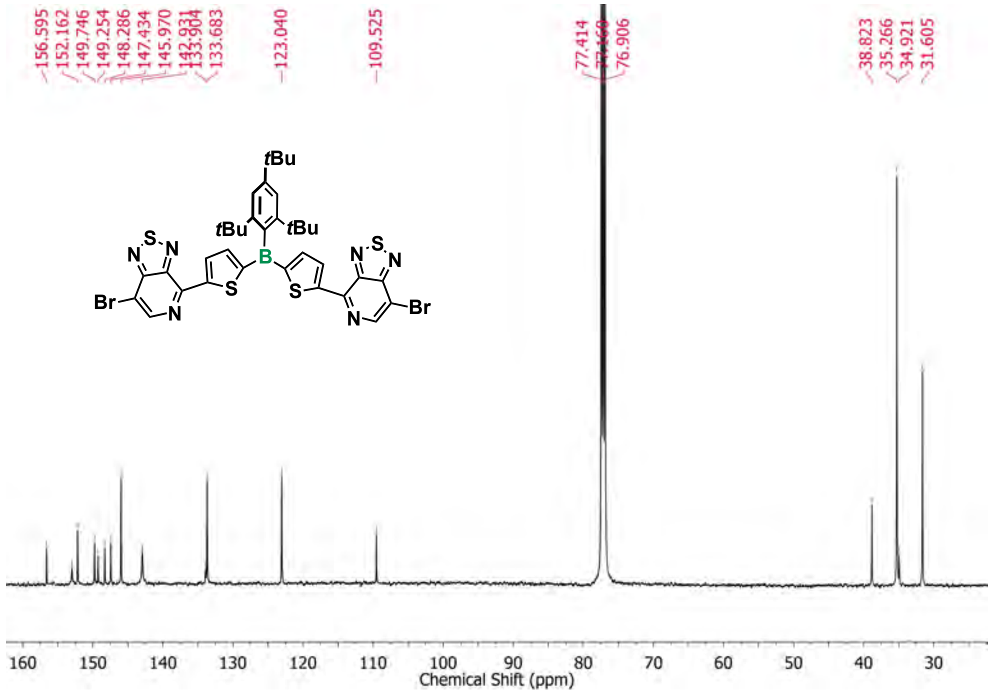

Figure S27. ^{13}C NMR ($CDCl_3$, 25°C) of **BDT-2PT.**

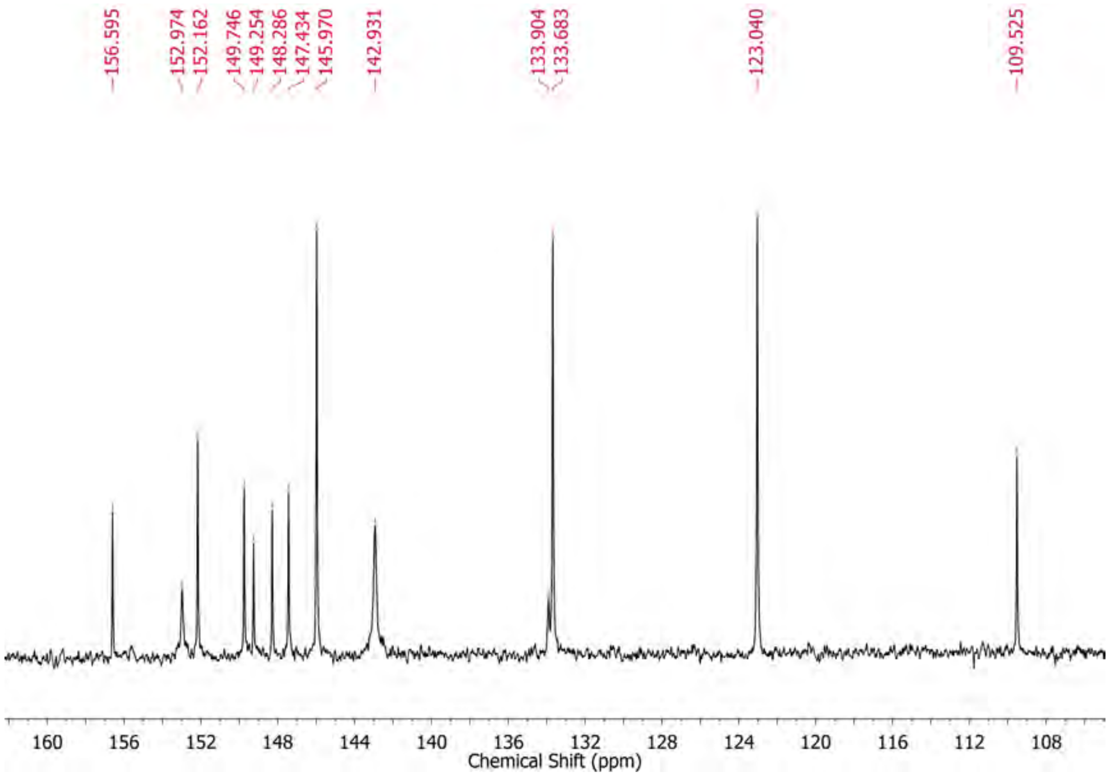

Figure S28. ^{13}C NMR ($CDCl_3$, 25°C) of **BDT-2PT** (aromatic region).

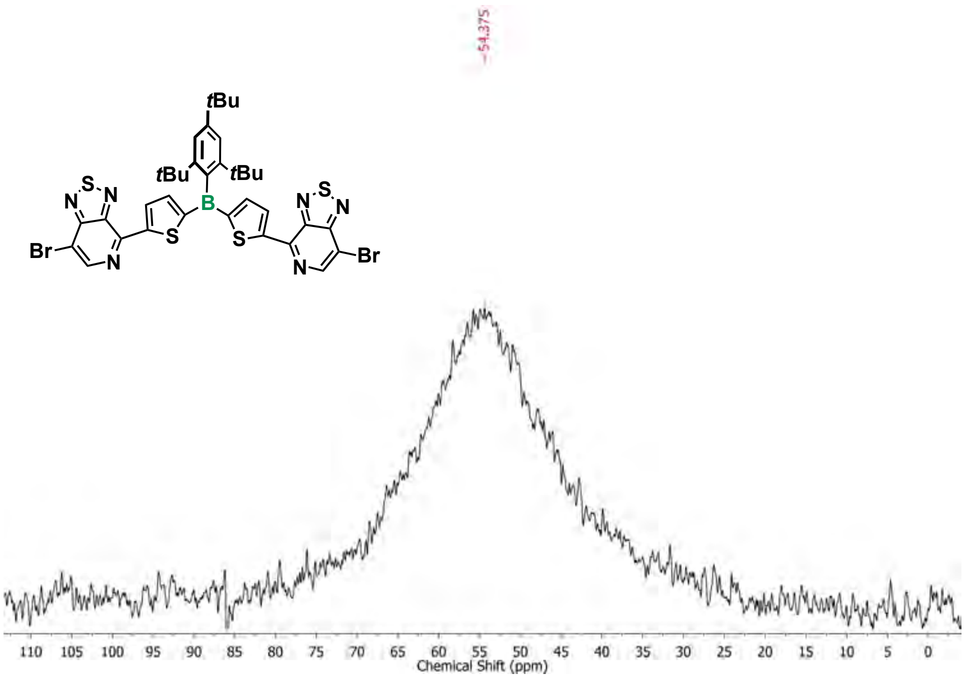

Figure S29. ^{11}B NMR ($CDCl_3$, 25°C) of **BDT-2PT.**

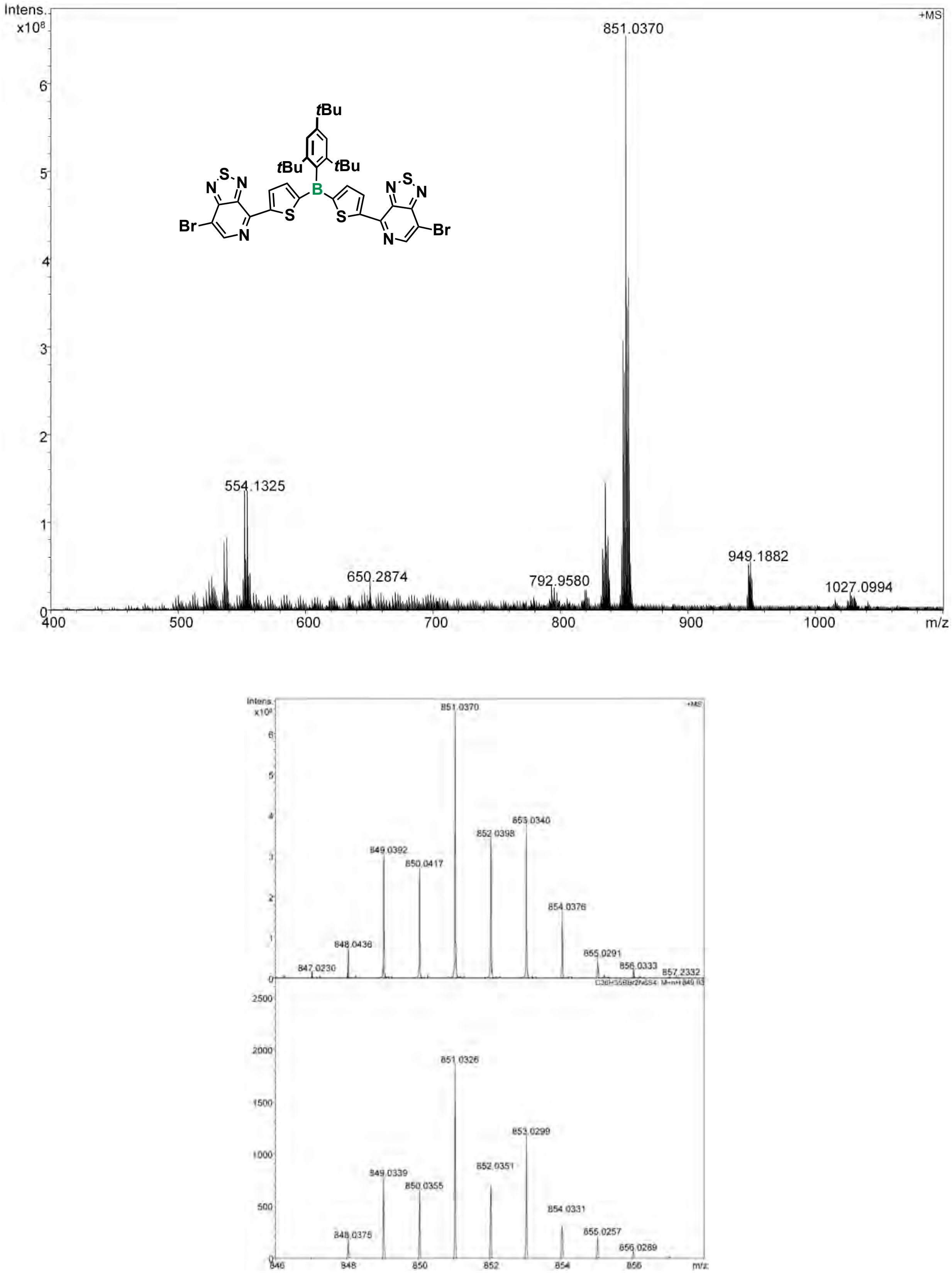

Figure S30. MALDI-MS spectrum of **BDT-2PT** (pos. mode).

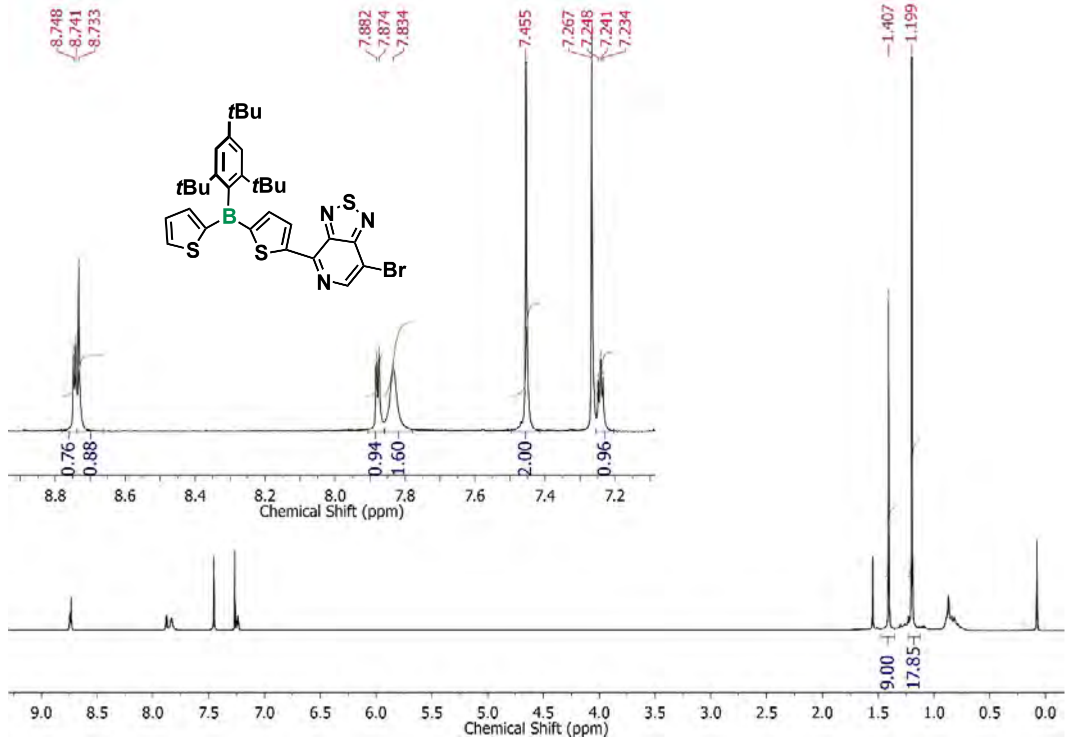

Figure S31. ^{1}H NMR ($CDCl_3$, 25°C) of **BDT-PT** (aromatic region as inset).

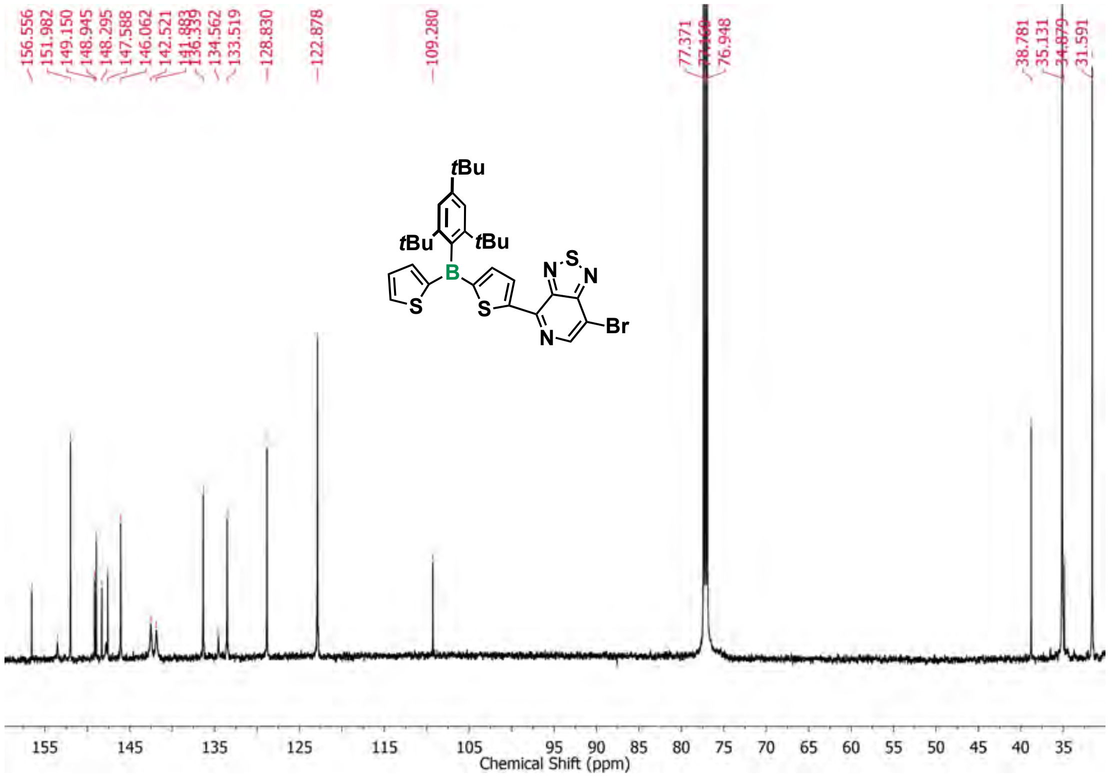

Figure S32. ^{13}C NMR ($CDCl_3$, 25°C) of **BDT-PT.**

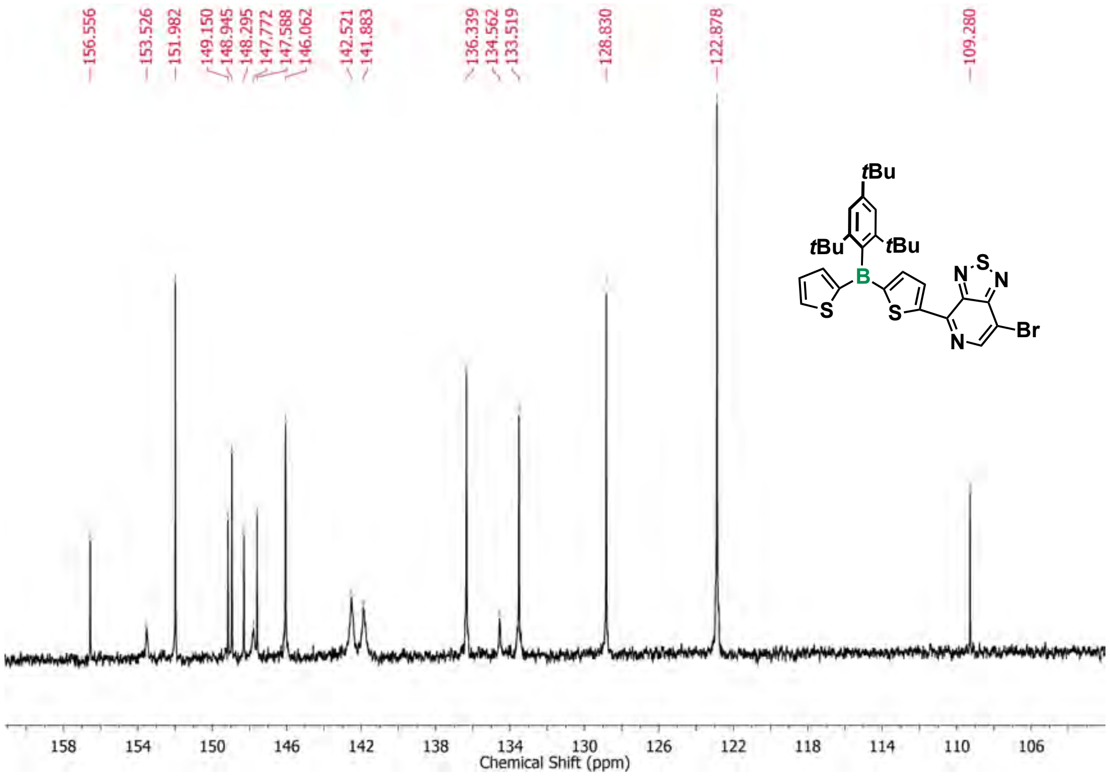

Figure S33. ^{13}C NMR ($CDCl_3$, 25°C) of **BDT-PT** (aromatic region).

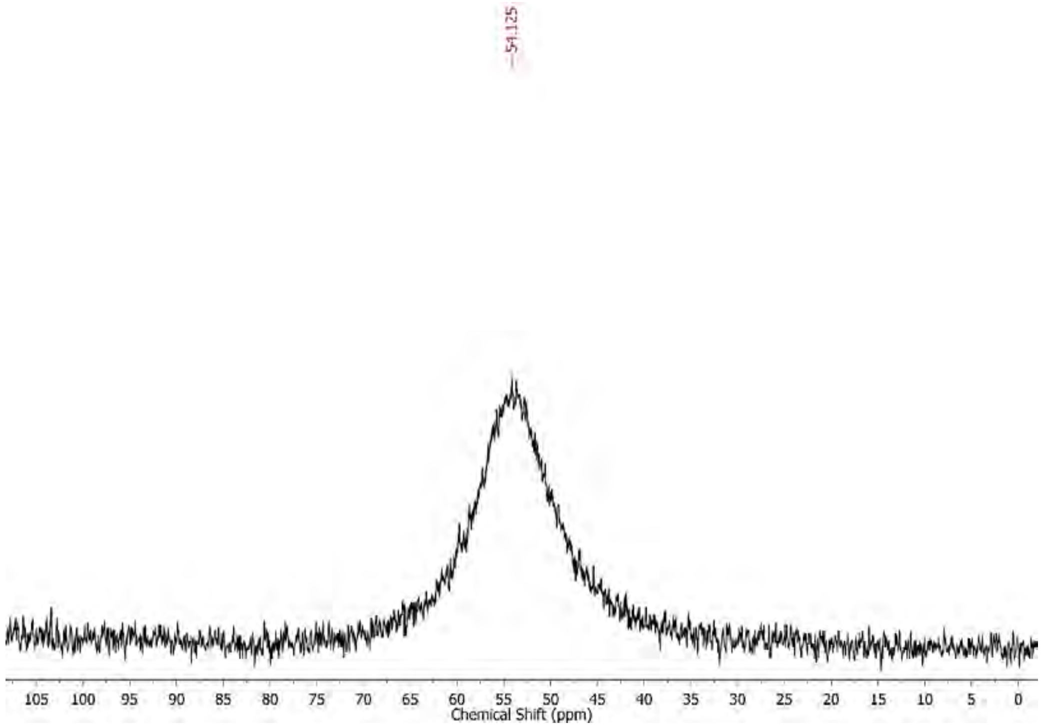

Figure S34. ^{11}B NMR ($CDCl_3$, 25°C) of **BDT-PT.**

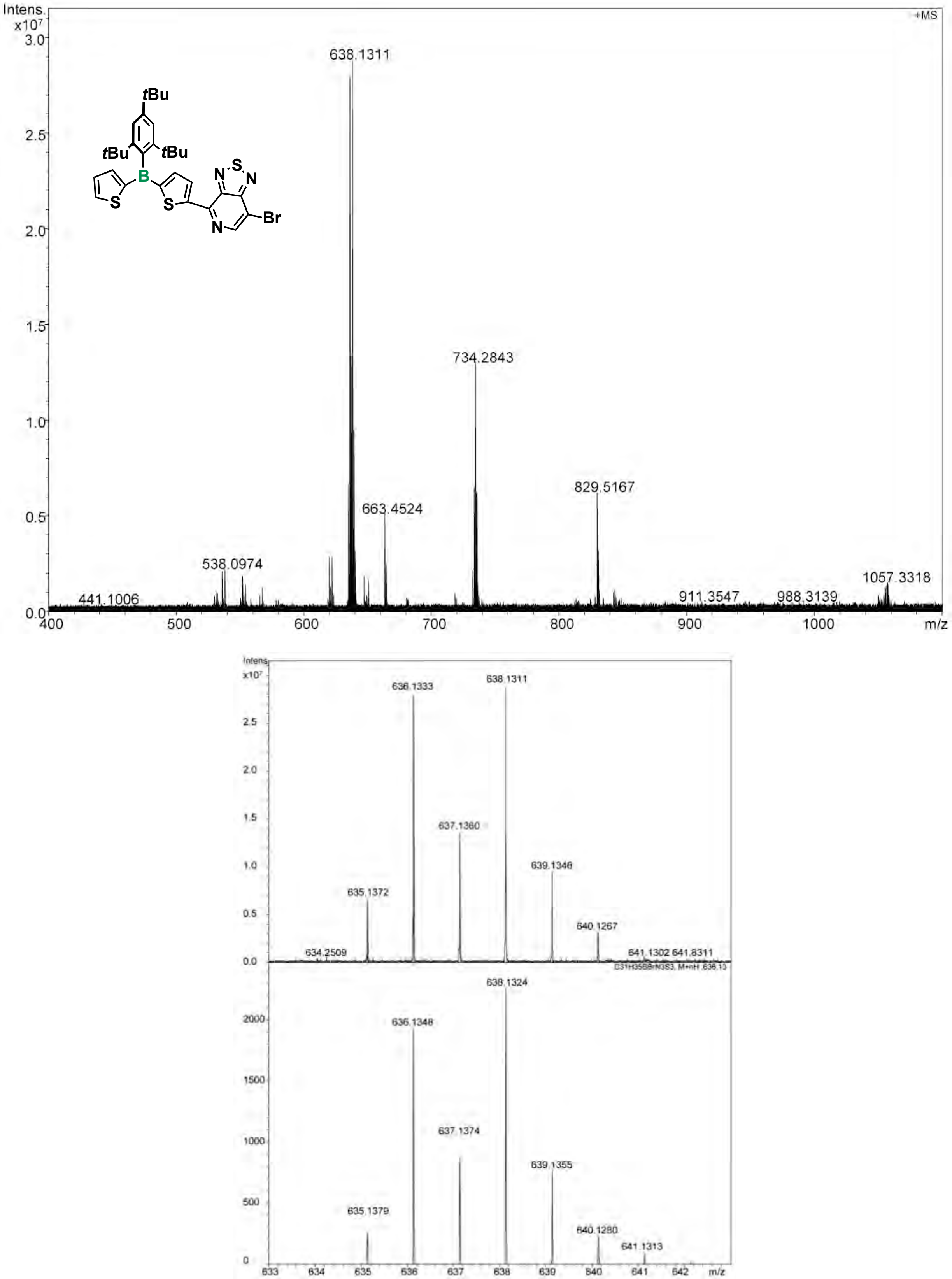

Figure S35. MALDI-MS of **BDT-PT** (pos. mode).

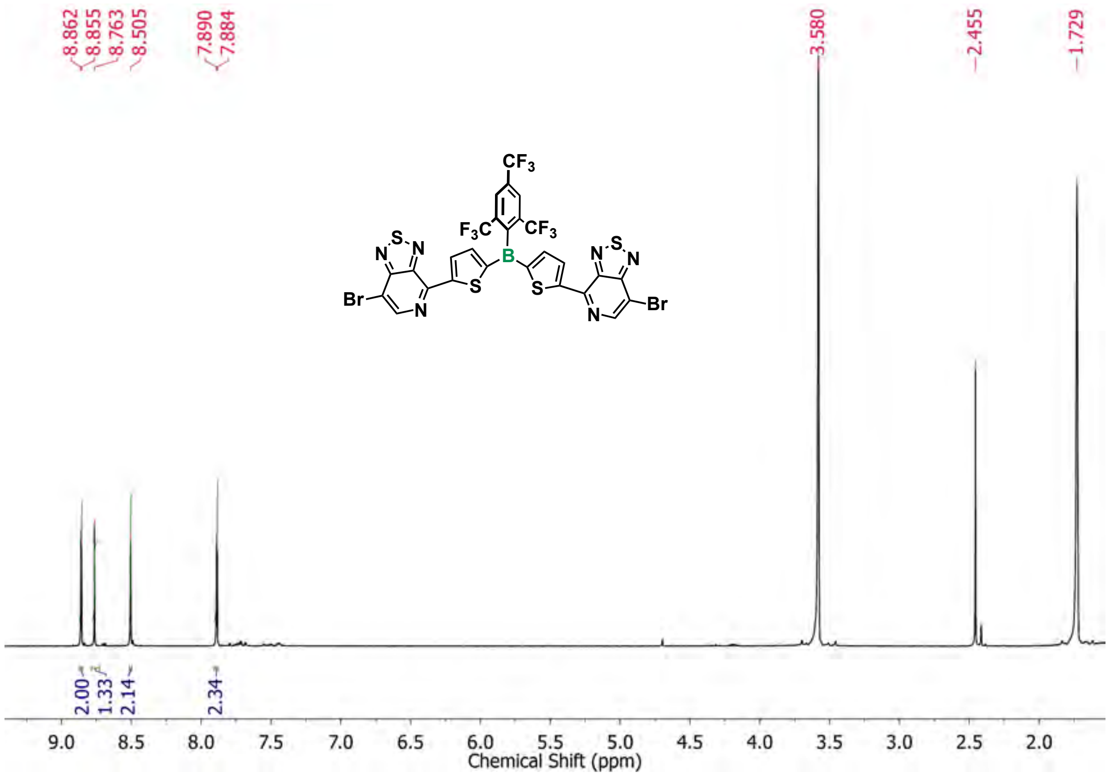

Figure S36. ^{1}H NMR (d8-THF, 25°C) of **FBDT-2PT.**

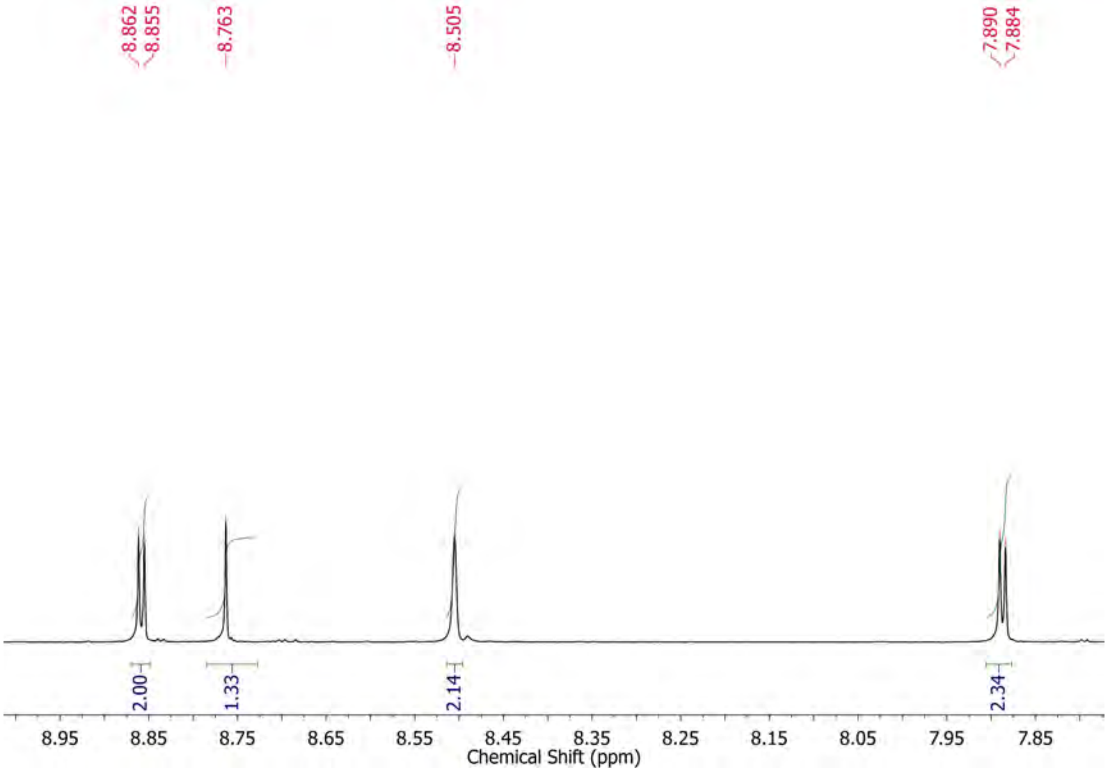

Figure S37. ^{1}H NMR (d8-THF, 25°C) of **FBDT-2PT** (aromatic region).

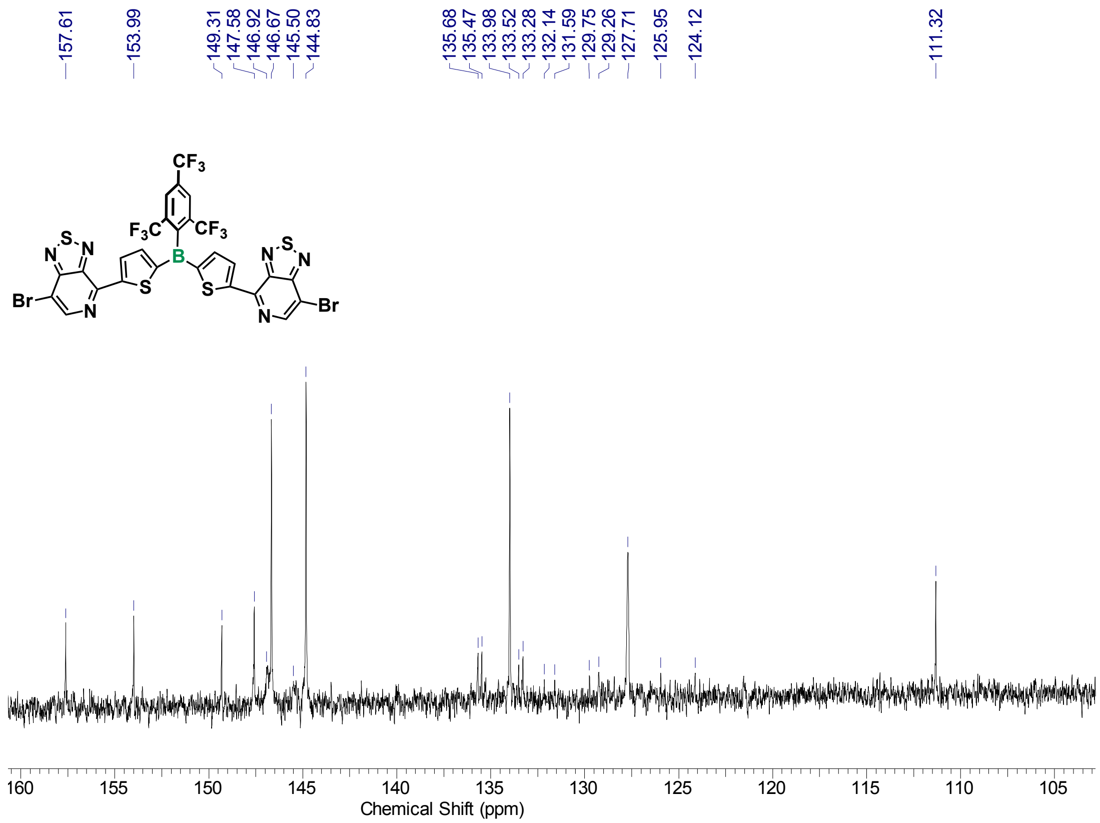

Figure S38. ^{13}C NMR (d8-THF, 25°C) of **FBDT-2PT.**

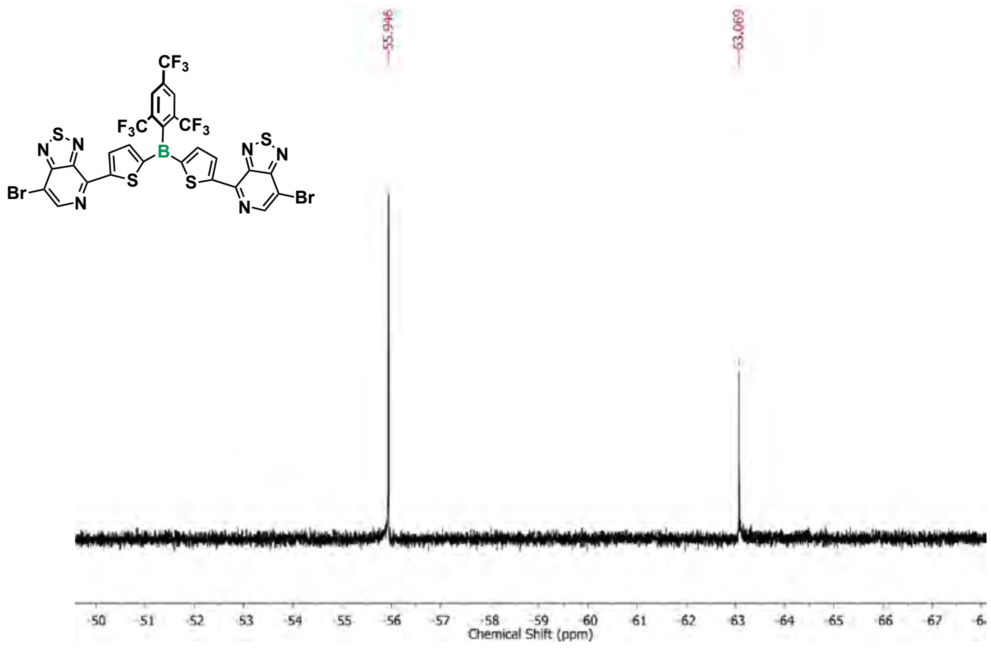

Figure S39. ^{19}F NMR ($CDCl_3$, 25°C) of **FBDT-2PT.**

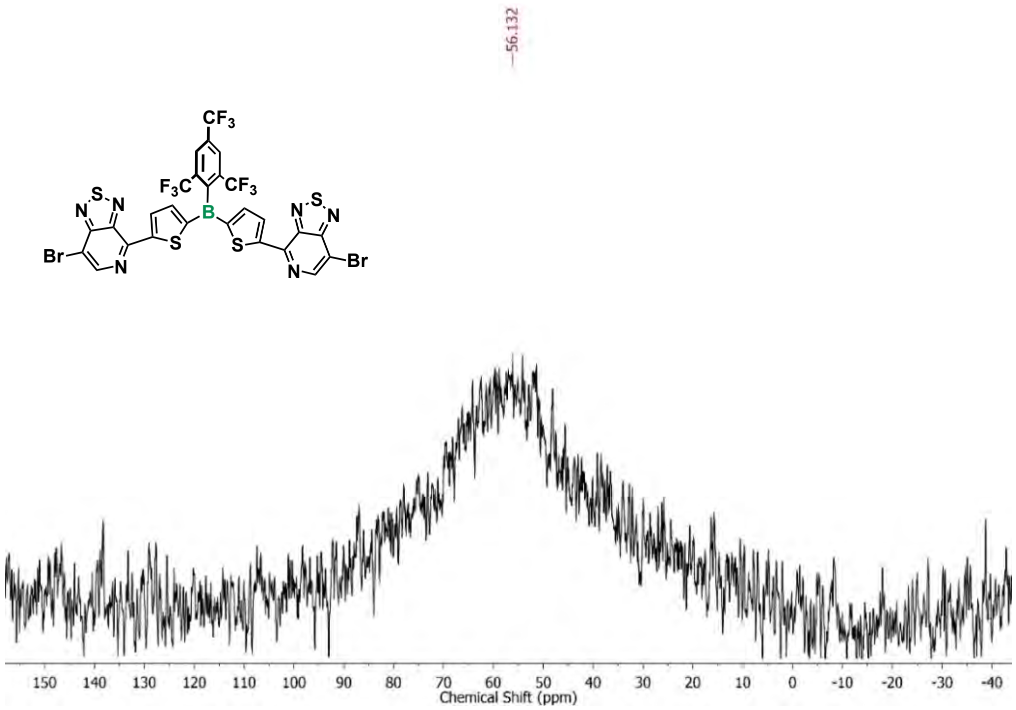

Figure S40. ^{11}B NMR (d8-THF, 25°C) of **FBDT-2PT.**

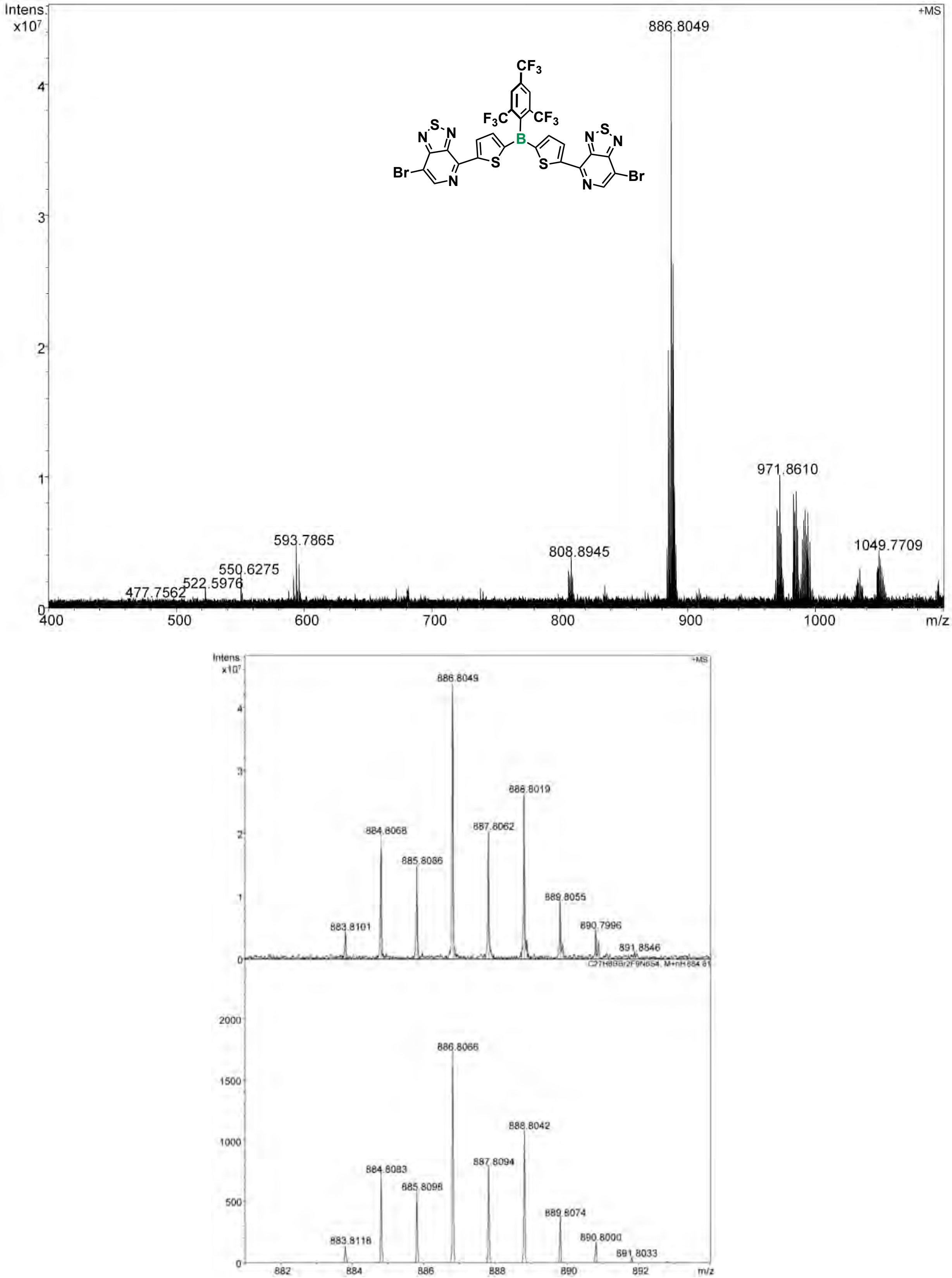

Figure S41. MALDI-MS of **FBDT-2PT** (pos. mode).

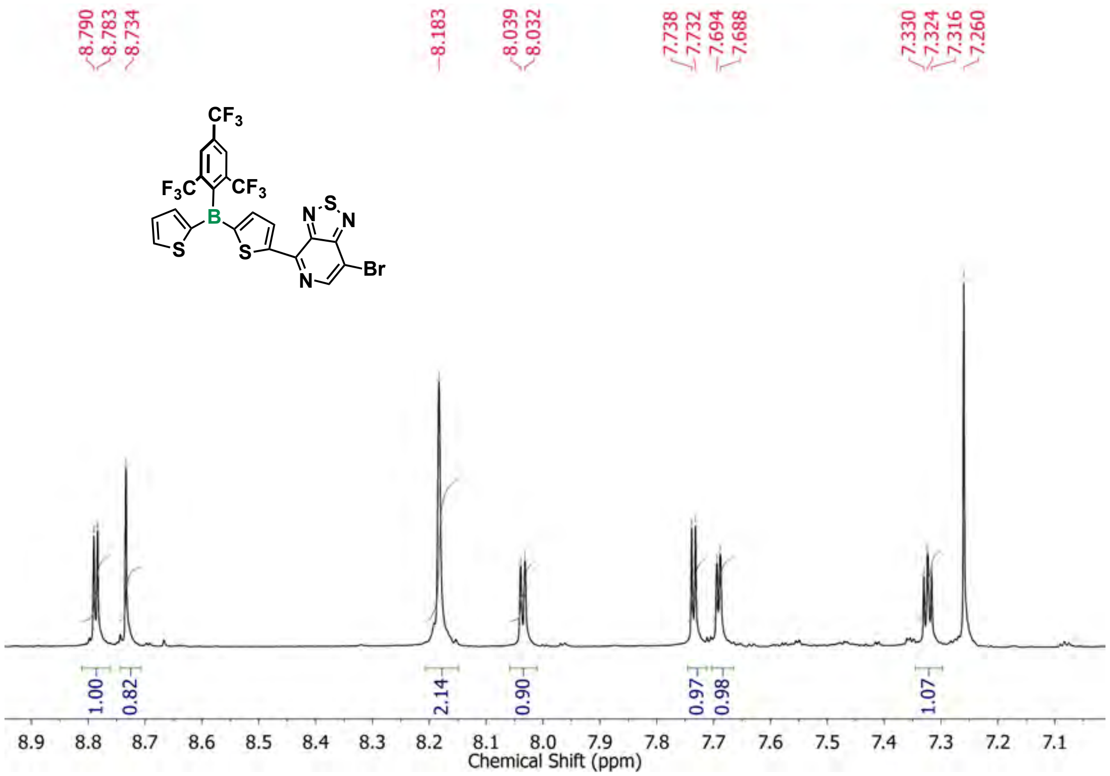

Figure S42. ^{1}H NMR ($CDCl_3$, 25°C) of **FBDT-PT.**

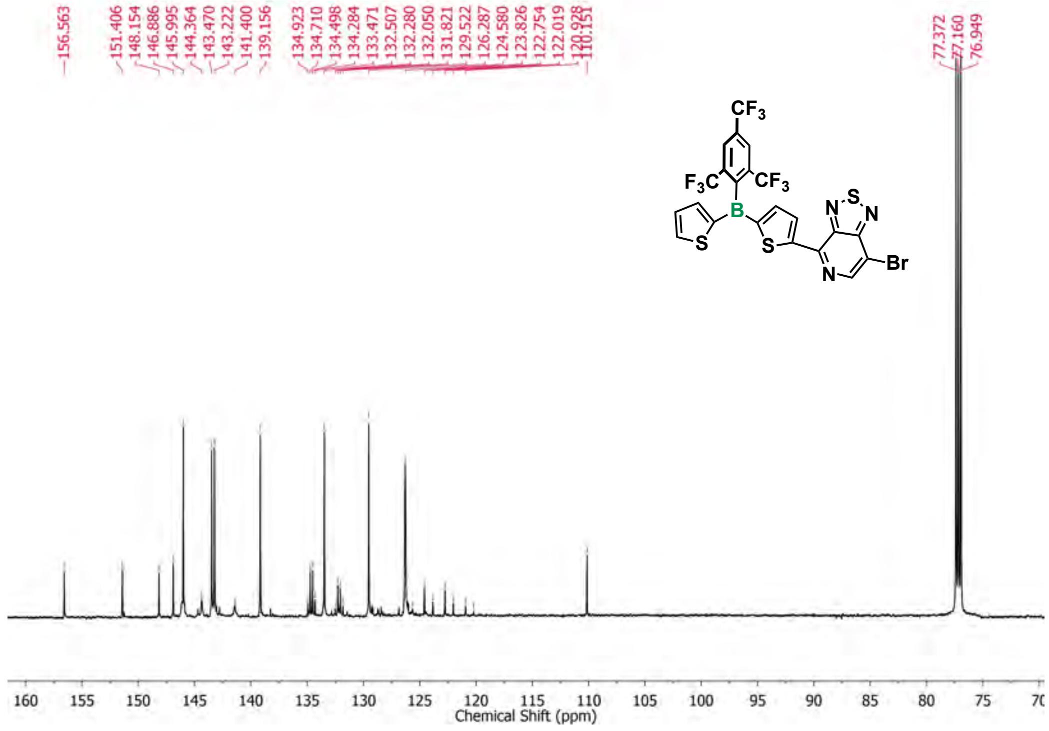

Figure S43. ^{13}C NMR ($CDCl_3$, 25°C) of **FBDT-PT.**

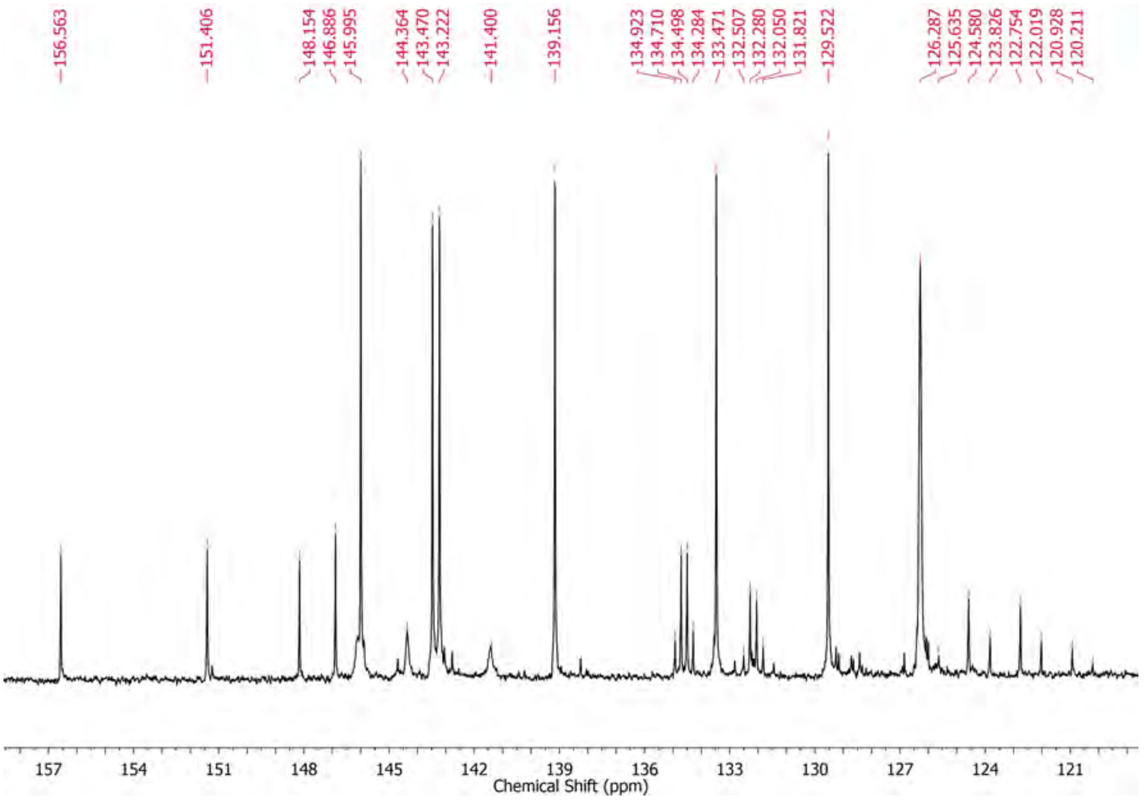

Figure S44. ^{13}C NMR ($CDCl_3$, 25°C) of **FBDT-PT** (aromatic region).

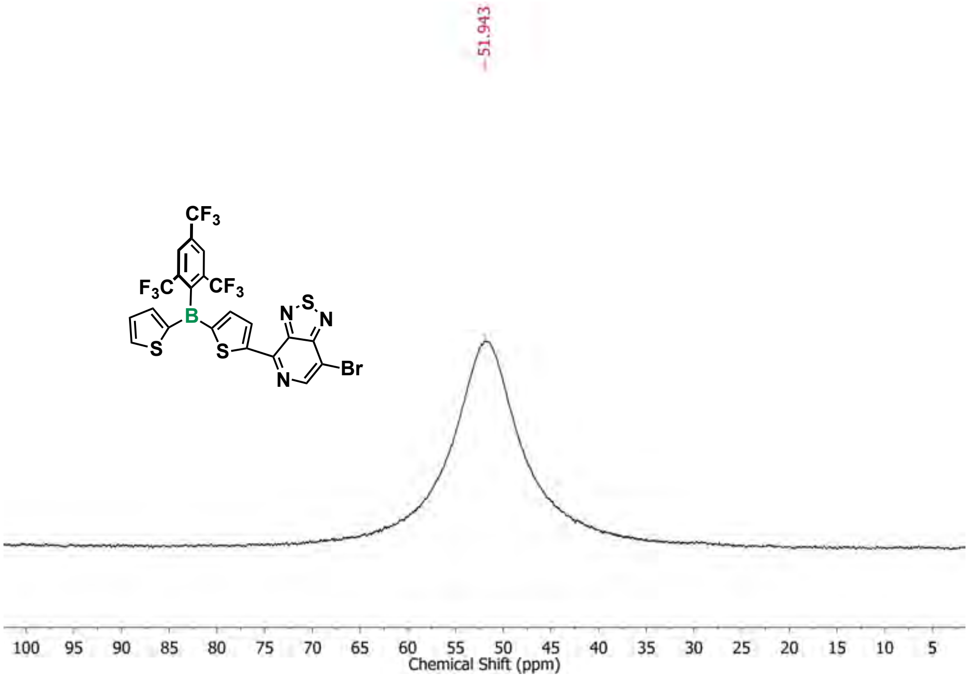

Figure S45. ^{11}B NMR ($CDCl_3$, 25°C) of **FBDT-PT.**

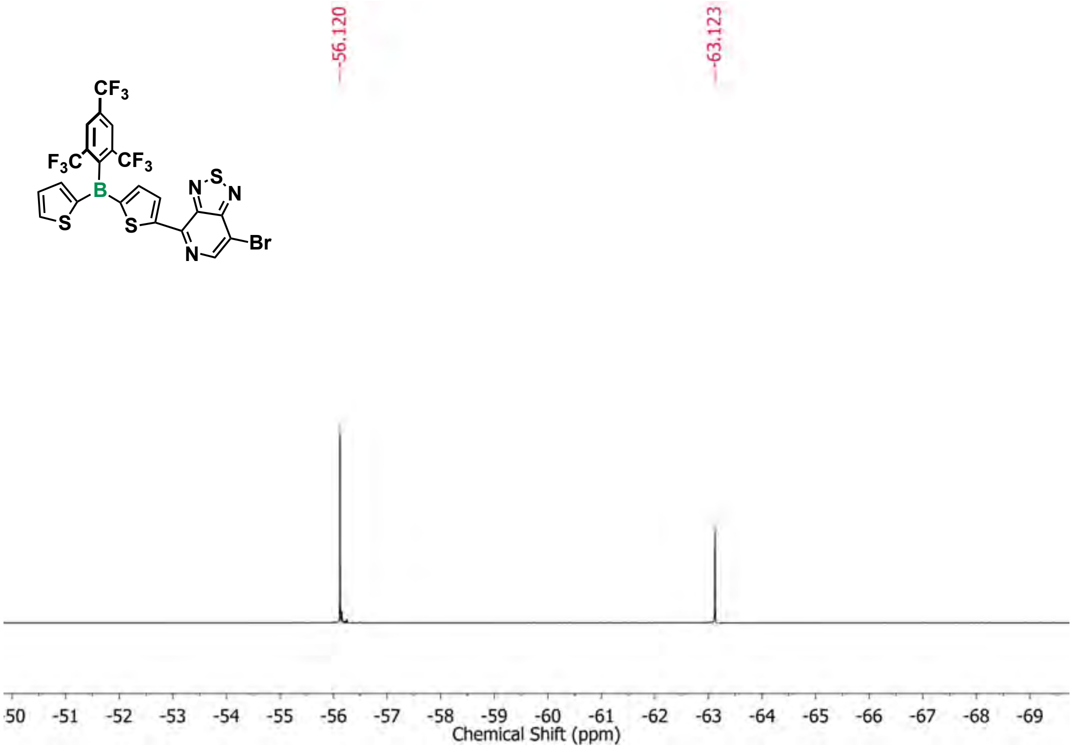

Figure S46. ^{19}F NMR ($CDCl_3$, 25°C) of **FBDT-PT.**

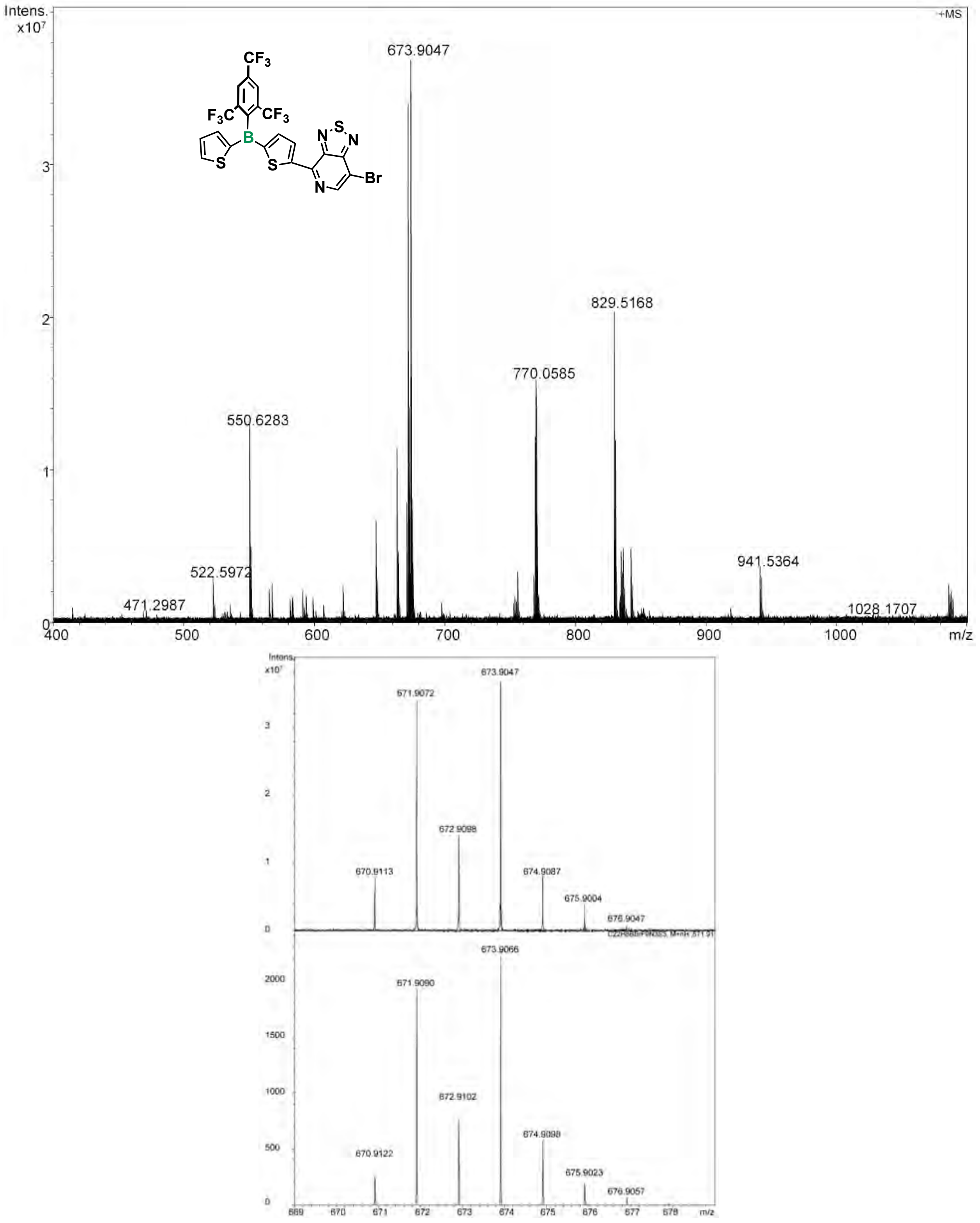

Figure S47. MALDI-MS of **FBDT-PT** (pos. mode).

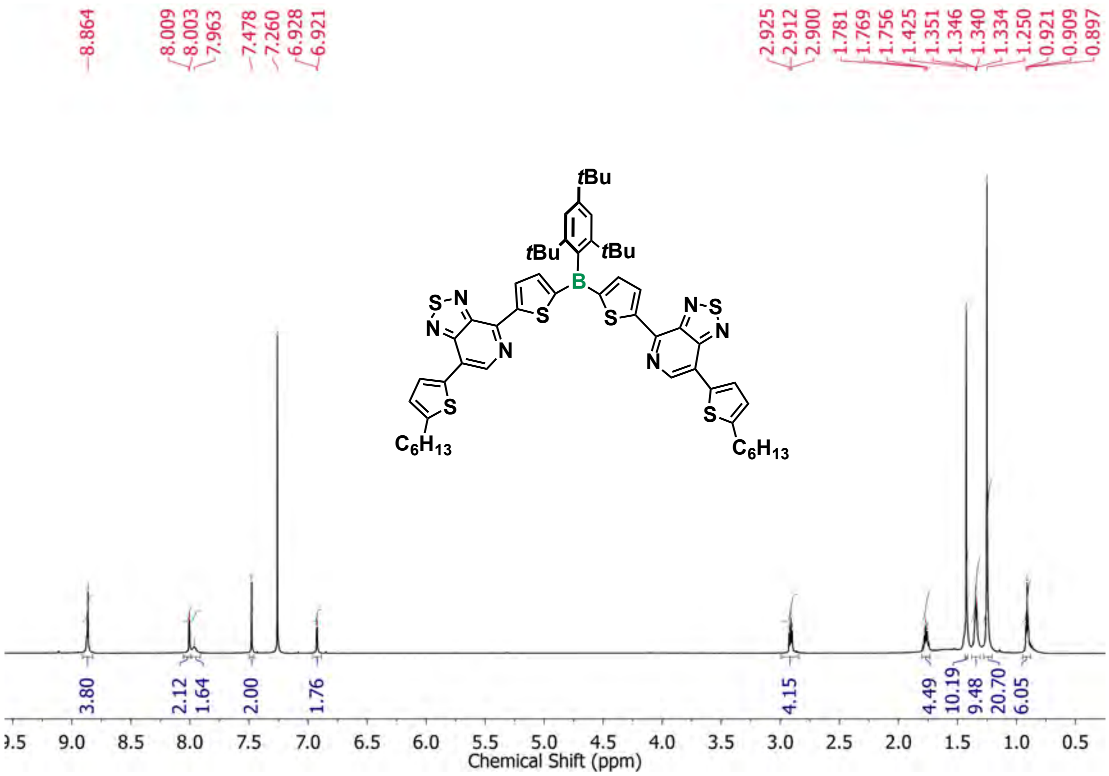

Figure S48. ^{1}H NMR ($CDCl_3$, 25°C) of **BDT-2PTTh.**

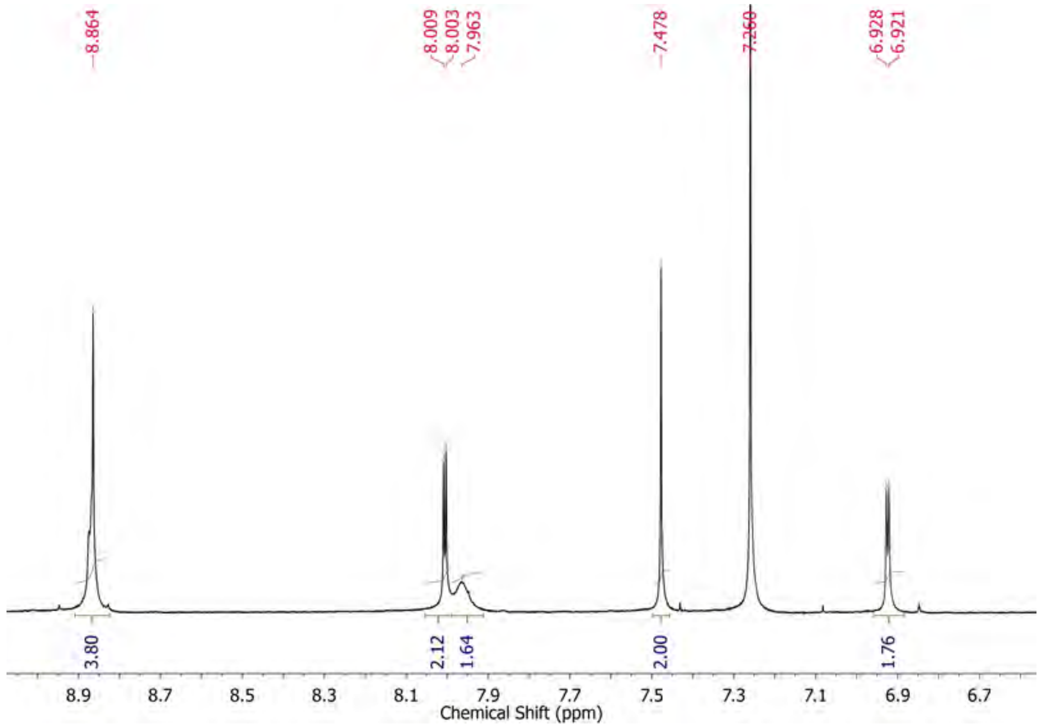

Figure S49. ^{1}H NMR ($CDCl_3$, 25°C) of **BDT-2PTTh** (aromatic region).

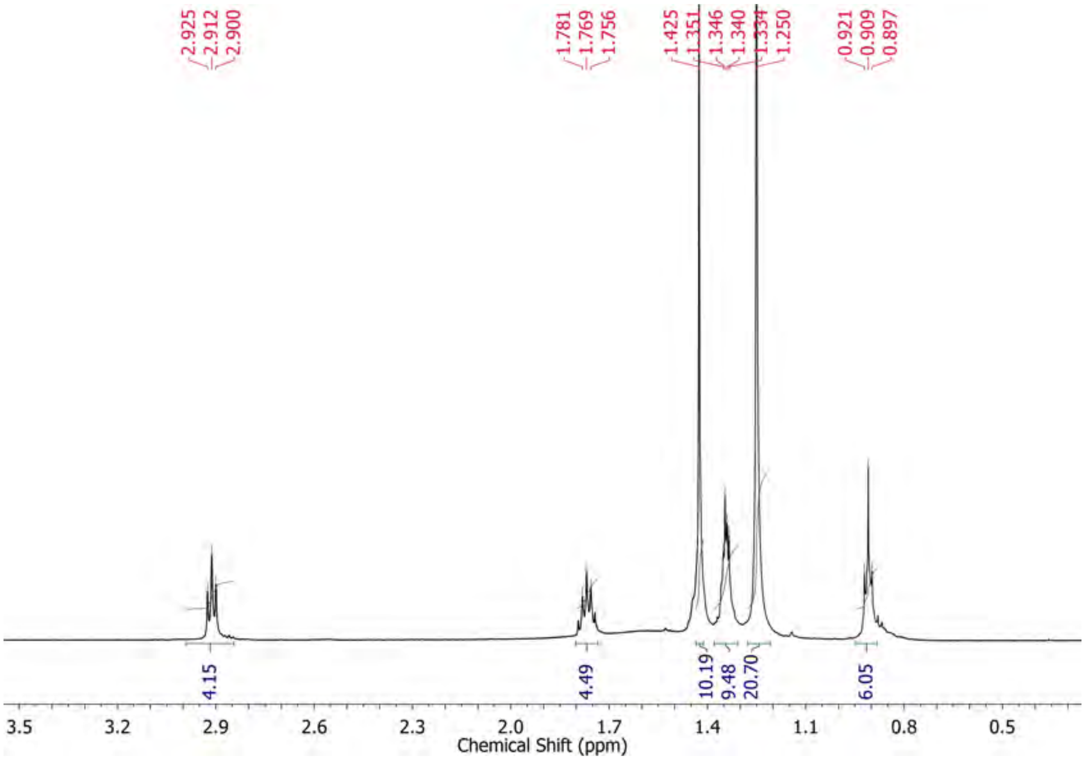

Figure S50. ^{1}H NMR ($CDCl_3$, 25°C) of **BDT-2PTTh** (aliphatic region).

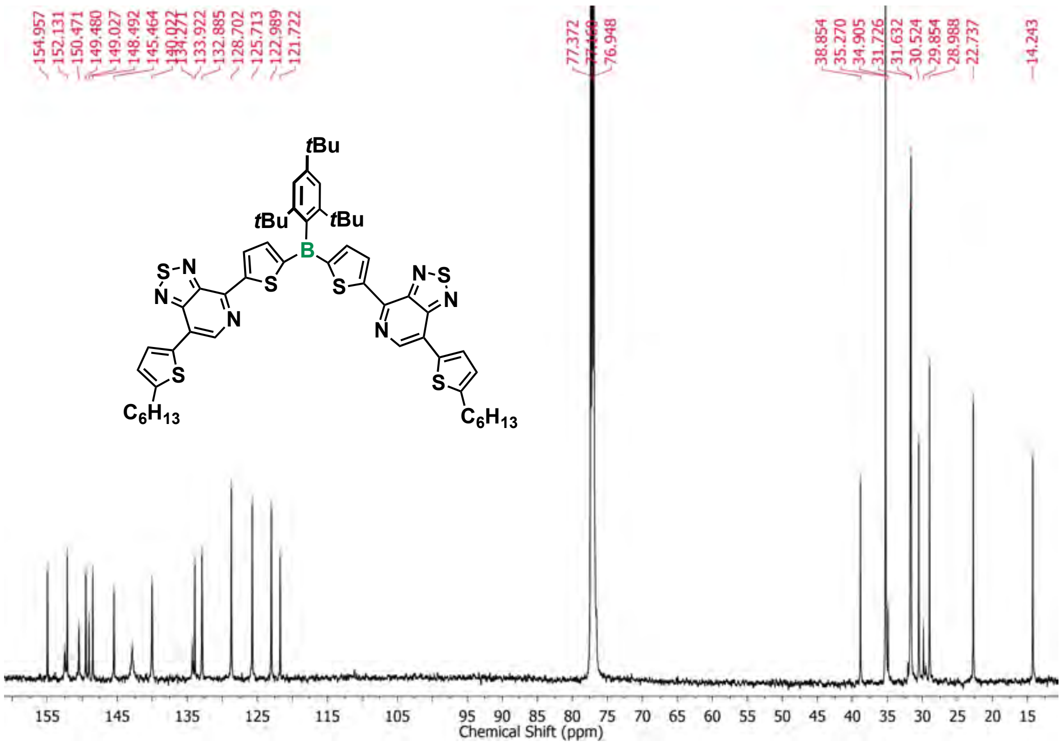

Figure S51. ^{13}C NMR ($CDCl_3$, 25°C) of **BDT-2PTTh.**

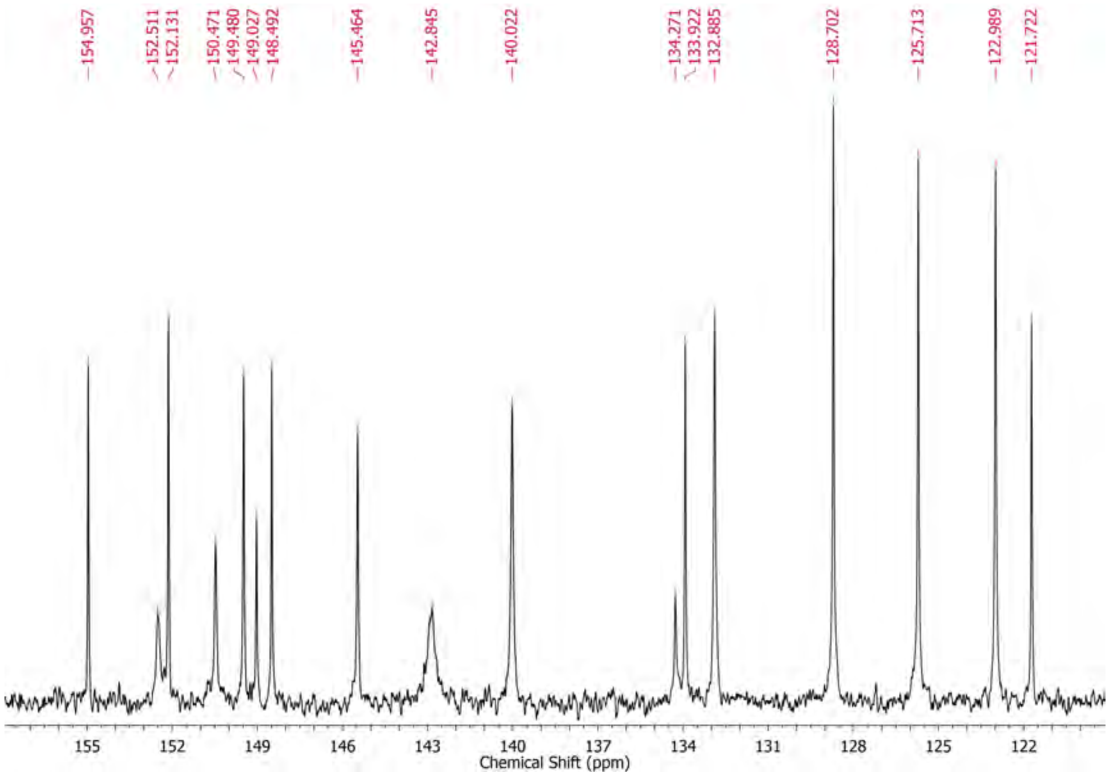

Figure S52. ^{13}C NMR ($CDCl_3$, 25°C) of **BDT-2PTTh** (aromatic region).

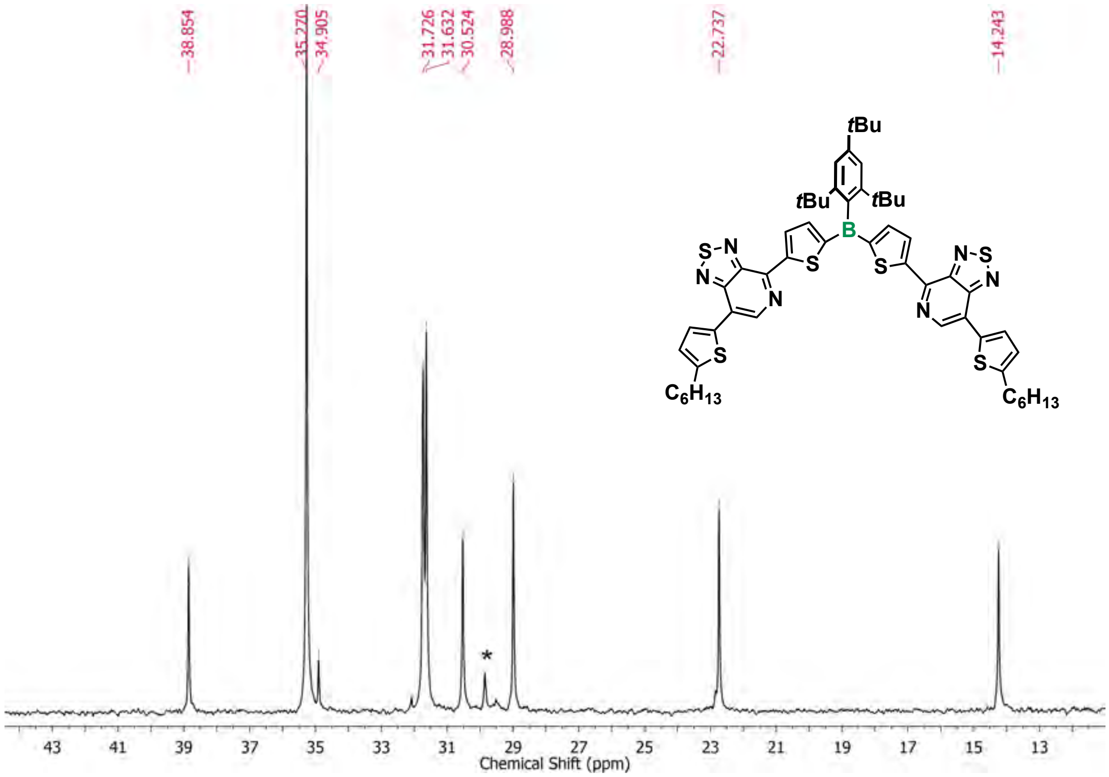

Figure S53. ^{13}C NMR ($CDCl_3$, 25°C) of **BDT-2PTTh** (aliphatic region), * residue of hexanes.

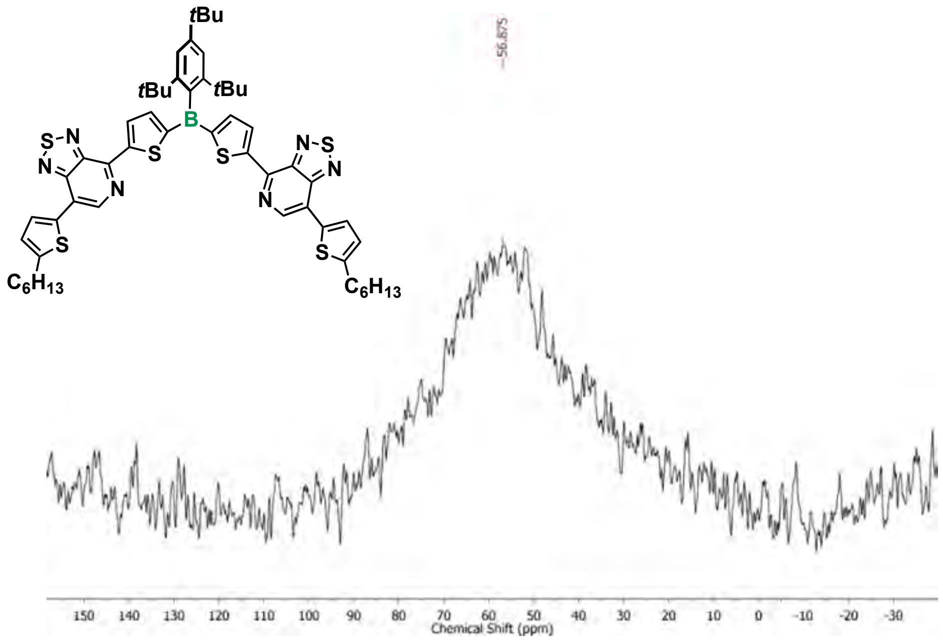

Figure S54. ^{11}B NMR ($CDCl_3$, 25°C) of **BDT-2PTTh.**

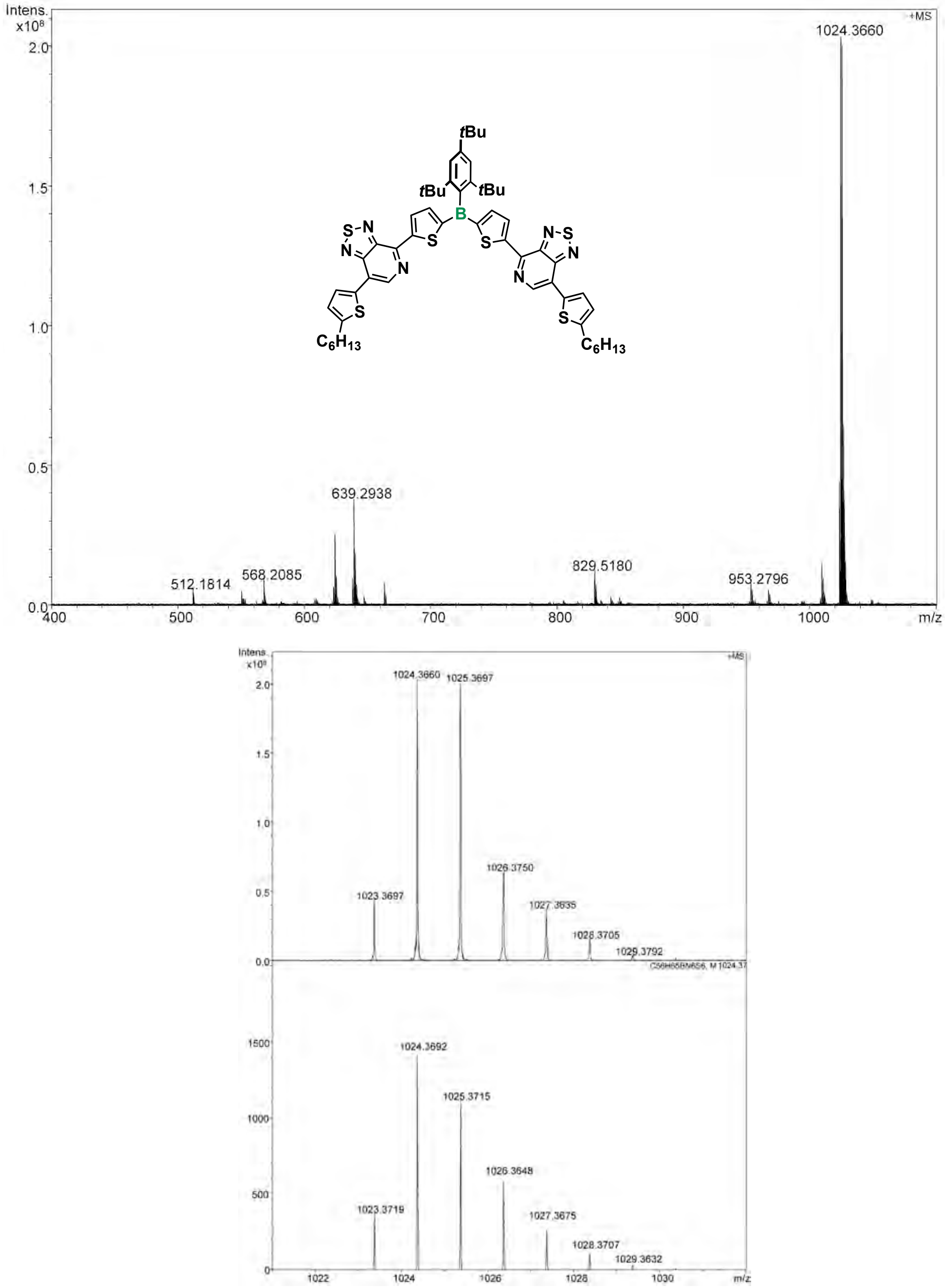

Figure S55. MALDI-MS of **BDT-2PTTh** (pos. mode).

Cartesian Coordinates for Optimized Structures

BDT-2PT; B3LYP/6-31+G* Total Energy: -8482.4175 Hatree

Element	Coordinates (angstroms)			Element	Coordinates (angstroms)		
	X	Y	Z		X	Y	Z
C	0.02286700	6.21677000	0.01630700	H	1.02096300	5.23731700	-3.61175800
C	0.00772300	3.34404700	0.01629800	H	1.12255000	3.72950500	-4.51844100
C	-0.00549000	5.48038600	-1.16702600	H	2.09609100	3.94855600	-3.05372400
C	0.04124800	5.48520800	1.19759000	B	0.00298500	1.74969800	0.01564500
C	0.01400000	4.08040100	1.24132000	C	-1.38636200	1.03645100	0.00473500
C	0.00770200	4.08068500	-1.21386900	S	-1.65565700	-0.69244500	0.03573900
H	-0.03537900	6.02563800	-2.10298700	C	-3.38991200	-0.50100100	0.00173800
H	0.07606700	6.02403000	2.13437700	C	-3.73841000	0.84035800	-0.03040700
C	0.03117800	7.75645000	-0.02584600	C	-2.61414800	1.69107100	-0.02770600
C	1.27008800	8.24552800	-0.81315800	H	-4.76577500	1.17937800	-0.05397300
H	2.19672600	7.91370500	-0.33086100	H	-2.68476600	2.77311600	-0.04511900
H	1.28532800	9.34141400	-0.86055800	C	1.38872600	1.02942900	0.02369300
H	1.27590600	7.86819700	-1.84113400	C	2.61975400	1.67786700	0.05765200
C	0.08173400	8.38425300	1.38015200	C	3.73984500	0.82169700	0.05406000
H	0.98512700	8.09011000	1.92669200	C	3.38477800	-0.51776500	0.01542200
H	-0.78945700	8.10614200	1.98423000	S	1.64953700	-0.70058200	-0.01628800
H	0.08863100	9.47686500	1.29540800	H	2.69578000	2.75943500	0.08053200
C	-1.25222500	8.25915500	-0.72880300	H	4.76887800	1.15563100	0.07748100
H	-2.14783500	7.93837700	-0.18444900	C	-4.23302000	-1.68480000	0.01111000
H	-1.33127900	7.88106700	-1.75346300	C	-5.71092100	-4.09972600	0.03272500
H	-1.25817400	9.35515800	-0.77683500	N	-3.63222400	-2.86419000	0.04642500
C	0.03330800	3.51082200	2.69960900	C	-5.67942800	-1.63334700	-0.01690800
C	1.39890600	3.86255800	3.34430600	C	-6.43031800	-2.87442800	-0.00583100
H	2.21889200	3.36761100	2.81132000	C	-4.33879800	-4.02473000	0.05705800
H	1.42462900	3.52596000	4.38827200	H	-3.74532900	-4.93396500	0.08692400
H	1.59630200	4.93902900	3.33464000	C	4.22217400	-1.70550400	-0.00259200
C	-0.16052400	1.99211300	2.86210500	C	5.68847300	-4.12724800	-0.04278000
H	0.61374500	1.40733000	2.36081200	C	5.66892400	-1.66106500	0.02057400
H	-1.13931200	1.65803300	2.51169100	N	3.61564700	-2.88182400	-0.04214100
H	-0.09563400	1.74326400	3.92838100	C	4.31664900	-4.04561100	-0.06165400
C	-1.10824200	4.15976400	3.52837800	C	6.41385000	-2.90562600	-0.00006400
H	-2.08339800	3.95403200	3.07201300	H	3.71877800	-4.95185600	-0.09438100
H	-1.00866600	5.24378200	3.62887200	N	6.45075200	-0.57889200	0.06037500
H	-1.11584900	3.73936000	4.54124100	S	8.00078800	-1.12273800	0.07024800
C	-0.01978700	3.50923500	-2.67166200	N	7.73940200	-2.74320000	0.02455900

C	-1.38738600	3.86460700	-3.30969300	N	-7.75497900	-2.70553100	-0.03441600
H	-1.58064400	4.94189700	-3.29937700	N	-6.45594400	-0.54725000	-0.05366200
H	-2.20642000	3.37321200	-2.77203300	S	-8.00849800	-1.08361900	-0.07220400
H	-1.41991600	3.52737700	-4.35329600	Br	6.58507200	-5.79016100	-0.07251300
C	0.16955900	1.98981200	-2.83319700	Br	-6.61561500	-5.75841900	0.04959200
H	-0.60409900	1.40740500	-2.32832300	H	0.09964200	1.74010300	-3.89894000
H	1.14892000	1.65368100	-2.48628200	C	1.11944600	4.15355700	-3.50711400

BDT-PT; B3LYP/6-31+G* Total Energy: -2599.1687 Hatree

Element	Coordinates (angstroms)			Element	Coordinates (angstroms)		
	X	Y	Z		X	Y	Z
C	-2.98906800	0.18535100	0.00844900	H	-1.75483300	1.10266300	-3.93954600
C	-4.68522000	-1.14450900	-1.14124700	C	-4.30717900	0.53410400	-3.50458200
C	-4.71750500	-1.04857100	1.22226400	H	-5.13647700	-0.17497100	-3.57350000
C	-3.58049400	-0.22189500	1.24470100	H	-3.98252500	0.75647800	-4.52835100
C	-3.57557700	-0.29175900	-1.20994100	H	-4.69574800	1.46080300	-3.06652100
H	-5.10450700	-1.51936000	-2.06771200	B	-1.71837300	1.14806700	-0.01584400
H	-5.16893000	-1.32048700	2.16633100	C	-0.30913900	0.46835100	-0.01168700
C	-6.51909200	-2.46265200	0.03444600	S	1.23112800	1.29569100	-0.01224100
C	-7.65867100	-1.79647400	-0.77289800	C	2.12554700	-0.20280200	-0.00257800
H	-7.95403100	-0.84334300	-0.31929000	C	1.26537900	-1.29008500	0.00104500
H	-8.54051400	-2.44860900	-0.79890500	C	-0.09175600	-0.90599300	-0.00399400
H	-7.36307800	-1.59544400	-1.80793300	H	1.61469500	-2.31412800	0.00777500
C	-7.05113400	-2.76365100	1.44901000	H	-0.91308600	-1.61411100	0.00200000
H	-7.36709900	-1.85192600	1.96902700	C	-1.98498100	2.67868800	-0.04326700
H	-6.30169800	-3.26887300	2.06906400	C	-3.23909600	3.27507900	-0.03253700
H	-7.92320600	-3.42423800	1.38231100	C	-3.22725900	4.69309500	-0.06744500
C	-6.13943700	-3.80723100	-0.63088700	C	-1.95131300	5.19889600	-0.10576800
H	-5.34105200	-4.30885800	-0.07192700	S	-0.76702600	3.94614600	-0.09924600
H	-5.79040800	-3.66886100	-1.65965100	H	-4.14720400	2.68293700	-0.00317600
H	-7.00717200	-4.47804400	-0.66073000	H	-4.11851000	5.31121400	-0.06488600
C	-3.13780900	0.17410000	2.69370900	C	3.57822600	-0.16262100	0.00144000
C	-4.24127000	1.06989400	3.31367300	C	6.39178300	0.10684200	0.00821000
H	-4.34231100	2.00482100	2.75029900	N	4.15916500	1.02733200	-0.00636000
H	-3.98586500	1.32704200	4.34940400	C	4.40688300	-1.35009000	0.01382200
H	-5.21852400	0.57693100	3.32235200	C	5.85197200	-1.20645800	0.01738200
C	-1.80802300	0.93802800	2.83938000	C	5.51216400	1.16107300	-0.00317700
H	-1.81076900	1.90085900	2.32455500	H	5.88154100	2.18224100	-0.01038700
H	-0.95326700	0.35316000	2.49259200	N	6.50864400	-2.36955000	0.02938000
H	-1.64337200	1.15235900	3.90247800	N	4.00435300	-2.62358700	0.02327200

C	-2.96882000	-1.10612200	3.55603200	S	5.36313400	-3.54366900	0.03554300
H	-2.21121000	-1.76869700	3.12155500	Br	8.31594800	0.39431800	0.01188200
H	-3.89197600	-1.68198100	3.66497600	H	-1.64772100	6.23759200	-0.13793800
H	-2.63637500	-0.83170300	4.56447500	H	-1.72251100	-1.69917200	-2.76524900
C	-3.10812800	-0.00282800	-2.67689800	H	-2.31881000	-1.17216700	-4.34946300
C	-2.60405100	-1.32949000	-3.30170100	C	-1.98394900	1.03259900	-2.86919900
H	-3.36418600	-2.11627900	-3.27648900	H	-1.05463700	0.74910800	-2.37031000

FBDT-2PT; B3LYP/6-31+G* Total Energy: -3904.7633 Hatree

Element	Coordinates (angstroms)			Element	Coordinates (angstroms)		
	X	Y	Z		X	Y	Z
C	0.03435200	3.37372600	-0.00020200	N	-3.67228900	-2.75348600	-0.32069900
C	0.38508600	5.51760800	1.15970100	C	-4.39583500	-3.89863300	-0.44742100
C	-0.25574800	5.52744300	-1.16005600	C	-6.42470900	-2.80249200	0.18175800
C	-0.27559400	4.12942600	-1.15310000	H	-3.83217100	-4.77519100	-0.75277800
C	0.36500800	4.12091900	1.15392600	N	-6.39183000	-0.53123400	0.69449200
H	0.63526400	6.05167500	2.06992200	S	-7.93049400	-1.07375200	0.85786100
H	-0.49901000	6.06797800	-2.06772300	N	-7.72407800	-2.64986900	0.45920900
C	0.14392600	7.73021500	-0.00223100	N	7.64801200	-2.82660100	-0.45785900
C	-0.69408500	3.46327900	-2.44917000	N	6.36460800	-0.67800500	-0.69266000
C	0.75959600	3.44275800	2.45140000	S	7.89060600	-1.25534100	-0.85519500
B	0.01368400	1.76556900	-0.00015200	Br	-6.70155300	-5.65520700	-0.41450700
C	1.38192100	1.05784300	-0.10949700	Br	6.55671600	-5.80836900	0.41299100
S	1.64568600	-0.64162700	0.20618500	F	-0.59444500	8.27545200	-0.99589100
C	3.34529400	-0.51991700	-0.15054400	F	-0.29537000	8.25653400	1.16601700
C	3.69168300	0.77984100	-0.49803600	F	1.41947100	8.16783900	-0.17195100
C	2.58931700	1.65687900	-0.46481800	F	-2.04416700	3.35346800	-2.54296000
H	4.70069200	1.07246500	-0.75720200	F	-0.28974300	4.17492800	-3.53088100
H	2.66948600	2.71251900	-0.70343000	F	-0.18397800	2.21847900	-2.58693200
C	-1.37073800	1.09023100	0.10931100	F	0.37365000	4.16622400	3.53182600
C	-2.56358600	1.71671800	0.46631900	F	2.10547200	3.29277900	2.54876900
C	-3.68596300	0.86536300	0.49951200	F	0.21244100	2.21355500	2.58754100
C	-3.37006300	-0.44165600	0.15020400	C	5.60195600	-1.71022800	-0.31611400
S	-1.67410700	-0.60220000	-0.20826700	C	6.34524100	-2.94976300	-0.18161900
H	-2.61903900	2.77361600	0.70653800	C	4.29131600	-4.00005600	0.44455300
H	-4.68769500	1.18089000	0.76004500	H	3.70755600	-4.86387700	0.74846800
C	4.18120300	-1.70849800	-0.04498500	C	-4.23306300	-1.61065800	0.04413200
C	5.64131600	-4.11803800	0.21310500	C	-5.74790500	-3.98617400	-0.21457600
N	3.59415400	-2.83859600	0.31819200				

FBDT-PT; B3LYP/6-31+G* Total Energy: -3138.6417 Hatree

Element	Coordinates (angstroms)			Element	Coordinates (angstroms)		
	X	Y	Z		X	Y	Z
C	3.01064800	0.12986500	0.05437200	C	-5.45378000	1.13303800	0.37469800
C	4.92254200	-0.90852400	-1.09985700	C	-5.83270700	-1.13978600	-0.26060900
C	4.51049500	-1.44565100	1.20974700	H	-5.80097700	2.11751800	0.67409400
C	3.40095700	-0.59500700	1.20256300	N	-4.01691400	-2.51525000	-0.74364600
C	3.81316600	-0.05956100	-1.09471400	S	-5.39041600	-3.39118100	-0.93060200
H	5.50600000	-1.03230600	-2.00545800	N	-6.50987600	-2.25607500	-0.55027400
H	4.77547200	-1.98303600	2.11335300	Br	-8.25812200	0.40223300	0.29725000
C	6.49819200	-2.48282300	0.06370000	F	6.42569700	-3.45087700	1.00629800
C	2.60707300	-0.52898200	2.49269600	F	6.69194800	-3.09252400	-1.12988700
C	3.53596800	0.68709300	-2.38530300	F	7.62284100	-1.76449100	0.32257100
B	1.73434600	1.10895800	0.05121000	F	1.69839400	-1.53525700	2.57259500
C	2.02198400	2.61535000	0.17622000	F	3.41003100	-0.64923200	3.57989000
S	0.84310100	3.88940300	-0.09658700	F	1.92816000	0.63123200	2.63393200
C	1.98280300	5.12794500	0.25814900	F	3.91165700	-0.03372600	-3.47135800
C	3.22076200	4.61481500	0.57481600	F	4.22100000	1.85775600	-2.44445400
C	3.23794600	3.19954800	0.51943600	F	2.22659600	0.98463900	-2.54706200
H	4.07905600	5.22772400	0.82809200	H	1.69037400	6.16950500	0.20585000
H	4.12410400	2.61003800	0.73171700	H	0.91305600	-1.58621800	-0.66627900
C	0.34816300	0.43470000	-0.08171800	H	-1.61565800	-2.23228100	-0.76341700
C	0.11137400	-0.88985700	-0.44221400	C	-3.54459300	-0.15076200	-0.08435100
C	-1.25102900	-1.24856500	-0.49824200	C	-6.35294200	0.12315500	0.12738100
C	-2.09089000	-0.19396700	-0.16497000	C	-4.39280500	-1.28696600	-0.37117100
S	-1.17574600	1.23955200	0.21025000				

BDT-2PT Fluoride Complex; B3LYP/6-31+G* Total Energy: -8582.5621 Hatree

Element	Coordinates (angstroms)			Element	Coordinates (angstroms)		
	X	Y	Z		X	Y	Z
C	-3.56185346	5.01467476	0.14407042	C	-2.16069772	-5.16407100	0.57367460
C	-1.24773149	3.27587661	0.08439283	C	-4.38350699	-5.09533469	-0.29765504
C	-3.31516404	4.09022597	1.15209476	H	-1.32211147	-5.70156836	1.00910805
C	-2.52601335	5.19555887	-0.77294345	N	-5.24214622	-3.07020775	-1.05634506
C	-1.35945590	4.41648070	-0.79596259	S	-6.39727631	-4.22493716	-1.25972199
C	-2.18047580	3.25519139	1.17786987	N	-5.58859600	-5.53596292	-0.67057257
H	-4.04204945	3.98673906	1.94666353	N	8.19571398	-0.16039712	1.13198868
H	-2.63459864	5.97831351	-1.51445553	N	6.14696886	1.21875077	1.62970687
C	-4.85241013	5.84990261	0.04809936	S	7.71621409	1.08078557	2.10539002
C	-2.09827378	2.41673882	2.50476541	Br	-3.50033447	-7.70133632	0.59268797
B	-0.44356314	1.90205683	-0.47419456	Br	8.52378128	-2.59820170	-1.01333852

C	1.09671874	1.61733699	0.03287501	F	-0.35070494	2.01363688	-1.93156770
S	2.04306927	0.37428555	-0.73809236	C	-0.28214290	4.96164278	-1.80412203
C	3.49084068	0.77004572	0.17278674	C	-0.69934067	4.68158570	-3.26834929
C	3.23053743	1.83998445	1.02744482	H	0.03127549	5.12268802	-3.96000727
C	1.90425017	2.30123740	0.93596068	H	-0.75100009	3.60765697	-3.45270728
H	3.98111564	2.26537626	1.68054128	H	-1.67846563	5.12122101	-3.49216889
H	1.53502927	3.14693587	1.50387028	C	-0.16892744	6.50733989	-1.62232351
C	-1.46619518	0.59826299	-0.36925360	H	-1.06901755	7.05258275	-1.91756375
C	-2.76918164	0.60392508	-0.86629982	H	0.05297019	6.76456210	-0.58033124
C	-3.41600863	-0.64391759	-0.85669388	H	0.65081625	6.88372133	-2.24586908
C	-2.61898700	-1.66628685	-0.34094873	C	1.15467929	4.45099044	-1.57923412
S	-1.04608804	-1.01834647	0.10520748	H	1.47424703	4.61693188	-0.54633904
H	-3.23247747	1.51308387	-1.23280127	H	1.26910141	3.39738803	-1.81361006
H	-4.42671455	-0.81104412	-1.20538148	H	1.83564394	5.01350162	-2.23064146
C	4.70469965	0.03007272	-0.03601435	C	-3.25885459	1.39527155	2.60843880
C	7.02891572	-1.51743927	-0.57789804	H	-3.17798544	0.62133427	1.84302104
N	4.70314514	-0.94976958	-0.94147221	H	-3.23859875	0.90592025	3.59088115
C	5.94197390	0.29618335	0.68989224	H	-4.23578342	1.87632661	2.49505630
C	7.12712429	-0.49965302	0.40497821	C	-2.21597038	3.40450036	3.70488732
C	5.81117552	-1.68508314	-1.19691657	H	-1.44918682	4.18461704	3.64051640
H	5.69429931	-2.45660911	-1.95350784	H	-3.18951939	3.89789010	3.76586055
C	-2.90588038	-3.06004302	-0.15619269	H	-2.06667560	2.85766529	4.64435863
C	-3.31971709	-5.84193045	0.26989164	C	-0.77709373	1.67204824	2.75153268
C	-4.18074181	-3.67085337	-0.51940036	H	-0.54074501	0.93857338	1.98739411
N	-1.96018367	-3.84039794	0.37277551	H	0.05942683	2.36819853	2.82788950
H	-5.77852041	4.57285521	-1.47226120	H	-0.84641109	1.13427325	3.70530522
H	-6.42562920	6.22720753	-1.42716199	C	-4.52285214	7.35401724	0.21267679
H	-4.84009313	5.90708604	-2.15208127	H	-3.82859585	7.70158647	-0.55848558
C	-5.88206087	5.46811837	1.13039106	H	-5.43622857	7.95915296	0.14135194
H	-6.16057570	4.41069233	1.06821653	H	-4.06020356	7.54500758	1.18738019
H	-5.50346329	5.66139793	2.14001218	C	-5.51153218	5.62636730	-1.33497958
H	-6.79384968	6.06303394	0.99948588				

BDT-PT Fluoride Complex; B3LYP/6-31+G* Total Energy: -5257.8133 Hatree

Element	Coordinates (angstroms)			Element	Coordinates (angstroms)		
	X	Y	Z		X	Y	Z
C	5.79110472	-0.82965247	0.06947167	H	1.08360605	-2.78822257	-0.25212020
C	2.94043616	-0.31893486	0.04415574	H	0.70068485	-1.75073406	-1.63605893
C	5.18737119	-0.00469817	1.01150110	H	0.89234702	-3.48956845	-1.86396859
C	4.91821699	-1.55776822	-0.73909915	C	2.98681453	-2.21041544	-3.11134489
C	3.52551365	-1.38634230	-0.73477770	H	2.50900292	-3.00594710	-3.69976017
C	3.79930547	0.23333142	1.05634483	H	2.55010946	-1.25246006	-3.39744369
H	5.81479472	0.50336524	1.73176296	H	4.05255497	-2.19940403	-3.36893067
H	5.34359461	-2.28944395	-1.41586644	C	3.94223695	2.52903762	2.23529593
C	7.31565575	-1.00636253	-0.06309067	H	3.47090099	3.09322769	1.42903640
C	2.77832298	-2.46768140	-1.59905549	H	3.74867502	3.05289192	3.18102292
C	3.38808839	1.08452922	2.31196455	H	5.02386432	2.53923220	2.06378784
B	1.59161089	0.49532488	-0.57734311	C	1.88161262	1.15889850	2.60422093
C	0.09550479	0.10996923	0.00697407	H	1.30659652	1.60318872	1.79817408
S	-1.32017674	0.68545873	-0.82868306	H	1.47350723	0.16859881	2.81487334
C	-2.42293824	-0.19961369	0.21414826	H	1.72255378	1.77687218	3.49713038
C	-1.69461390	-0.91992758	1.16185628	C	3.99562883	0.40355677	3.57635409
C	-0.30616226	-0.74257388	1.03285600	H	3.68825912	-0.64652353	3.63764770
H	-2.16276758	-1.55101525	1.90584608	H	5.08822412	0.43367584	3.60141989
H	0.41223799	-1.25265373	1.66312490	H	3.63394900	0.91730499	4.47611773
C	1.90628658	2.11693683	-0.63393588	C	7.75077566	-0.66386173	-1.50907951
C	3.03731350	2.68141333	-1.18750903	H	8.83529787	-0.78943377	-1.62616849
C	3.02153621	4.10561741	-1.29164239	H	7.25584544	-1.30830288	-2.24191670
C	1.85973619	4.65475350	-0.81061465	H	7.49664173	0.37290673	-1.75501553
S	0.78433109	3.41262084	-0.25093315	C	7.70888139	-2.47166241	0.24734924
H	3.87302118	2.07156957	-1.51468701	H	7.22050074	-3.17325057	-0.43598304
H	3.83728649	4.69385294	-1.70186659	H	8.79424456	-2.60892275	0.15254147
C	-3.84061878	-0.11595742	0.01209427	H	7.41671331	-2.74394022	1.26770568
C	-6.62325331	0.15240698	-0.52033656	C	8.10133553	-0.09007953	0.89619051
N	-4.29826166	0.64544575	-0.98579759	H	7.87287596	0.96691031	0.72210019
C	-4.81387488	-0.82156611	0.84141082	H	7.88487611	-0.31615673	1.94602991
C	-6.23602023	-0.67832523	0.56143901	H	9.17829178	-0.23037013	0.74280883
C	-5.62211107	0.76998837	-1.23581811	H	1.57600685	5.69792180	-0.76115320
H	-5.87970545	1.41447225	-2.07253178	F	1.52122581	0.16755671	-2.00446335
N	-7.02438622	-1.37147728	1.38804288	C	3.37081924	-3.87066578	-1.26033124
N	-4.56504498	-1.62202673	1.87691584	H	2.79225798	-4.64172136	-1.78350894
S	-6.01955001	-2.14613190	2.44259673	H	4.41395585	-3.99372163	-1.56361410
Br	-8.45316072	0.39582762	-0.95470587	H	3.30850943	-4.07304406	-0.18486675
C	1.27082925	-2.61676886	-1.31630782				

FBDT-2PT-Fluoride Complex; B3LYP/6-31+G* Total Energy: -4004.7490 Hatree

Element	Coordinates (angstroms)			Element	Coordinates (angstroms)		
	X	Y	Z		X	Y	Z
C	1.34279200	6.05856600	0.40114600	N	3.05979800	-3.26525000	0.33492700
C	0.46710800	3.37448700	-0.17032300	C	5.16784300	-2.61398100	-0.60197700
C	1.73515100	5.00186200	1.21191500	C	5.71654500	-3.92871700	-0.29555500
C	0.44231200	5.81182000	-0.63042100	C	3.57814100	-4.48665500	0.61526300
C	-0.00709200	4.51263400	-0.89019000	H	2.89448500	-5.18174300	1.09649000
C	1.29072100	3.70138200	0.94280000	C	-4.43316700	-1.05269700	-0.01328000
H	2.38247500	5.18574400	2.06165700	C	-6.56152200	-2.84461500	-0.59091000
H	0.07320600	6.63783500	-1.22518400	C	-5.48594400	-1.27283800	0.97190800
C	1.88806000	7.43777000	0.63280600	N	-4.50546900	-1.71027800	-1.17075900
C	-1.06802300	4.45424000	-1.99055300	C	-5.52176600	-2.56535000	-1.44567500
C	1.78745600	2.69481400	1.96945100	C	-6.57402900	-2.19314400	0.66793700
C	1.49469100	0.85780800	-0.64458800	H	-5.47475300	-3.04449100	-2.42052400
S	1.48748800	-0.76987700	-0.05448200	N	-5.59586700	-0.71689300	2.17869700
C	3.16000800	-1.04318200	-0.51671400	S	-6.96512600	-1.31650100	2.86220700
C	3.67791700	0.10390600	-1.11666300	N	-7.47406100	-2.30791700	1.64922100
C	2.74183600	1.15354500	-1.18605500	N	6.99342600	-4.07904900	-0.66033600
H	4.69406300	0.16637500	-1.48509700	N	6.04921600	-1.80600400	-1.19140400
H	2.96347300	2.11902300	-1.63117500	S	7.45173500	-2.65034200	-1.34054200
C	-1.18811200	1.16958000	-0.09482300	Br	-7.97382100	-4.09127500	-1.08025800
C	-1.82491000	1.37238300	1.12442500	Br	5.51651300	-6.65260900	0.78067900
C	-3.01262200	0.63302700	1.30034400	F	1.04797600	8.41277500	0.20031900
C	-3.32150400	-0.16343200	0.19994500	F	2.13619100	7.68460400	1.94615600
S	-2.10464000	0.04278600	-1.04855600	F	3.07120700	7.64037100	-0.01801500
H	-1.43943200	2.04820900	1.87993000	F	-2.02108900	3.53207100	-1.78897100
H	-3.62911200	0.67460100	2.18934000	F	-1.74410100	5.65521800	-2.04653600
C	3.78309600	-2.31318800	-0.25590400	F	-0.55428300	4.29134800	-3.23210900
C	4.86734000	-4.87288300	0.33412100	F	1.80678800	3.26178600	3.21993800
B	0.16452300	1.81771900	-0.74031300	F	3.06657900	2.29456700	1.74136700
F	-0.03097600	1.93601900	-2.16710800	F	1.02884000	1.59342400	2.08611000

FBDT-PT Fluoride Complex; B3LYP/6-31+G* Total Energy: -3238.6147 Hatree

Element	Coordinates (angstroms)			Element	Coordinates (angstroms)		
	X	Y	Z		X	Y	Z
C	-5.43242600	-1.45844300	0.28274900	C	4.57610100	-0.54986000	1.08922700
C	-2.94226600	-0.10078700	-0.22435100	N	4.16806100	0.26271400	-1.12968500
C	-5.04814200	-0.37650300	1.06310900	C	5.49612300	0.18204100	-1.38726600
C	-4.54416700	-1.94097600	-0.67480000	C	6.00320500	-0.63225800	0.80238600
C	-3.32197500	-1.30059300	-0.90047800	H	5.79458400	0.48650500	-2.38767000
C	-3.82446900	0.26492600	0.82933100	N	4.27129500	-0.93565500	2.32788800
H	-5.69389200	-0.03075900	1.86188200	S	5.67966200	-1.36490600	3.06034100
H	-4.79779500	-2.83167000	-1.23676800	N	6.73567200	-1.07627600	1.82819600
C	-6.77737500	-2.09926600	0.45752100	Br	8.33246300	-0.32344400	-0.95861700
C	-2.43916800	-2.03513700	-1.90984100	F	-6.74396200	-3.44472200	0.26300300
C	-3.55460400	1.37590100	1.83301300	F	-7.30047600	-1.89472900	1.69446600
B	-1.66365100	0.83781700	-0.80537600	F	-7.69943200	-1.61690800	-0.42750500
C	-2.00981400	2.43628600	-0.77400100	F	-1.12458000	-1.95653900	-1.65725200
S	-0.90968700	3.66721100	-0.19521300	F	-2.72452100	-3.38607800	-1.87434000
C	-1.93511400	4.96739300	-0.71041700	F	-2.66151400	-1.67283300	-3.19506200
C	-3.07059400	4.48396800	-1.31089700	F	-3.98939600	0.99343900	3.08251400
C	-3.10118400	3.05587000	-1.34562700	F	-4.23425400	2.51958200	1.56082800
H	-3.85535100	5.11926200	-1.71319000	F	-2.25990900	1.69115700	1.98910600
H	-3.91521700	2.49190600	-1.79355500	H	-1.64887800	5.99916600	-0.54666100
C	-0.23521000	0.45055100	-0.10803400	F	-1.56295000	0.52442300	-2.21472200
C	0.08878600	-0.02554600	1.15937600	H	-0.65909700	-0.20324500	1.92423100
C	1.46182100	-0.26128900	1.36856200	H	1.88085900	-0.63374500	2.29483500
C	2.23858200	0.03036600	0.24754700	C	3.65855000	-0.08004100	0.05553700
S	1.20977500	0.59295400	-1.06061900	C	6.44411200	-0.24605500	-0.48778900

BDT-2PTTh; B3LYP/6-31+G* Total Energy: -4521.9660 Hatree

Element	Coordinates (angstroms)			Element	Coordinates (angstroms)		
	X	Y	Z		X	Y	Z
C	0.01539200	6.18365300	-1.17226900	C	-6.44825300	-2.15062100	-0.01235100
C	0.02267200	4.78256900	-1.21743400	C	-5.68766100	-0.91310200	-0.01834300
C	0.02007900	4.04722000	0.01461100	N	-6.45281400	0.18317000	-0.04725900
C	0.02661600	4.78509100	1.23957400	S	-8.01114100	-0.32654400	-0.06543100
C	0.05267700	6.19133100	1.19390300	C	-5.76163200	-3.41454600	0.01771300
C	0.04006000	6.92249800	0.01088900	C	-4.37395200	-3.29939500	0.03961900
B	0.01157900	2.45119700	0.01569300	N	-3.65668800	-2.15320700	0.03378600
C	-1.37983000	1.74486400	0.00440100	C	-4.24417700	-0.96437500	0.00556900
C	1.39570800	1.73074400	0.02592900	H	3.71951900	-4.23676600	-0.06269500
C	2.62847300	2.37856000	0.05612000	H	4.77714100	1.85724200	0.07602200
C	3.74845600	1.52159400	0.05511100	H	2.70721000	3.46066700	0.07519800
C	3.39326200	0.17898300	0.02274000	H	-3.76371100	-4.19913400	0.06416800
S	1.65670100	0.00031000	-0.00611600	H	-4.75978000	1.90573900	-0.04947600
C	0.05104100	8.46319500	-0.03250500	H	-2.67357600	3.48818700	-0.04031600
C	-0.00315200	4.21059000	-2.67707500	C	1.15249900	4.83871400	-3.50396300
C	0.04345400	4.21858100	2.70107500	H	1.07073700	5.92450800	-3.60709700
S	-1.65840600	0.01703800	0.03025000	H	1.15592300	4.41588500	-4.51644500
C	-3.39299000	0.21345400	-0.00054000	H	2.12321400	4.61799000	-3.04419200
C	-3.73455200	1.55969300	-0.02835100	C	-1.35979200	4.58752700	-3.32887600
C	-2.60593100	2.40526400	-0.02502800	H	-2.19232600	4.11319900	-2.79602300
N	7.75092500	-2.02892900	0.03822000	H	-1.38807600	4.24435900	-4.37128400
C	6.42466700	-2.21562300	0.01589100	H	-1.53481300	5.66836400	-3.32749800
C	5.67654100	-0.97059000	0.03024200	C	0.16097400	2.68751000	-2.84107200
N	6.45272700	0.11779500	0.06258200	H	-0.63013400	2.11663100	-2.35016100
S	8.00591400	-0.40759500	0.07386200	H	1.12990100	2.33100900	-2.48432500
C	5.72534100	-3.47245500	-0.01867800	H	0.10124300	2.44428100	-3.90960000
C	4.33882900	-3.34331500	-0.03565100	C	-1.22813200	8.97030700	-0.74216000
N	3.63309900	-2.19005600	-0.02222000	H	-2.12798700	8.64939100	-0.20379600
C	4.23253300	-1.00730600	0.00988000	H	-1.30160100	8.59519700	-1.76873100
N	-7.77250600	-1.95050100	-0.03734400	H	-1.23133900	10.06711800	-0.78695100
H	1.42135800	4.24879100	4.40085200	C	1.29471800	8.95238800	-0.81415400
C	-0.13059400	2.69720800	2.87000800	H	1.31230800	10.04909700	-0.85689800
H	-0.07674500	2.45743600	3.93964100	H	1.30303800	8.57926100	-1.84404900
H	0.65904500	2.11998000	2.38410300	H	2.21932200	8.61608800	-0.32979900
H	-1.10024800	2.34526800	2.51075500	C	0.09577000	9.09423200	1.37323400
C	-1.11130800	4.85761200	3.52090000	H	-0.77939900	8.82043000	1.97447800
H	-1.02458500	5.94369200	3.61635400	H	0.10456900	10.18717700	1.28440300
H	-1.11923900	4.44153700	4.53618600	H	0.99685000	8.80174600	1.92552700
H	-2.08188300	4.63788500	3.06023700	C	1.40010500	4.58867700	3.35723200

H	0.08319800	6.73198400	2.12997000	H	1.58312400	5.66812300	3.35336800
H	-0.00796100	6.72800500	-2.10914500	H	2.23141100	4.10637300	2.82957800
C	-6.44542500	-4.69859200	0.02430700	C	8.03908400	-6.43975100	-0.04893800
C	-7.80053500	-4.96869300	0.01257300	C	7.74860000	-5.04686200	-0.02824600
C	-8.10493400	-6.35869800	0.02444900	H	9.04844100	-6.84020900	-0.04752100
C	-6.99873400	-7.17150000	0.04504400	H	8.50358700	-4.27222700	-0.00934300
S	-5.53974400	-6.21334600	0.05019200	C	-6.94110500	-8.67003300	0.06188800
H	-8.54773800	-4.18648500	-0.00415100	H	-7.95709500	-9.07802200	0.05324900
H	-9.11820500	-6.74910400	0.01778800	H	-6.43277200	-9.04956900	0.95719500
C	6.39623300	-4.76321100	-0.03440100	H	-6.41009200	-9.06862200	-0.81169700
S	5.47541600	-6.26867400	-0.06627900	C	6.85194600	-8.73909600	-0.09623700
C	6.92477300	-7.24135100	-0.07092800	H	6.33683300	-9.10824300	-0.99201300
H	7.86373900	-9.15748700	-0.09337500	H	6.31978200	-9.13735700	0.77679900

S4. References

S1 X. Yin, J. Chen, R. A. Lalancette, T. B. Marder and F. Jäkle, *Angew. Chem. Int. Ed.*, 2014, **53**, 9761-9765.

S2 X. Yin, F. Guo, R. A. Lalancette and F. Jäkle, *Macromolecules*, 2016, **49**, 537-546.

S3 Gaussian 09, Revision B.01, M. J. Frisch, G. W. Trucks, H. B. Schlegel, G. E. Scuseria, M. A. Robb, J. R. Cheeseman, G. Scalmani, V. Barone, B. Mennucci, G. A. Petersson, H. Nakatsuji, M. Caricato, X. Li, H. P. Hratchian, A. F. Izmaylov, J. Bloino, G. Zheng, J. L. Sonnenberg, M. Hada, M. Ehara, K. Toyota, R. Fukuda, J. Hasegawa, M. Ishida, T. Nakajima, Y. Honda, O. Kitao, H. Nakai, T. Vreven, J. A. Montgomery, Jr., J. E. Peralta, F. Ogliaro, M. Bearpark, J. J. Heyd, E. Brothers, K. N. Kudin, V. N. Staroverov, T. Keith, R. Kobayashi, J. Normand, K. Raghavachari, A. Rendell, J. C. Burant, S. S. Iyengar, J. Tomasi, M. Cossi, N. Rega, J. M. Millam, M. Klene, J. E. Knox, J. B. Cross, V. Bakken, C. Adamo, J. Jaramillo, R. Gomperts, R. E. Stratmann, O. Yazyev, A. J. Austin, R. Cammi, C. Pomelli, J. W. Ochterski, R. L. Martin, K. Morokuma, V. G. Zakrzewski, G. A. Voth, P. Salvador, J. J. Dannenberg, S. Dapprich, A. D. Daniels, O. Farkas, J. B. Foresman, J. V. Ortiz, J. Cioslowski, and D. J. Fox, Gaussian, Inc., Wallingford CT, 2010.
